# PCYT2 inhibits epithelial-mesenchymal transition in colorectal cancer by elevating YAP1 phosphorylation

**DOI:** 10.1172/jci.insight.178823

**Published:** 2024-12-20

**Authors:** Lian Zhou, Su Zhang, Lingli Wang, Xueqin Liu, Xuyang Yang, Lei Qiu, Ying Zhou, Qing Huang, Yang Meng, Xue Lei, Linda Wen, Junhong Han

**Affiliations:** 1 Department of Biotherapy, Cancer Center and State Key Laboratory of Biotherapy, and Frontiers Science Center for Disease-related Molecular Network, and; 2 Department of Gastrointestinal Surgery, West China Hospital, Sichuan University, Chengdu, China.

**Keywords:** Cell biology, Oncology, Cancer, Colorectal cancer, Tumor suppressors

## Abstract

Metabolic reprogramming is a common feature in tumor progression and metastasis. Like proteins, lipids can transduce signals through lipid-protein interactions. During tumor initiation and metastasis, dysregulation of the Hippo pathway plays a critical role. Specifically, the inhibition of YAP1 phosphorylation leads to the relocation of YAP1 to the nucleus to activate transcription of genes involved in metastasis. Although recent studies reveal the involvement of phosphatidylethanolamine (PE) synthesis enzyme phosphoethanolamine cytidylyltransferase 2 (PCYT2) in tumor chemoresistance, the effect of PCYT2 on tumor metastasis remains elusive. Here, we show that PCYT2 was significantly downregulated in metastatic colorectal cancer (CRC) and acted as a tumor metastasis suppressor. Mechanistically, PCYT2 increased the interaction between PEBP1 and YAP1–phosphatase PPP2R1A, thus disrupting PPP2R1A-YAP1 association. As a result, phosphorylated YAP1 levels were increased, leading to YAP1 degradation through the ubiquitin protease pathway. YAP1 reduction in the nucleus repressed the transcription of ZEB1 and SNAIL2, eventually resulting in metastasis suppression. Our work provides insight into the role of PE synthesis in regulating metastasis and presents PCYT2 as a potential therapeutic target for CRC.

## Abstract 2

Suppression of EMT via YAP1 phosphorylation manipulated by PCYT2

## Graphical abstract

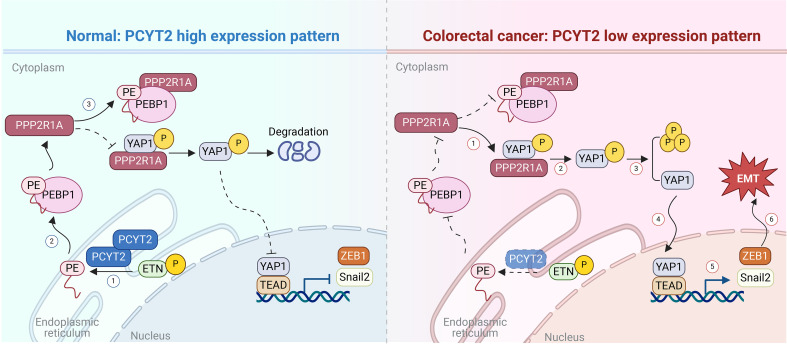

## Introduction

Colorectal cancer (CRC) is the most common gastrointestinal malignancy. CRC is the third most prevalent cancer worldwide and the second leading cause of cancer-related deaths, with a mortality rate of approximately 9.3% (1). Despite advancements in diagnostic and therapeutic techniques, prognoses remain poor due to distant metastasis, which is the major cause of death in patients with CRC (2). Cancer exhibits altered metabolism compared with normal tissues, including in the production of CO_2_, lactic acid, aspartic acid, asparagine, oxaloacetic acid, and other organic acids (3–6). Metabolic reprogramming is a common feature in cancer progression and metastasis (5, 7–10). While lipid composition has been shown to be altered in some cancers during metastasis, the precise mechanistic contributions remain unknown.

Phosphatidyl ethanolamine (PE) is an essential component of biological membranes with keys role in cell functions (11, 12). The majority of PE is produced via the *Kennedy* pathway, which involves the stepwise conjugation of ethanolamine to PE in the endoplasmic reticulum (13). The synthesis of PE is catalyzed by phosphoethanolamine cytidylyltransferase 2 (PCYT2), which is ubiquitously expressed in most cells and tissues but aberrantly regulated in some cancers (14). Other genes required for PE biosynthesis are ethanolamine kinase 1 and 2 (ETNK1 and ETNK2) (15, 16). Pcyt2 is reported to be essential for embryonic development in mice, and *Pcyt2^–/–^* complete KO is embryonically lethal (17). Pcyt2 and the PE synthesis pathway regulate phospholipid remodeling and promote the transition from mesenchymal to epithelial cells, thus allowing cell pluripotency (18). Recent studies have also revealed the involvement of PCYT2 and PE in tumor drug resistance and glutamine starvation (19, 20). The data suggest that targeting the synthesis of PE by manipulating PCYT2 expression is a potential therapeutic approach to treat cancer. However, whether and how PCYT2 and the PE synthesis pathway influence cancer metastasis remains unclear. Focusing on PCYT2 enzymatic activity, its effect on the PE synthesis pathway, and its regulation roles in tumorigenesis and metastasis will clarify the effect of phospholipid metabolism in cancer and may provide clinical opportunities for PCYT2-targeted therapy.

Here, we decipher the mechanisms through which PCYT2 functions as a master inhibitor of epithelial-mesenchymal transition (EMT) by changing YAP1 stability and accumulation in the nucleus. Briefly, the key rate-limiting enzyme PCYT2 regulates the *Kennedy* pathway for de novo synthesis of PE. Then, PE binds to PE binding protein 1 (PEBP1), which acts as a rheostat for the specificity of the PP2A phosphatase complex member PPP2R1A. This PE-PEPB1 interaction inhibits the catalytic competence of PPP2R1A toward YAP1 and leads to the degradation of YAP1. Insufficient YAP1 in the nucleus due to its degradation attenuates the transcription of mesenchymal genes, such as ZEB1 and SNAIL2, eventually resulting in suppression of EMT and metastasis. With these data, we can uncover the relevance of PE synthesis mechanisms affecting the Hippo signaling pathway to the pathophysiology of cancer metastasis and provide insight into PCYT2 as a potential therapeutic target for CRC.

## Results

### Downregulation of PCYT2 correlates with poor prognosis in CRCs.

**Figure 1:**
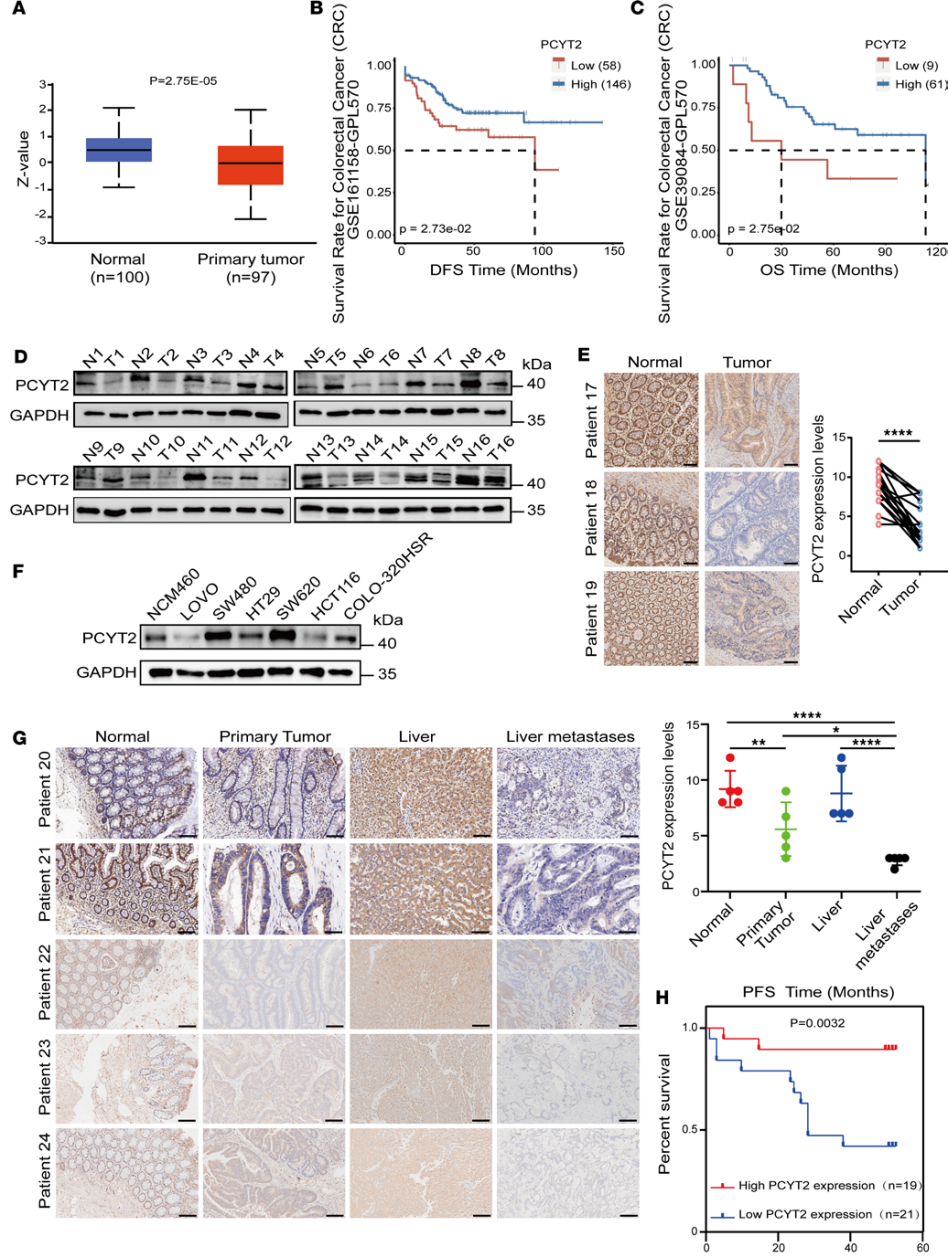
The downregulation of PCYT2 positively correlates with poor prognosis in colorectal cancer. (**A**) The relative protein expression of PCYT2 in public Clinical Proteomic Tumor Analysis Consortium (CPTAC) datasets exhibited a lower level in primary tumors compared with normal tissues. (**B** and **C**) Disease-free survival (DFS) (**B**) and Overall survival (OS) (**C**) of colorectal cancer patients in CPTAC datasets. (**D**) Detection of PCYT2 expression in 16 paired CRC tissues (T) and the corresponding adjacent normal tissues (N) by Western blot. (**E**) Representative images of PCYT2 immunohistochemical detection in paired patients with CRC tissues and their normal tissues. Representative images and quantification. Scale bar: 100 μm. *P* value was calculated with 2-tailed paired Student’s *t* test (****P < 0.0001). (**F**) Detection of PCYT2 expression in CRC cell lines and the normal intestinal epithelial cell line NCM460 by Western blot. (**G**) Representative images of PCYT2 IHC detection in patients with CRC paired with primary tumor, normal tissues, liver, and liver metastases. Representative images and quantification. Scale bar: 50 μm. *P* value was calculated with 1-way ANOVA with Tukey’s multiple-comparison test (*P < 0.05, **P < 0.01, ****P < 0.0001). (**H**) Estimation of the relationship between PCYT2 expression and PFS times in 40 patients with CRC by Kaplan-Meier analysis. *P* value was calculated with log-rank test (*P* = 0.0032).

To evaluate the potential role of PCYT2, a key rate-limiting enzyme of PE synthesis pathway in CRC progression, we analyzed PCYT2 protein expression difference between tumor and normal tissue in the publicly available datasets. We found a significant reduction in PCYT2 expression in CRC (Figure 1A) and pancancer (Supplemental Figure 1; supplemental material available online with this article; https://doi.org/10.1172/jci.insight.178823DS1). We also observed that low levels of PCYT2 protein in the datasets positively correlated with poor disease-free survival (DFS) and overall survival (OS) (Figure 1, B and C). To further verify PCYT2 expression, we collected 33 paired samples of human CRC tissue and corresponding normal mucosa tissue from West China Hospital, Sichuan University. Western blot and IHC showed significantly lower PCYT2 expression in most CRC samples compared with expression in paired adjacent normal tissues (Figure 1, D and E). We also examined the expression of PCYT2 in CRC cell lines (Lovo, SW480, HCT116, SW620, COLO320, and HT29) and the normal colon cell line NCM460 and observed the highest expression in SW480 and SW620 cells compared with lower expression levels in HCT116 and HT29 cells (Figure 1F). To explore the role of PCYT2 in CRC metastasis, we examined PCYT2 expression in patients with CRC with liver metastases, as the liver is the most common metastasis site in CRC. Intriguingly, IHC revealed significantly lower expression of PCYT2 in live metastatic tumor compared with primary tumor (Figure 1G), implying the possible involvement of PCYT2 in CRC liver metastasis. This possibility is supported by the results of IHC staining in 40 CRC patient tissues, which indicated that patients with CRC with expression of PCYT2 (*n* = 21) had shorter progression-free survival (PFS) times, whereas patients with high PCYT2 expression (*n* = 19) had longer PFS times (Figure 1H). Collectively, these results indicate that PCYT2 is critical for tumor development and metastasis and may serve as a tumor suppressor in CRC.

### PCYT2 inhibits CRC migration and EMT in vitro via its cytidine transferase domain.

**Figure 2:**
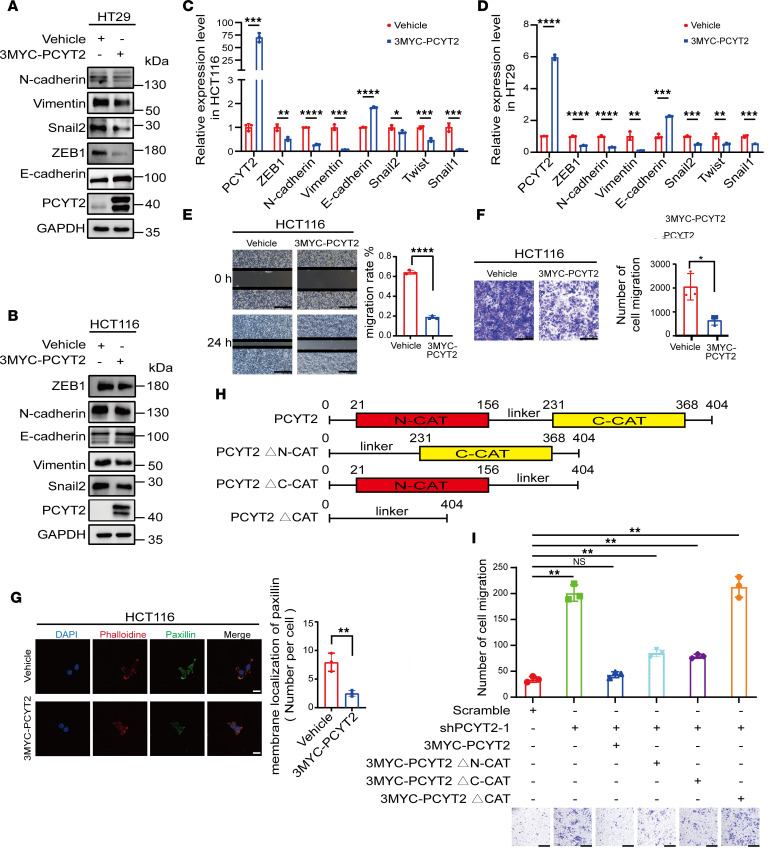
Inhibition of CRC cell migration and EMT in vivo and in vitro by PCYT2 overexpression. (**A** and **B**) Detection of epithelial and mesenchymal markers in PCYT2-overexpression HT29 (**A**) and HCT116 (**B**) cells by Western blot. (**C** and **D**) Transcriptional alteration of epithelial and mesenchymal marker genes in PCYT2-overexpressed HCT116 (**C**) and HT29 (**D**) cells detected by quantitative PCR. (**E**) Representative images of scratch assays in PCYT2-overexpression HCT116 cells and control group. The graph shows the area of a wound evaluated with ImageJ (NIH). Representative images and quantification. Scale bar: 50 μm. (**F**) Investigation of the cell migratory and invasion ability of PCYT2-overexpression HCT116 cells by Transwell assay. Representative images and quantification. Scale bar: 100 μm. (**G**) Representative images of immunofluorescence assays for the membrane localization of paxillin in HCT116 cells with ectopic expression of PCYT2. Different colors represent different antibodies or dyes: DAPI (blue), paxillin (green), and phalloidine (red). Representative images and quantification. Scale bar: 20 μm. (**H**) Schematic diagram of WT PCYT2 and cytidine transferase catalytic domain deletion mutants used in the study. (**I**) Representative images and quantification of Transwell assays for shPCYT2 cells with ectopic expression of WT PCYT2 or deletion of the cytidine transferase catalytic domain of PCYT2. Statistical chart and representative images. Scale bar: 100 μm. Data represent the mean ± SD (*n* = 3). Statistical significance was assessed with 2-tailed unpaired Student’s *t* test (**C**–**G**) or 1-way ANOVA with Tukey’s multiple-comparison test (**I**). *P < 0.05, **P < 0.01, ***P < 0.001, ****P < 0.0001.

EMT is considered an essential determinant in cancer progression (21). EMT refers to the reversible biological process by which epithelial cells are transformed into mesenchymal phenotypes through specific procedures (22). EMT is driven by Snail1, Snail2, zinc-finger E-box-binding (ZEB), and 2-handed E box binding zinc finger protein SIP1 and basic helix–loop–helix (bHLH) transcription factors, which repress epithelial marker genes and activate genes associated with the mesenchymal phenotype (23–28). To ascertain the function of PCYT2 expression on the metastasis of CRC cells, we constructed HCT116 and HT29 cells stably overexpressing PCYT2 and found that overexpression of PCYT2 led to upregulation of the epithelial marker E-cadherin but downregulation of mesenchymal markers (N-cadherin and Vimentin) and key transcription factors (Snail1, Twist, Snail2, and ZEB1) (Figure 2, A–D). Meanwhile, PCYT2 knockdown in SW480 and SW620 cells decreased epithelial marker expression and increased mesenchymal marker expression (Supplemental Figure 2, A–C). To investigate cell migration, we employed scratch wound and Transwell assays and observed a noticeable reduction in the migratory ability of HCT116 cells stably expressing PCYT2 (Figure 2, E and F). In contrast, the migration abilities were improved in PCYT2-silenced CRC cells (Supplemental Figure 2, D and E). To further examine migration capabilities, we examined the state of focal adhesion formation by measuring paxillin clustering levels. Paxillin is a core component of focal adhesion complexes, and its clustering is a characteristic of focal adhesion formation (29). In HCT116 cells stably overexpressing PCYT2, the number of paxillin fluorescence spots were significantly reduced compared with control (Figure 2G). PCYT2-deficient SW480 cells showed a significant increase in the number of paxillin fluorescence spots (Supplemental Figure 2F), indicating the critical role of PCYT2 in the regulation of CRC migration. Overexpression of PCYT2 increased PE levels, while knockdown of PCYT2 decreased PE levels, indicating that PCYT2 plays a key rate-limiting role in the PE synthesis pathway (Supplemental Figure 2, G–J). Intriguingly, PCYT2 knockdown did not affect the proliferation of CRC cells (Supplemental Figure 3, A–D).

The PCYT2 protein contains 2 catalytic domains responsible for cytidyl transferase activity: the C-terminal cytidyl transferase domain and the N-terminal cytidyl transferase domain (30). We generated a mutant variant of PCYT2 lacking the functional cytidyl transferase catalytic domain (Figure 2H) to investigate this domain on the migratory capacity of CRC cells. First, we observed that exogenous overexpression of PCYT2 in PCYT2-knockdown HCT116 cells restored their migration ability (Figure 2I). However, only partial restoration of migration ability was observed in PCYT2-knockdown HCT116 cells upon transfection with mutants lacking either the C-terminal transferase domain or the N-terminal transferase domain (Figure 2I). Conversely, no change in migration ability was observed in PCYT2-knockdown HCT116 cells when they were transfected with mutants with deletions in both the C-terminal and N-terminal transferase domains (Figure 2I). Collectively, these results suggest that PCYT2 inhibits the metastasis of CRC in vitro, and the inhibitory effect of PCYT2 on the metastasis of CRC cells in vitro is mediated by its cytidine transferase domain.

### The overexpression of PCYT2 suppresses the metastasis of CRC in vivo.

**Figure 3:**
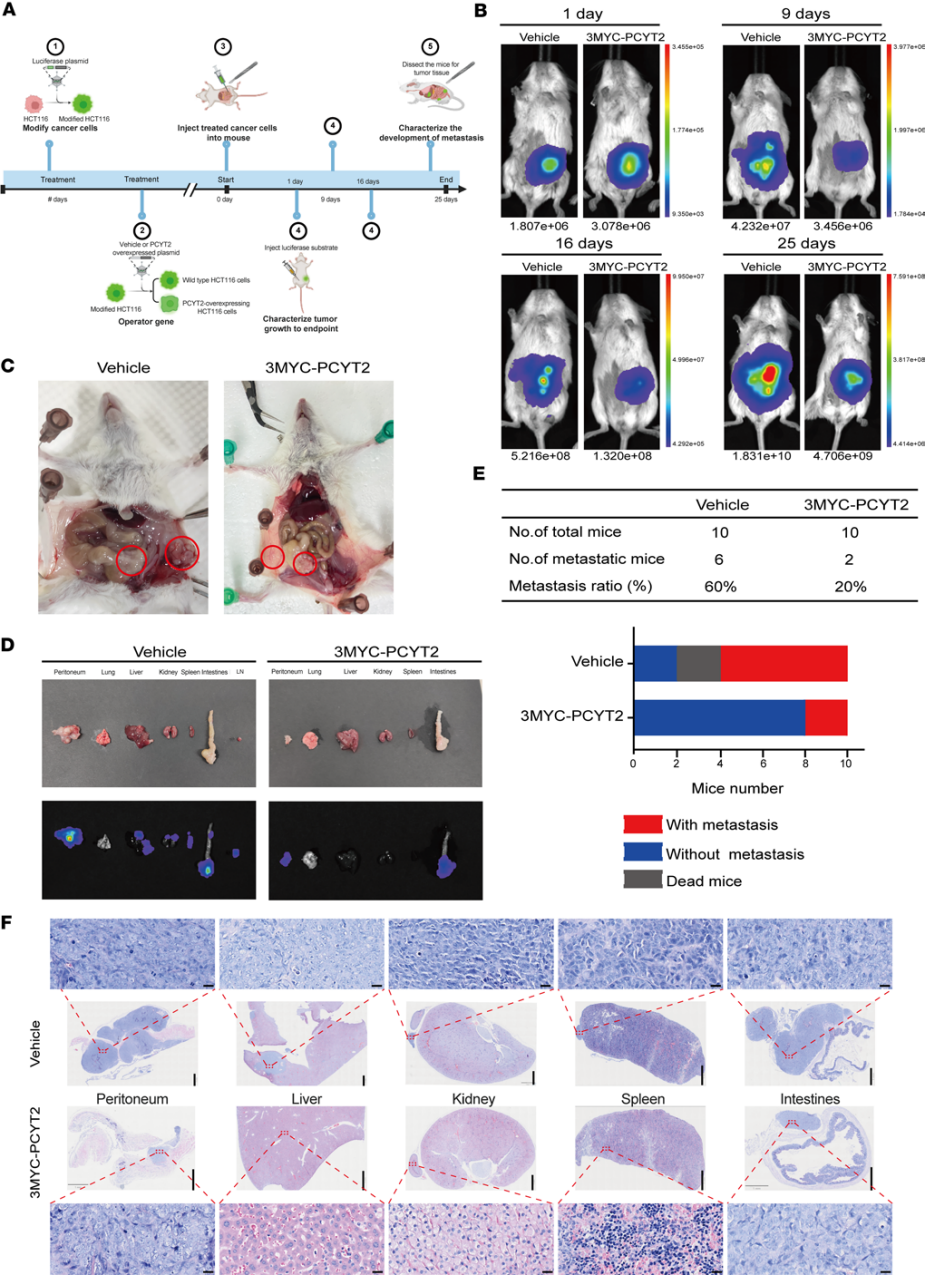
Overexpression of PCYT2 inhibits metastasis of colorectal cancer cells in mice. (**A**) Schematic diagram of orthotopic mouse model of CRC. (**B**) Representative images of fluorescence in orthotopic mouse after injection of CRC cells each week. (**C**) Representative images of abdominal tumors in mice after 30 days of dissection. (**D**) Representative images of fluorescence in mouse organs. LN, lymph node. (**E**) The statistics of tumor metastasis rate. (**F**) Representative images of H&E. The scale bar of the enlarged image is 20 μm, and the scale bar of the full image is 1 mm.

To investigate the effect of PCYT2 on in vivo metastasis of CRC cells, we established an orthotopic mouse model by intracecal injection of PCYT2-overexpression and control (vehicle) luciferase-labeled metastatic HCT116 cells (*n* = 10 per group) (Figure 3A). The progression and distribution of tumors were continuously evaluated by weekly injection of fluorescein substrates using bioluminescence imaging. We found that the fluorescence intensity of the PCYT2-overexpression group mice was weaker than that of the control group mice, indicating a decelerated growth rate of tumor cells in PCYT2-overexpression group mice (Figure 3B). After the fluorescence intensity of in vivo imaging reached its maximum, we dissected all mice to observe tumor growth. Our findings reveal that 60% of the mice in the control group exhibited metastases to distant sites, including the peritoneum, liver, kidney, and spleen, whereas only 20% of the mice from the PCYT2-overexpression group showed peritoneal metastasis (Figure 3, C–E). The pathological H&E staining also demonstrated the presence of tumor cells in the cecum, peritoneum, liver, kidney, and spleen tissues of the control group mice, while tumor cells were only observed in the cecum and peritoneum of PCYT2-overexpression group mice (Figure 3F).

The liver is the most common site for distant metastasis of CRC, followed by the lung as the second most frequent target organ for metastasis of CRC (31, 32). To determine whether PCYT2 also has an inhibitory effect on the metastasis of CRC to the lungs, we also established mouse models by injecting PCYT2-overexpression and control (vehicle) luciferase-labeled metastatic HCT116 cells into BALB/c nude mice (*n* = 7 per group) (Supplemental Figure 4A). Tumor progression and distribution were evaluated by serial bioluminescence imaging after the injection of the luciferin substrate. Overexpression of PCYT2 significantly reduced the fluorescence in the lungs compared with the vehicle group (Supplemental Figure 4, B–D). Mice injected with PCYT2-overexpression HCT116 cells had a lower rate of metastasis (2 of 7 mice) than those injected with vehicle control cells (6 of 7 mice) (Supplemental Figure 4, E and F). Pathological changes in ectopic foci tissues were detected using H&E and IHC staining. Mice injected with PCYT2-overexpression HCT116 cells showed a significant decrease in the number of metastatic tumor nodules and nodule size on the surface of the lungs compared with mice injected with vehicle control cells (Supplemental Figure 4G). Meanwhile, the IHC results show that PCYT2 was significantly overexpressed in cancer nodules (Supplemental Figure 4H). Collectively, these results suggest that PCYT2 inhibits the metastasis of CRC in vivo.

### PCYT2 increases YAP1 phosphorylation and relocates YAP1 to cytoplasm for degradation.

**Figure 4:**
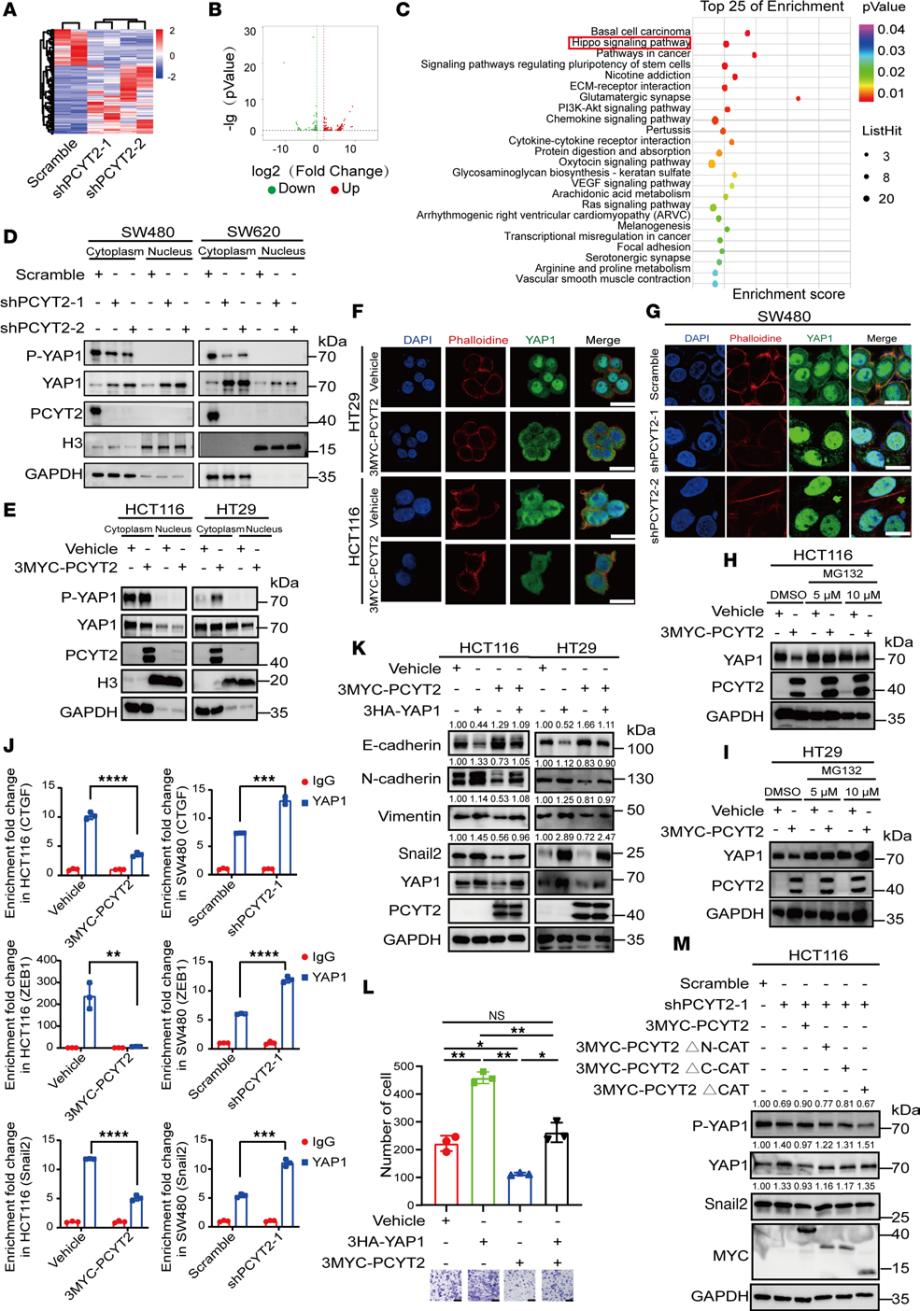
PCYT2 affects the phosphorylation levels of YAP1 and changes the proportion of YAP1 in the nucleus. (**A**) Heatmap showing upregulated and downregulated genes after PCYT2 knockdown (*P* < 0.05, [FC Log2] > 1). (**B**) Volcanic maps of upregulated and downregulated genes with PCYT2 knockdown (*P* < 0.05, [FC Log2] > 1). Red represents significantly upregulated genes, and green represents significantly downregulated genes. (**C**) Gene Ontology (GO) terms and KEGG pathways related to PCYT2 knockdown (*P* < 0.05, [FC Log2] > 1). (**D** and **E**) Detection of the YAP1 and p-YAP protein expression levels with PCYT2 knockdown (**D**) or PCYT2 overexpression (**E**) CRC cells in cytoplasm and nucleus, respectively, by Western blot. (**F** and **G**) Immunofluorescence analysis for the nuclear proportion of YAP1 in HT29, HCT116, and SW480 cells after PCYT2 overexpression (**F**) or knockdown (**G**). Different colors represent different antibodies or dyes: DAPI (blue), YAP1 (green), and phalloidine (red). Scale bar: 20 μm. (**H** and **I**) Detection of the expression changes of YAP1 in PCYT2-overexpression HCT116 (**H**) and HT29 (**I**) cells treated with MG132 by Western blot. (**J**) The enrichment of YAP1 in CTGF, ZEB1, and Snail2 promoters detected by ChIP assay. (**K**) Detection of epithelial and mesenchymal markers in CRC cell ectopic expression of vehicle, 3MYC-PCYT2, 3HA-YAP1, and 3MYC-PCYT2/3HA-YAP1 by Western blot. (**L**) Transwell assay was performed to detect cell migration capacity in HCT116 cell ectopic expression of vehicle, 3MYC-PCYT2, 3HA-YAP1, and 3MYC-PCYT2/3HA-YAP1. Statistical chart and representative images. Scale bar: 100 μm. (**M**) Detection of YAP1, p-YAP1, and Snail2 protein level in PCYT2- knockdown HCT116 cell ectopic expression of ectopic expression of WT PCYT2 or deletion of the cytidine transferase catalytic domain of PCYT2. Data represent the mean ± SD (*n* = 3). Statistical significance was assessed with 2-tailed unpaired Student’s *t* test (**J**) or 1-way ANOVA with Tukey’s multiple-comparison test (**L**). *P < 0.05, **P < 0.01, ***P < 0.001, ****P < 0.0001.

To investigate the underlying mechanisms by which PCYT2 suppresses metastasis, we first performed RNA-Seq in SW480 cells with PCYT2 knockdown (Figure 4A) and identified 224 differentially expressed genes, including 136 upregulated and 88 downregulated genes with the threshold of (log_2_[fold-change]) > 1 and *P* < 0.01 (Figure 4B) (Supplemental Table 1). Pathway enrichment analysis of the differentially expressed genes was carried out using the Kyoto Encyclopedia of Genes and Genomes (KEGG) public database (http://www.genome.jp/kegg/), suggesting an enrichment for several signaling pathways, including the Hippo and PI3K/AKT pathways (Figure 4C). Other biological processes (BPs), such as extracellular matrix disassembly and cell adhesion, were also enriched (Figure 4C), indicating that PCYT2 is possibly involved in the regulation of CRC cell migration. Among the enriched pathways identified, we particularly focused on the Hippo signaling pathway. The role of the Hippo pathway in EMT has been gradually elucidated (33). The mammalian pathway consists of core kinases (MST1/2 and LATS1/2), adaptor proteins (SAV1 for MST1/2 and MOB1 for LATS1/2), downstream effectors (YAP and its analog TAZ), and nuclear transcription factors (TEAD1-4) (34). The Hippo signaling components LATS1/2, each encoding a tumor-suppressive serine or threonine-protein kinase, phosphorylate their effectors TAZ/YAP, causing YAP1 and TAZ’s cytoplasmic retention and destabilization. This retention in the cytoplasm inhibits TAZ/YAP activity by preventing their translocation to the nucleus, resulting in the transcriptional inhibition of downstream targets such as oncogene c-MYC and mesenchymal genes ZEB1/SNAIL2, which drive metastasis (34–38). During tumor development, YAP1 phosphorylation is inhibited, and its downstream regulators YAP1 and TAZ enter the nucleus, where they increase the transcriptional activation of genes involved in cell proliferation and metastasis (36, 39).

To investigate the effect of PCYT2 on the Hippo pathway, the expression of its core effectors YAP1/TAZ and the phosphorylation status of YAP1 at Ser127 were determined in PCYT2-knockdown CRC cells. Western blot indicated an increase in YAP1 expression and a decrease in the phosphorylation of YAP1 compared with control, while TAZ expression had a little change (Supplemental Figure 5A). In contrast, a decrease in total YAP1 expression and an increase in the phosphorylation of YAP1 were observed in PCYT2-overexpression CRC cells (Supplemental Figure 5B). YAP1 acts as the core component of the Hippo pathway, and nuclear YAP localization indicates Hippo pathway inactivation (34). Therefore, we detected the amount of YAP1 in the nucleus and cytoplasm by nucleocytoplasmic separation in CRC cells with PCYT2 either knocked-down or overexpressed. We observed the remarkable increase of YAP1 in the nucleus and reduction of phosphorylated YAP1 (p-YAP1) in the cytoplasm of PCYT2-knockdown CRC cells (Figure 4D). Consistently, YAP1 was decreased significantly in the nucleus, while p-YAP1 was increased significantly in the cytoplasm of PCYT2-overexpressed CRC cells (Figure 4E). In line with the results of nucleocytoplasmic separation, the immunofluorescence results also clearly indicated that PCYT2 overexpression led to a marked reduction in YAP1 in the nucleus and to an obvious increase in the cytoplasm (Figure 4F). Meanwhile, YAP1 was translocated into the nucleus after PCYT2 downregulation (Figure 4G). These data indicate the critical role of PCYT2 in the regulation of YAP1 phosphorylation and nuclear localization.

p-YAP1 is ubiquitinated and degraded via the ubiquitin-proteasome pathway (40). Because PCYT2 affected p-YAP1 levels (Figure 4, D and E, and Supplemental Figure 5, A and B), we speculated that changes in PCYT2 expression would eventually lead to altered YAP1 ubiquitin levels. Thus, we investigated the ubiquitination level of YAP1 and found that it was indeed reduced in PCYT2-knockdown CRC cells (Supplemental Figure 5C), while PCYT2-overexpression HCT116 cells tended to increase ubiquitin levels (Supplemental Figure 5D). Simultaneously, we treated PCYT2-overexpression HCT116 and HT29 cells with proteasome inhibitor MG132, and we found that MG132 inhibited the degradation of YAP1 in the PCYT2-overexpression group (Figure 4, H and I); however, it had no effect on PCYT2-knockdown SW480 cells (Supplemental Figure 5E). Since YAP1 phosphorylation represses its transcriptional activity by promoting YAP1 accumulation in the cytoplasm (34, 35), we evaluated the effect of PCYT2 on YAP1’s transcriptional ability in regulating the EMT transcription program. We observed a significant reduction in YAP1 bound to proximal promoter regions of CTGF, ZEB1, and SNAIL2 in HCT116 cells with ectopic PCYT2 expression (Figure 4J). In contrast, loss of PCYT2 markedly increased the enrichment of YAP1 in proximal promoter regions of these genes (Figure 4J). In addition, we examined the effect of YAP1 on EMT and migration and found that YAP1 could promote the migration and EMT of CRC (Supplemental Figure 6, A–E). Importantly, expression of YAP1 in HCT116 cells counteracted the capacity of PCYT2 to repress cell migration (Figure 4, K and L), supporting the idea that YAP1 functions as a target of PCYT2. Simultaneously, we observed that overexpression of PCYT2 effectively reinstated the expression of YAP1, p-YAP1, and the mesenchymal marker Snail2 in PCYT2-knockdown HCT116 cells (Figure 4M). However, overexpression of mutants lacking the cytidylytransferase domain of PCYT2 failed to elicit such restorative effects in PCYT2-knockdown HCT116 cells (Figure 4M). Therefore, these data suggest that YAP1 is one of potential targets repressed by PCYT2 in manipulating the EMT transcription program.

### PCYT2 regulates EMT in CRC by inducing YAP1 relocation.

Lipids are able to transduce signals in a variety of physiological processes through specific lipid-protein interactions (18, 41). Therefore, we hypothesized that PCYT2 functions in the regulation of EMT by affecting PE synthesis and function in lipid-protein interactions. PEBP1 (also known as Raf1 kinase inhibitory protein [RKIP]) belongs to the family of PE-binding proteins (PEBPs) and has been implicated in various diseases, including cancer, Alzheimer’s disease, diabetes, and retinopathies (42–46). In particular, PEBP1 is reported to suppress cell motility and metastasis development and is downregulated in prostate cancer (45). Previous studies have shown that PE binding to Pebp1 disrupts the association of Pebp1 with Raf-1 and MAPK kinase 1 (MEK1) while enhancing its interaction with kinases upstream of NF-κB (18, 47, 48). However, the specific signaling pathways regulated by PE-PEBP1 interaction in CRC metastasis remain elusive.

**Figure 5:**
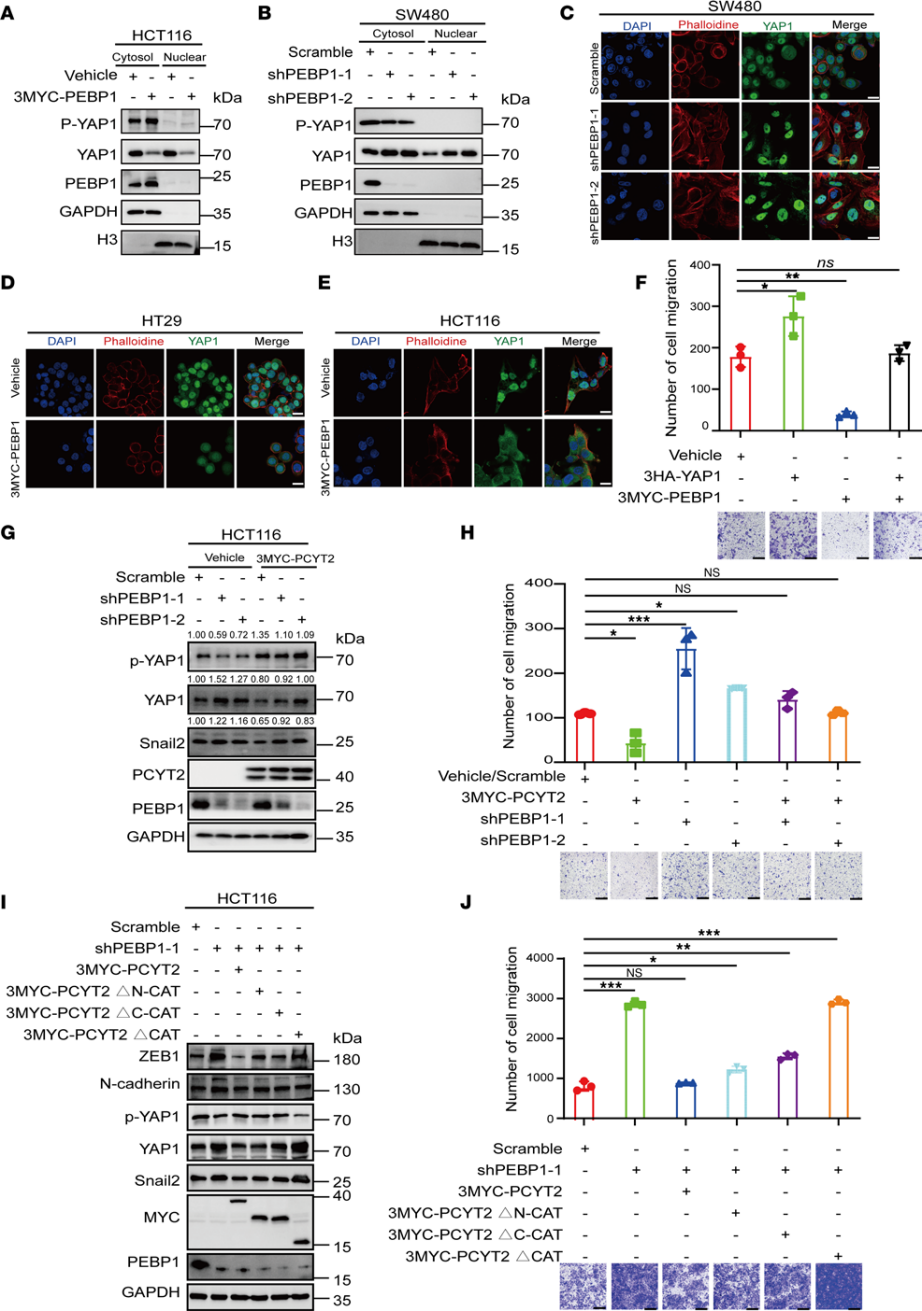
PCYT2 regulates EMT in CRC via YAP1 translocation induced by PEBP1. (**A** and **B**) Western blot results show the expression of YAP1 and phosphorylated YAP1 (p-YAP1) in the nuclear and cytoplasmic of PEBP1-overexpression HCT116 (**A**) and PEBP1-knockdown (**B**) SW480 cells. (**C**–**E**) Detection of the expression changes in the nuclear proportion of YAP1 in CRC cells with PEBP1-knockdown (**C**) or PEBP1-overexpression (**D** and **E**) by immunofluorescence assays. Different colors represent different antibodies or dyes: DAPI (blue), YAP1 (green), and phalloidine (red). Scale bar: 20 μm. (**F**) Detection of the cell migration capacity in ectopic expression of vehicle, 3MYC-PEBP1, 3HA-YAP1, and 3MYC-PEBP1/3HA-YAP1 HCT116 cells by Transwell assay. Statistical chart and representative images. Scale bar: 100 μm. (**G**) Detection of the expression of Snail2, YAP1, and p-YAP1 in shPEBP1, 3MYC-PCYT2, and shPEBP1/3MYC-PCYT2 HCT116 cells by Western blot. (**H**) Transwell assays in HCT116 cells described in **G**. Scale bar: 100 μm. Statistical chart and representative images. (**I**) Detection of the expression of YAP1, p-YAP1, and epithelial and mesenchymal markers in PEBP1-knockdown HCT116 cells with PCYT2 or kinase inactive PCYT2 mutant. (**J**) Representative images and of Transwell assays for shPEBP1 cells with ectopic expression of WT PCYT2 or deletion of the cytidine transferase catalytic domain of PCYT2. Statistical chart and representative images. Scale bar: 100 μm. Data represent the mean ± SD (*n* = 3). *P < 0.05, **P < 0.01, ***P < 0.001 by 1-way ANOVA with Tukey’s multiple-comparison test.

Therefore, we wondered whether PCYT2 functions in EMT regulation by affecting the binding of PE with PEBP1. We initially investigated whether the upregulation of PEBP1 leads to the relocation of YAP1 to the nucleus. In concert with the finding of PCYT2-mediated YAP1 relocation, overexpression of PEBP1 in CRC cells led to a significant increase in p-YAP1 accumulation in the cytoplasm and a decrease in YAP1 accumulation in the nucleus (Figure 5A), suggesting that YAP1 may undergo ubiquitination and protein degradation. Conversely, downregulation of PEBP1 resulted in markedly decreased expression of p-YAP1 and increased accumulation of YAP1 in the nucleus (Figure 5B). Meanwhile, the results of immunofluorescence analysis were consistent with those of Western blot, revealing that knockdown of PEBP1 in SW480 cells enhanced the accumulation of YAP1 in the nucleus and that overexpression of PEBP1 reduced the aggregation of YAP1 in the nucleus. (Figure 5, C–E). Additionally, we observed the inhibitory role of PEBP1 in CRC cell migration (Supplemental Figure 7, A–G). In addition, ectopic expression of YAP1 in HCT116 cells counteracted the repressive effect of PEBP1 on cell migration (Figure 5F). Collectively, these results imply that PEBP1 inhibits CRC migration by altering YAP1 distribution in the nucleus, similar to the effect of PCYT2.

To further clarify the role of the PCYT2/PEBP1 axis on YAP1 relocation, we knocked down PEBP1 in PCYT2-overexpression HCT116 cells. We observed that PEBP1 deficiency compensates the loss of YAP1 caused by PCYT2 overexpression and that YAP1 supplementation partially abolished PCYT2 inhibitory role in CRC migration (Figure 5, G and H). The effects of overexpressing PCYT2 or its cytidyltransferase deletion mutant on the migration phenotype of PEBP1-knockdown HCT116 cells were simultaneously examined. We found that overexpression of full-length PCYT2 restored the expression of YAP1, p-YAP1, and migration ability in PEBP1-knockdown cells (Figure 5, I and J). However, only partial restoration of the expression of YAP1, p-YAP1, and migration ability was observed in PEBP1-knockdown HCT116 cells when they were transfected with mutants lacking either the C-terminal transferase domain or the N-terminal transferase domain alone (Figure 5, I and J). Conversely, overexpression of the deletion of both N-terminal and C-terminal cytidylytransferase domains of PCYT2 did not affect the expression of YAP1, p-YAP1, or migration ability changes in PEBP1-knockdown HCT116 cells (Figure 5, I and J). Therefore, these data suggest that PCYT2 regulates cell migration by affecting the nuclear distribution of YAP1 through PEBP1.

### PEBP1 binds to PPP2R1A phosphatase but not YAP1.

**Figure 6:**
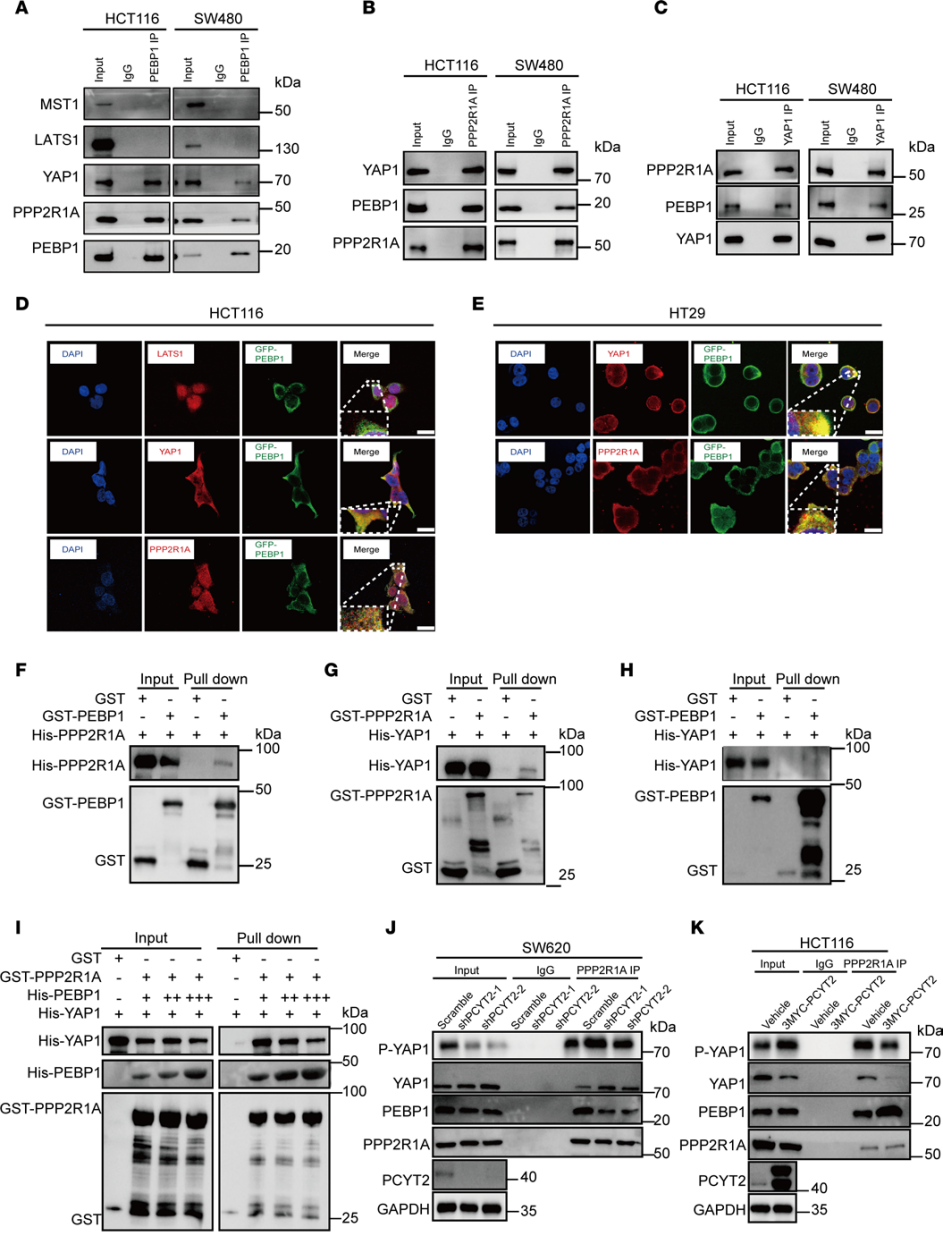
PPP2R1A interacts with YAP1 and PEBP1. (**A**) Detection of the interaction between PEBP1 and MST1/LATS1/YAP1/PPP2R1A in HCT116 and SW480 cells by coimmunoprecipitation. (**B**) Detection of the interaction between PPP2R1A and PEBP1/YAP1 in HCT116 and SW480 cells by coimmunoprecipitation. (**C**) Detection of the interaction between YAP1 and PEBP1/PPP2R1A in HCT116 and SW480 cells by coimmunoprecipitation. (**D**) The colocalization of LATS1/YAP1/PPP2R1A and GFP-PEBP1 in HCT116 cells was detected by immunofluorescence. Scale bar: 20 μm. (**E**) Representative images of the colocalization of YAP1/PPP2R1A and GFP-PEBP1 in HT29 cells. Scale bar: 20 μm. (**F**) The validation of the interactions between PEBP1 and PPP2R1A in vitro by GST pull-down assay. (**G**) The validation of the interactions between YAP1 and PPP2R1A in vitro by GST pull-down assay. (**H**) The validation of the interactions between PEBP1 and YAP1 in vitro by GST pull-down assay. (**I**) The validation of the interactions between PEBP1, PPP2R1A, and YAP1 in vitro by GST pull-down assay. (**J**) Detection of the interaction between PPP2R1A and PEBP1/YAP1 in PCYT2-knockdown SW620 cells by coimmunoprecipitation. (**K**) Detection of the interaction between PPP2R1A and YAP1/PEBP1 after PCYT2 overexpression in HCT116 cells overexpressing PCYT2 by coimmunoprecipitation.

Next, we explored the mechanism by which the PCYT2/PEBP1 axis affects YAP1 phosphorylation levels and nuclear translocation. Binding of PE to PEBP1 has been shown to disrupt the association between PEBP1 and certain kinases (18, 47). Thus, we speculated that PEBP1 may bind to the upstream kinases of YAP1 to affect the protein phosphorylation cascade in the Hippo pathway. To validate this hypothesis, we initially examined whether PEBP1 interacts with MST1 and LATS1, the upstream kinases of YAP1. However, we did not observe the coimmunoprecipitation (Co-IP) of MST1/LATS1 with PEBP1 in CRC cells (Figure 6A). This result prompted us to speculate the possibility that PEBP1 probably does not function in direct interaction with kinases involved in YAP1 phosphorylation. To further identify the regulatory network of YAP1, we employed affinity purification–mass spectrometry (MS) to uncover the proteins associated with PEBP1. Among 201 candidate proteins identified in GFP-PEBP1–overexpressing cells, PPP2R1A — a subunit of PP2A — attracted our attention (Supplemental Table 2) since PPP2R1A was listed as interacting with YAP1 in the BioGRID database (https://thebiogrid.org/). To confirm the interaction among PEBP1, PPP2R1A, and YAP1, we performed Co-IP using specific antibodies against each protein in CRC cells. We found that PEBP1 indeed interacts with PPP2R1A and YAP1 and vice versa (Figure 6, A–C). The colocalization of these proteins in the cytoplasm detected by immunofluorescence was also observed (Figure 6, D and E). To verify the authenticity of the interactions detected in vivo, we performed an in vitro GST pull-down assay. Indeed, the PEBP1-PPP2R1A and YAP1-PPP2R1A interactions were observed but not the PEBP1-YAP1 interaction (Figure 6, F–H), suggesting that PEBP1 likely regulates YAP1 function by interacting with PPP2R1A. Intriguingly, this mutual interaction between YAP1 and PPP2R1A was significantly reduced following the increase in PEBP1 protein level, implying that PEBP1 can compete with YAP1 to bind PPP2R1A, which in turn stabilizes the YAP1 protein (Figure 6I). This possibility is supported by the finding that the PP2A complex negatively regulates Hippo signaling by dephosphorylating YAP1 (49, 50). These results suggest that PEBP1 may affect the phosphorylation of YAP1 by binding to PPP2R1A. Therefore, we speculate that the change in PCYT2 expression could affect the interaction between PEBP1 and downstream proteins. To test this possibility, we performed Co-IP using a specific antibody against PPP2R1A to detect interactions among PPP2R1A, PEBP1, and YAP1. The results show that PCYT2 knockdown weakened the binding of PPP2R1A to PEBP1, whereas it induced the binding of YAP1 with PPP2R1A (Figure 6J). In contrast, overexpression of PCYT2 enhanced the binding of PPP2R1A to PEBP1, while it weakened the binding of YAP1 to PPP2R1A (Figure 6K). These findings indicate that PCYT2 regulates the phosphorylation of YAP1 by promoting the association of PEBP1 with PPP2R1A in order to impede the interaction of PPP2R1A with YAP1.

### PPP2R1A facilitates nuclear distribution of YAP1 and cell migration by reducing YAP1 phosphorylation.

**Figure 7:**
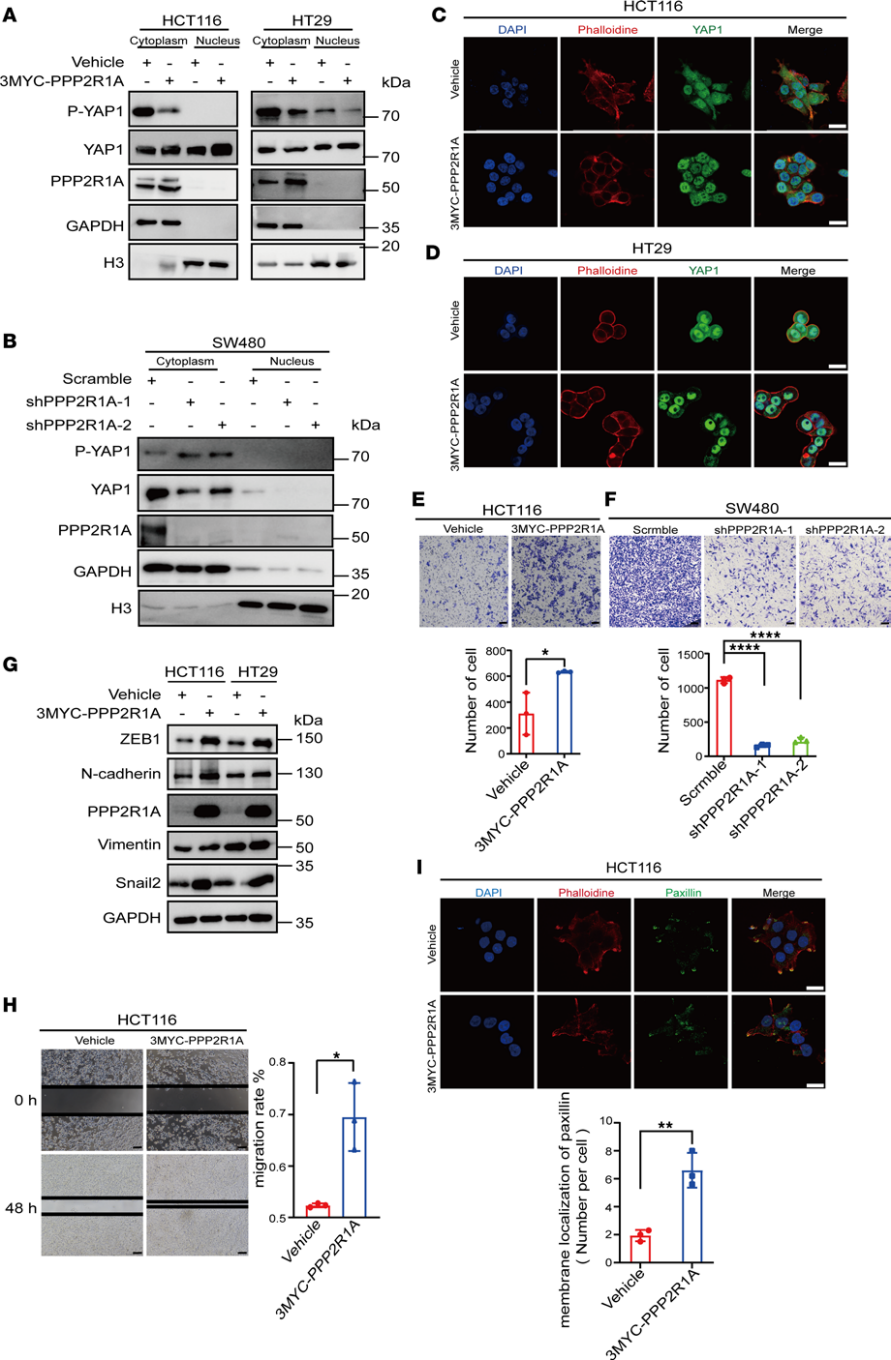
PPP2R1A dephosphorylates YAP1 and leads to changes in the proportion of YAP1 entering the nucleus. (**A**) Detection of the YAP1/p-YAP1 expression levels in the nucleoprotein and in cytoplasmic with ectopic expression of vehicle and 3MYC-PPP2R1A HCT116 and HT29 cells by nucleoplasmic separation and Western blot. (**B**) Detection of the YAP1/p-YAP1 expression in the nucleoprotein and cytoplasmic at SW480 cells infected with control and shPPP2R1A by nucleoplasmic separation and Western blot. (**C** and **D**) Detection of the changes in the nuclear proportion of YAP1 in CRC cells with PPP2R1A overexpression (**C**) or knockdown (**D**) by immunofluorescence staining. Different colors represent different antibodies or dyes: DAPI (blue), YAP1 (green), and phalloidine (red). Scale bar: 20 μm. (**E** and **F**) Transwell assays of HCT116 (**E**) and SW480 (**F**) cells demonstrate that cells with higher levels of PPP2R1A had a higher migratory ability than cells with lower PPP2R1A levels. Scale bar: 100 μm. Representative images and statistical chart. (**G**) Detection of the epithelial and mesenchymal markers with ectopic expression of vehicle and 3MYC-PPP2R1A HCT116 and HT29 cells by Western blot. (**H**) Representative images of scratch assays in PPP2R1A-overexpression HCT116 cells and control group. The graph shows the area of a wound evaluated with ImageJ. Scale bar: 500 μm. Representative images and quantification. (**I**) Immunofluorescence assays were performed to determine the membrane localization of paxillin in HCT116 cells with PPP2R1A overexpression. Scale bar: 20 μm. Representative images and statistical chart. Data represent the mean ± SD (*n* = 3). Statistical significance was assessed with 2-tailed unpaired Student’s *t* test (**E**, **H**, and **I**) or 1-way ANOVA with Tukey’s multiple-comparison test (**F**). *P < 0. 05, **P < 0.01, ****P < 0.0001.

Since PPP2R1A is functionally annotated as a subunit of phosphatase PP2A, we hypothesize that PPP2R1A could change YAP1 phosphorylation levels in CRC cells. To investigate this hypothesis, we detected YAP1 phosphorylation levels in CRC cells ectopically expressing PPP2R1A. PPP2R1A overexpression remarkably reduced the p-YAP1 in the cytoplasm, while it increased the accumulation of YAP1 in the nucleus (Figure 7A). Conversely, PPP2R1A knockdown greatly increased YAP1 phosphorylation, whereas total YAP1 expression was decreased (Figure 7B). The immunofluorescence assays also showed an enhanced fluorescence signal of YAP1 in the nucleus after the overexpression of PPP2R1A, whereas PPP2R1A knockdown weakened the signal (Figure 7, C and D). Additionally, we found that knocking down PPP2R1A inhibited CRC cell migration, while PPP2R1A overexpression had the opposite effect (Figure 7, E–I). These results indicate that PPP2R1A probably dephosphorylates YAP1 to promote YAP1 nuclear distribution and cell migration.

## Discussion

The reprogramming of metabolism is a hallmark of cancer, and recent discoveries suggest that alterations in lipid metabolism play an important role in tumor development. A better understanding of these metabolites will be instrumental in the design of targeted agents for pharmacological interventions in tumorigenesis. The regulation of lipid metabolism is extremely complicate, and metabolic processes commonly involve multiple metabolic enzymes. Understanding these enzymes and pathways has provided potentially new avenues for the development of therapeutic strategies targeting tumor-related diseases (7). PE is an essential component of biological membranes that plays a key role in cell functions (11, 12). PCYT2 is a key enzyme in one of the 2 major pathways of PE biosynthesis (51). In the current study, we showed that PCYT2 expression was downregulated in tumor tissues compared with adjacent tissues, and patients with CRC had a better prognosis when they showed higher PCYT2 expression. Furthermore, we identified that PCYT2 noticeably inhibited the metastasis of CRC cells. These results suggest the involvement of PCYT2 in negative regulation on CRC metastasis.

PEBP1, also known as RKIP, is considered a potential tumor suppressor gene and plays a critical role in multiple important signaling pathways (47, 52, 53). Here, we found that PEBP1 inhibited CRC metastasis, which further supports the notion about PEBP1 is critical for signal transduction. In previous reports, PEBP1 is also considered as a scaffold protein that inhibits protein kinase cascades such as the ERK pathway and NF-κB pathway. However, our results do not indicate that PEBP1 functioned as a kinase inhibitor in CRC, while it manipulated YAP1 phosphorylation by directly binding with PPP2R1A, a subunit of phosphatase PP2A. Previous studies reveal that PCYT2 affects the interaction between PEBP1 and downstream kinases in 2 ways; PCYT2 either disrupts the association between PEBP1 and certain kinases, including RAF1 and MAPK kinase 1 (MEK1), or enhances the interaction of PEBP1 with kinases in NF-κB pathway (18). In this study, we reveal a potentially new function of PCYT2: PCYT2 enhanced the binding of PEBP1 to phosphatase PPP2R1A to regulate YAP1 phosphorylation.

Reversible protein phosphorylation is a widespread modification that is involved in multiple cellular processes. While numerous studies have investigated the different kinases and substrates in the Hippo pathway, few have focused on dephosphorylation and phosphatase. Among the 150 known protein phosphatases, 4 have been identified as regulators of the Hippo/YAP pathway, including PP2AC, which targets MST kinases (49, 50, 54); PP1, which selectively dephosphorylates TAZ (55) and LATS1 (56); Pez/PTPN14, which interacts with TAZ and LATS1 (57); PPM1A, which binds to YAP/TAZ in both the cytoplasm and nucleus (58); and phosphatase PPP1R12A, which is regulated by phosphatidylserine (59). Based on our data, we showed that overexpression of PCYT2 or PEBP1 led to change in the phosphorylation level of YAP1. To understand the underlying mechanism, we screened the interacting protein of PEBP1 through liquid chromatography/MS (LC/MS) and identified PPP2R1A phosphatase as a potential candidate. Further evidence shows the direct interaction between PPP2R1A and PEBP1, and PPP2R1A indeed regulates YAP1 phosphorylation. Though PP2AC induces the binding and dephosphorylation of MST1/2 and MAP4Ks (50, 60), the finding by Zhou et al. reveal that the reducing effect of PPP2R1A (PP2AA) on the transcriptional activity resulting from changing the phosphorylation of Hippo pathway is stronger than PP2AC (58). Therefore, we suspect that the phosphorylation of YAP1 is manipulated by PPP2R1A/PP2AA rather than PP2AC. Indeed, our finding supports this speculation by which PPP2R1A/PP2AA directly binds to YAP1 to remove YAP1 phosphorylation rather than via the phosphorylation of MST1/2. PPP2R1A directly targets on YAP1 (a core factor of the Hippo pathway), and its regulation on YAP1 phosphorylation is stronger than PP2AC (while PP2AC interacts with the upstream kinases MST1/2 and MAP4Ks), providing an explanation for the general phenomenon (59). Interestingly, PCYT2 promotes the interaction of PPP2R1A and PEBP1 and simultaneously reduces the binding of PPP2R1A and YAP1, leading to the enhancement of YAP1 phosphorylation, which in turn inhibits CRC cell migration and invasion. In line with this discovery, elevated PPP2R1A expression promotes CRC cell migration and invasion.

In summary, we uncovered an underlying mechanism showing that PCYT2 attenuated the expression of key EMT regulators by manipulating YAP1 phosphorylation in a PEBP1-dependent manner. Specifically, the downregulation of PCYT2 in cancer cells not only decreased PE level but also attenuated the interaction between PEBP1 and PPP2R1A. As a result, the interaction between YAP1 and PPP2R1A was enhanced, leading to dephosphorylation of YAP1 and nucleus relocation to upregulate the transcription of EMT key regulators such as ZEB1 and Snail2, eventually facilitating metastasis. Collectively, these findings reveal that PCYT2 inhibited the metastasis of CRC through the PCYT2/PEBP1/YAP1 axis, supporting the significance of metabolic pathways in regulation on cancer metastasis, and they shed light on PCYT2 as a potential therapeutic target for CRC metastasis.

## Methods

### Sex as a biological variable.

This study involved male and female individual clinical specimens. Female mice were used for all animal investigations. Sex was not considered as a biological variable.

### Clinical samples.

Tumor and surrounding nontumor tissue samples were obtained from patients with CRC at West China Hospital, Sichuan University. All enrolled patients provided informed consent, and the study was approved by the Ethics Committee of Clinical Research of the West China Hospital of Sichuan University. Tissue specimens were approximately 0.5 cm^3^ in size, and the surrounding nontumor tissue was taken from the normal site within 1–3 cm from the tumor site. Specimens were cleaned with normal saline immediately after removal and stored in liquid nitrogen within 30 minutes.

### Lung metastasis mouse model of CRC.

Six-week-old female BALB/c nude mice were purchased from Beijing Huafukang Biotechnology Co. Ltd. The mice were injected with 1.5 × 10^6^ of HCT116 cells containing the luciferase reporter gene and PCYT2 overexpression or vector control via tail vein. After injection, fluorescein substrate was injected i.p. weekly to allow us to check lung fluorescence and observe lung tumor metastasis. Tissue samples were fixed in 4% formalin and embedded to obtain 3–5 μm–thick continuous sections for H&E staining.

### Orthotopic mouse model of CRC.

Orthotopic mouse model of CRC was established according to ref. 61. Six-week-old female NCG mice were purchased from GemPharmatech Co. Ltd. The mice were injected with 1 × 10^6^ of HCT116 cells containing the luciferase reporter gene and PCYT2 overexpression or vector control via intracecal injection. After injection, fluorescein substrate was injected i.p. weekly to allow us to check intracecal fluorescence and observe tumor metastasis. Tissue samples were fixed in 4% formalin and embedded to obtain 3–5 μm–thick continuous sections for H&E staining.

### Cell culture.

Human normal intestinal epithelial cells (NCM460) and human CRC cells (HCT116, HT29, SW480, SW620, and LOVO) were purchased from the cell bank of the Chinese Academy of Science (Shanghai, China). All cell lines were tested for mycoplasma contamination, identified by short tandem repeat analysis. Cells were cultured in high-glucose DMEM containing 10% FBS and 1% penicillin-streptomycin double antibiotic solution at 37°C in atmosphere of 5% CO_2_.

### IHC assay.

Tissue samples were fixed in 4% formalin and embedded in paraffin to obtain 3–5 μm–thick serial sections for and IHC staining. The sections were dewaxed and deparaffinized in xylene and rehydrated in graded alcohol solutions. After antigen retrieval by heating the sections for 30 minutes in sodium citrate buffer, the slides were stained with specific antibodies (Supplemental Table 3). The sections were then counterstained with hematoxylin, followed by dehydration and mounting. The immunoreactivity score was separately for 2 variables, first according to the percentage of positive cells with staining (< 25% = 1, 26%–50% = 2, 51%–75% = 3, and > 75% = 4) and then according to the overall intensity of the immunoreactivity of cells that stained positive (no staining = 0, light staining = 1, moderate staining = 2, and marked staining = 3).

### Western blot.

Western blot was performed as previously described (62). Briefly, total proteins were extracted form cells using RIPA buffer containing protease and phosphatase inhibitors cocktail (Bimake, B14001 and B15001). The protein lysates were subjected to separate on 12.5% or 10% SDS-PAGE and subsequently transferred onto PVDF membranes. After being blocked in 5% milk, membranes were incubated with the primary antibody (Supplemental Table 3) at a diluted concentration based on the manufacturer’s protocol. The immunoblot band were detected by enhanced chemiluminescence detection kit (MilliporeSigma, WBKLS0500).

### Construction of shRNAs and plasmids.

shRNA oligos of PCYT2/PEBP1/YAP1 (Supplemental Table 4) were cloned into pLKO.1-TRC and transfected into HEK293T cells, along with the packing plasmids psPAX2 and pMD2G to package the PCYT2/PEBP1/YAP1 knockdown virus. CRC cells were infected with virus for 48 hours and then selected with media containing 2.5 μg/mL puromycin until the density of positively infected cells reached 80%–90% confluence. Human PCYT2/PEBP1/YAP1 genes were amplified by PCR and cloned into plenti–CMV-3MYC, plenti–CMV-GFP, or plenti–CMV-3HA. The recombinant plasmid was transfected into CRC cell lines by using polyethylenimine (PEI) or plentivirus. All plasmid constructs were confirmed by DNA-Seq before use, and the expression efficiency was validated by reverse transcription PCR (RT-PCR) and/or Western blot.

### Migration assay.

The migration and invasion abilities were analyzed using a Boyden chamber (Corning Inc.) migration assay. After starvation incubation without FBS for 24 hours, proper CRC cells (30,000 per well in HCT116 and 300,000 per well in SW480) were seeded per chamber in 24-well plates. Then, 800 μL of medium containing 10% FBS was added to the lower chamber, and 200 μL of medium containing 2% FBS was added to the upper chamber. Migratory cells on the lower surface of the filter were fixed in 4% formaldehyde after 24–48 hours of incubation and stained with crystal violet solution. Cells were photographed and counted using a light microscope. The cell scratch area and the number of cells that penetrated the Transwell chamber membrane were analyzed using ImageJ.

### Colony formation assay.

CRC cells with PCYT2 knockdown, overexpression, or vector control (3,000 per well) were seeded in 6-well plates and incubated at 37°C in a humidified incubator for 2 weeks. Cell colonies were fixed with formalin and stained with crystal violet. The total number of colonies was calculated at the end of each experiment.

### RNA isolation and quantitative PCR.

The FOREGENE Cell Total RNA Isolation Kit was used to extract total RNA from cells. NanoDrop 2000c (Thermo Fisher Scientific) was used to determine RNA concentration and quality. An RT kit (PrimeScript RT Reagent Kit; Takara) was used to transcribe RNA into cDNA, and quantitative PCR was performed using SYBR Green (NovoStart SYBR qPCR SuperMix Plus). Fold-change analysis was performed used the method (63). ACTIN was used as the normalized control. Primers used for quantitative real-time PCR are listed in Supplemental Table 4.

### RNA-Seq.

CRC cells were lysed in TRIzol Reagent (Invitrogen), and total RNA was extracted according to the manufacturer’s instructions. Library preparation and RNA-Seq were performed by Novogene Bioinformatics Technology Co. Differential expression analysis of the PCYT2 knockdown or vector control was performed using the DESeq R package (1.10.1). Benjamini and Hochberg’s approach was used for *P* value adjustment to control the FDR. An adjusted *P* < 0.05 indicated that the gene was differentially expressed.

### Gene ontology and pathway analysis.

Gene ontology (GO) and pathway analysis were conducted to better understand the roles of differentially expressed genes. In this process, 3 independent categories, including BP, cellular component, and molecular function, were derived from the GO Consortium website (http://www.geneontology.org). The Database for Annotation, Visualization, and Integrated Discovery (https://davidbioinformatics.nih.gov/) was used to assess changes in the upregulated and downregulated genes. The biological pathways with markedly enriched genes that were differentially expressed were analyzed using the KEGG database.

### Immunofluorescence staining.

For immunofluorescence assays, cells were seeded at a density of 1 × 10^5^ cells/well on a coverslip in 24-well plates. After transfection, the coverslips were fixed in cold PBS buffer and washed 3 times. Then, the coverslips were fixed with 4% formaldehyde, and the cells were permeabilized with 0.5% Triton X-100 (Sigma-Aldrich) for 10 minutes and washed thoroughly with PBST buffer. Lastly, cells were blocked with goat serum for 40 minutes at room temperature, followed by incubation with primary antibodies overnight at 4°C (Supplemental Table 3). The cells were washed 5 times with PBST buffer and then incubated with Alexa Fluor 488–tagged or Alexa Fluor 594–tagged secondary antibodies (Supplemental Table 3). DAPI (Beyotime Biotechnology) was used to stain DNA.

### Immunoprecipitation assay.

Immunoprecipitation was performed as previously described (64). Cells were lysed with lysis buffer (50 mM Tris-HCl [pH 7.5], 150 mM NaCl, 0.2% NP-40, 5% glycerol, 1% [v/v] Tween-20, and protease inhibitor cocktail; see *Western blot* for manufacturer). Cell lysates were centrifuged at 12,000*g* at 4°C for 15 minutes and incubated with antibodies (Supplemental Table 3) at 4°C overnight before incubation with protein A/G magnetic beads (MedChemExpress) for 3 hours. The beads were washed 3 times for 5 minutes with lysis buffer before being evaluated for immunoprecipitated proteins using Western blot.

### ChIP assay.

HCT116 and SW480 cells were fixed with 1% formaldehyde for 10 minutes and then quenched with 125 mM glycine. Cell extracts were prepared by mechanical sonication, and protein-DNA complexes were immunoprecipitated using anti-YAP1 antibodies (Cell Signaling Technology, 14074T). Immunoprecipitated DNA was analyzed using real-time PCR with specific primers for the CTGF/ZEB1/SNAIL2 promoter region (Supplemental Table 4).

### Purification of GST-tagged recombinant proteins.

PEBP1 and PPP2R1A were cloned into the pGEX-6P-1 vector and transformed into DE3 (BL21). The expression of GST-PEBP1/PPP2R1A was induced by IPTG (0.5 mM) at 16°C for 16 hours, and DE3 cells were suspended in 50 mL of PBS buffer (140 mM NaCl, 2.7 mM KCl_2_, 10 mM Na_2_HPO_4_, 1.8 mM KH_2_PO_4_, and 1 mM PMSF [pH 7.5]), sonicated, and centrifuged at 12,000*g* at 4°C for 20 minutes. All supernatants were added to a gravity column with 15 mL of elution buffer (50 mM Tris-HCl and 10 mM reducing glutathione). The supernatants were examined for the expression of the indicated proteins by SDS-PAGE and Coomassie blue staining.

### Purification of His-tagged recombinant proteins.

PEBP1/PPP2R1A/YAP1 was cloned into the pCold-pros2 vector and transformed into DE3 (BL21) before induction with 0.5 mM IPTG at 16°C for 16 hours. Bacteria were suspended in 50 mL of lysis buffer (0.5 mM Tris-HCl, 0.5M NaCl, 10 mM imidazole, 5% glycerin, 1% NP-40, 0.25% Tween-20, and 1 mM PMSF [pH 8]), sonicated, and centrifuged for 12,000*g* at 4°C for 20 minutes. Supernatant was combined with 15 mL of elution buffer (50 mM Tris-HCl, 0.5M NaCl, 50 mM imidazole, 5% glycerin, and 0.05% Tween-20 [pH 8.0]) before elution by gravity. An enrichment column was used to concentrate the protein. Eluates were examined for protein expression by SDS-PAGE and Coomassie blue staining.

### GST pull-down assay.

Recombinant proteins were incubated in GST binding buffer (25 mM Tris-HCl, 1 mM EDTA, 200 mM NaCl, 30 mM imidazole, and 0.01% NP-40 [pH 7.5]) at 4°C overnight. On the second day, GST beads were added to 30 μL of reaction mixture and incubated at 4°C for 2 hours. The beads were then washed 3 times with 1 mL of GST-binding buffer for 5 minutes. The protein interactions were detected by Western blot.

### Statistics.

Statistical analysis was performed using GraphPad Prism 8.0. Student’s 2-tailed paired *t* test and 1-way ANOVA with Tukey’s multiple-comparison test were used for comparison of differences where appropriate. For cancer-specific outcome analysis, Kaplan-Meier survival curves were used. *P* values less than 0.05 were considered significant.

### Study approval.

Animal assays was conducted following established standards to safeguard the welfare and health of the animals involved. Prior to commencing any experiments, approval was obtained from the Ethics Committee of West China Hospital after thorough deliberation to ensure compliance with ethical guidelines for experimental research. The ethical approval number for the animal experiments is 20220214016. Animal experiments were designed and conducted in accordance with the guidelines of the Ethics Committee of West China Hospital.

### Data availability.

The manuscript and the supplemental materials present all the data needed to evaluate the study’s conclusions. All data sets are available on the GEO with the accession no. GSE278226. All original data sets and analyses, including individual comparisons, are also reported in the Supporting Data Values file.

## Author contributions

JH conceived the project with the input from LZ and SZ. LZ, SZ, L Wang, X Liu, LQ, YZ, QH, and YM performed experimental biological research. X Liu was responsible for the RNA-Seq data analysis. XY helped collect human CRC samples. X Lei and L Wen were responsible for purchasing reagents and consumables. LZ, SZ, and JH completed the data sorting, data analysis, and data mapping. LZ and JH wrote the manuscript, and all authors reviewed and revised the manuscript. The authorship order was determined based on the relative contributions to the study, with those individuals most involved in the research listed first and the most experienced contributors last. The authorship order of the co–first authors is based on the relative contributions of their input to the final version of the manuscript.

## Supplementary Material

Supplemental data

Unedited blot and gel images

Supporting data values

## Version 1 11/12/2024

In-Press Preview

## Version 2 12/20/2024

Electronic publication

## References

[1] Bray F (2024). Global cancer statistics 2022: GLOBOCAN estimates of incidence and mortality worldwide for 36 cancers in 185 countries. CA Cancer J Clin.

[2] Keum N, Giovannucci E (2019). Global burden of colorectal cancer: emerging trends, risk factors and prevention strategies. Nat Rev Gastroenterol Hepatol.

[3] Knott SRV (2018). Asparagine bioavailability governs metastasis in a model of breast cancer. Nature.

[4] Garcia-Bermudez J (2018). Aspartate is a limiting metabolite for cancer cell proliferation under hypoxia and in tumours. Nat Cell Biol.

[5] Faubert B (2020). Metabolic reprogramming and cancer progression. Science.

[6] Christen S (2016). Breast cancer-derived lung metastases show increased pyruvate carboxylase-dependent anaplerosis. Cell Rep.

[7] Martinez-Outschoorn UE (2017). Cancer metabolism: a therapeutic perspective. Nat Rev Clin Oncol.

[8] Gong J (2020). Reprogramming of lipid metabolism in cancer-associated fibroblasts potentiates migration of colorectal cancer cells. Cell Death Dis.

[9] Luo XJ (2017). Emerging roles of lipid metabolism in cancer metastasis. Mol Cancer.

[10] Maan M (2018). Lipid metabolism and lipophagy in cancer. Biochem Biophys Res Commun.

[11] Yang Q (2018). Metabolites as regulators of insulin sensitivity and metabolism. Nat Rev Mol Cell Biol.

[12] Takagi A (1971). Lipid composition of sarcoplasmic reticulum of human skeletal muscle. Biochim Biophys Acta.

[13] Fullerton MD (2007). Developmental and metabolic effects of disruption of the mouse CTP: phosphoethanolamine cytidylyltransferase gene (Pcyt2). Mol Cell Biol.

[14] Calzada E (2019). Phosphatidylethanolamine made in the inner mitochondrial membrane is essential for yeast cytochrome bc(1) complex function. Nat Commun.

[15] Kersting MC (2004). Regulation of the yeast EKI1-encoded ethanolamine kinase by inositol and choline. J Biol Chem.

[16] Aoyama C (2002). Expression and characterization of the active molecular forms of choline/ethanolamine kinase-alpha and -beta in mouse tissues, including carbon tetrachloride-induced liver. Biochem J.

[17] Vance DE, Vance JE (2009). Physiological consequences of disruption of mammalian phospholipid biosynthetic genes. J Lipid Res.

[18] Wu Y (2019). Phospholipid remodeling is critical for stem cell pluripotency by facilitating mesenchymal-to-epithelial transition. Sci Adv.

[19] Guan Y (2020). The phosphatidylethanolamine biosynthesis pathway provides a new target for cancer chemotherapy. J Hepatol.

[20] Osawa T (2019). Phosphoethanolamine accumulation protects cancer cells under glutamine starvation through downregulation of PCYT2. Cell Rep.

[21] Gupta GP, Massague J (2006). Cancer metastasis: building a framework. Cell.

[22] Pastushenko I, Blanpain C (2019). EMT Transition States during tumor progression and metastasis. Trends Cell Biol.

[23] Lamouille S (2014). Molecular mechanisms of epithelial-mesenchymal transition. Nat Rev Mol Cell Biol.

[24] Bolos V (2003). The transcription factor Slug represses E-cadherin expression and induces epithelial to mesenchymal transitions: a comparison with Snail and E47 repressors. J Cell Sci.

[25] Cano A (2000). The transcription factor snail controls epithelial-mesenchymal transitions by repressing E-cadherin expression. Nat Cell Biol.

[26] Yang J (2004). Twist, a master regulator of morphogenesis, plays an essential role in tumor metastasis. Cell.

[27] Comijn J (2001). The two-handed E box binding zinc finger protein SIP1 downregulates E-cadherin and induces invasion. Mol Cell.

[28] Batlle E (2000). The transcription factor snail is a repressor of E-cadherin gene expression in epithelial tumour cells. Nat Cell Biol.

[29] Schaller MD (2001). Paxillin: a focal adhesion-associated adaptor protein. Oncogene.

[30] Pavlovic Z (2014). Isoform-specific and protein kinase C-mediated regulation of CTP:phosphoethanolamine cytidylyltransferase phosphorylation. J Biol Chem.

[31] Rama N (2009). Lung metastases from colorectal cancer: surgical resection and prognostic factors. Eur J Cardiothorac Surg.

[32] Giannis D (2020). The role of liver transplantation for colorectal liver metastases: a systematic review and pooled analysis. Transplant Rev (Orlando).

[33] Wei CR (2018). The role of Hippo signal pathway in breast cancer metastasis. Onco Targets Ther.

[34] Ma S (2019). The Hippo pathway: biology and pathophysiology. Annu Rev Biochem.

[35] Meng ZP (2016). Mechanisms of Hippo pathway regulation. Genes Dev.

[36] Harvey KF (2013). The Hippo pathway and human cancer. Nat Rev Cancer.

[37] Ji L (2020). LINC01413/hnRNP-K/ZEB1 axis accelerates cell proliferation and EMT in colorectal cancer via inducing YAP1/TAZ1 translocation. Mol Ther Nucleic Acids.

[38] Yu M (2018). YAP1 contributes to NSCLC invasion and migration by promoting Slug transcription via the transcription co-factor TEAD. Cell Death Dis.

[39] Dey A (2020). Targeting the Hippo pathway in cancer, fibrosis, wound healing and regenerative medicine. Nat Rev Drug Discov.

[40] Yu FX, Guan KL (2013). The Hippo pathway: regulators and regulations. Genes Dev.

[41] Zhao JM (2011). 15-Lipoxygenase 1 interacts with phosphatidylethanolamine-binding protein to regulate MAPK signaling in human airway epithelial cells. Proc Natl Acad Sci U S A.

[42] Bernier I, Jolles P (1984). Purification and characterization of a basic 23 kDa cytosolic protein from bovine brain. Biochim Biophys Acta.

[43] Banfield MJ (1998). Function from structure? The crystal structure of human phosphatidylethanolamine-binding protein suggests a role in membrane signal transduction. Structure.

[44] Lamiman K (2014). Survey of Raf kinase inhibitor protein (RKIP) in multiple cancer types. Crit Rev Oncog.

[45] Fu Z (2006). Metastasis suppressor gene Raf kinase inhibitor protein (RKIP) is a novel prognostic marker in prostate cancer. Prostate.

[46] Rajkumar K (2016). Understanding perspectives of signalling mechanisms regulating PEBP1 function. Cell Biochem Funct.

[47] Al-Mulla F (2013). RKIP: much more than Raf kinase inhibitory protein. J Cell Physiol.

[48] Prieto J (2016). Early ERK1/2 activation promotes DRP1-dependent mitochondrial fission necessary for cell reprogramming. Nat Commun.

[49] Ribeiro PS (2010). Combined functional genomic and proteomic approaches identify a PP2A complex as a negative regulator of Hippo signaling. Mol Cell.

[50] Chen R (2019). STRIPAK integrates upstream signals to initiate the Hippo kinase cascade. Nat Cell Biol.

[51] Vaz FM (2019). Mutations in PCYT2 disrupt etherlipid biosynthesis and cause a complex hereditary spastic paraplegia. Brain.

[52] Tang D (2019). The molecular machinery of regulated cell death. Cell Res.

[53] Zhang L (2010). Proteomic analysis reveals molecular biological details in varioliform gastritis without Helicobacter pylori infection. World J Gastroenterol.

[54] Zheng YG (2017). Homeostatic control of Hpo/MST kinase activity through autophosphorylation-dependent recruitment of the STRIPAK PP2A phosphatase complex. Cell Rep.

[55] Liu CY (2011). PP1 cooperates with ASPP2 to dephosphorylate and activate TAZ. J Biol Chem.

[56] Lv XB (2015). PARD3 induces TAZ activation and cell growth by promoting LATS1 and PP1 interaction. EMBO Rep.

[57] Wang WQ (2012). PTPN14 is required for the density-dependent control of YAP1. Genes Dev.

[58] Zhou RY (2021). The protein phosphatase PPM1A dephosphorylates and activates YAP to govern mammalian intestinal and liver regeneration. PLoS Biol.

[59] Inoue C (2023). PPP1R12A is a recycling endosomal phosphatase that facilitates YAP activation. Sci Rep.

[60] Hwang JY, Pallas DC (2014). STRIPAK complexes: structure, biological function, and involvement in human diseases. Int J Biochem Cell Biol.

[61] Kochall S (2017). Isolation of circulating tumor cells in an orthotopic mouse model of colorectal cancer. J Vis Exp.

[62] Meng Y (2022). Targeting CRL4 suppresses chemoresistant ovarian cancer growth by inducing mitophagy. Signal Transduct Target Ther.

[63] Wu J (2024). KDM6A-SND1 interaction maintains genomic stability by protecting the nascent DNA and contributes to cancer chemoresistance. Nucleic Acids Res.

[64] Mao X (2023). Requirement of WDR70 for POLE3-mediated DNA double-strand breaks repair. Sci Adv.

