# Supplementary material for: PCYT2 inhibits epithelial-mesenchymal transition in colorectal cancer by elevating YAP1 phosphorylation
Source: JCI Insight. 2024 Dec 20;9(24):e178823. doi: 10.1172/jci.insight.178823 (PMC11665560; doi:10.1172/jci.insight.178823)
Supplement: Supplemental data [file jciinsight-9-178823-s247.pdf]

Supplementary data

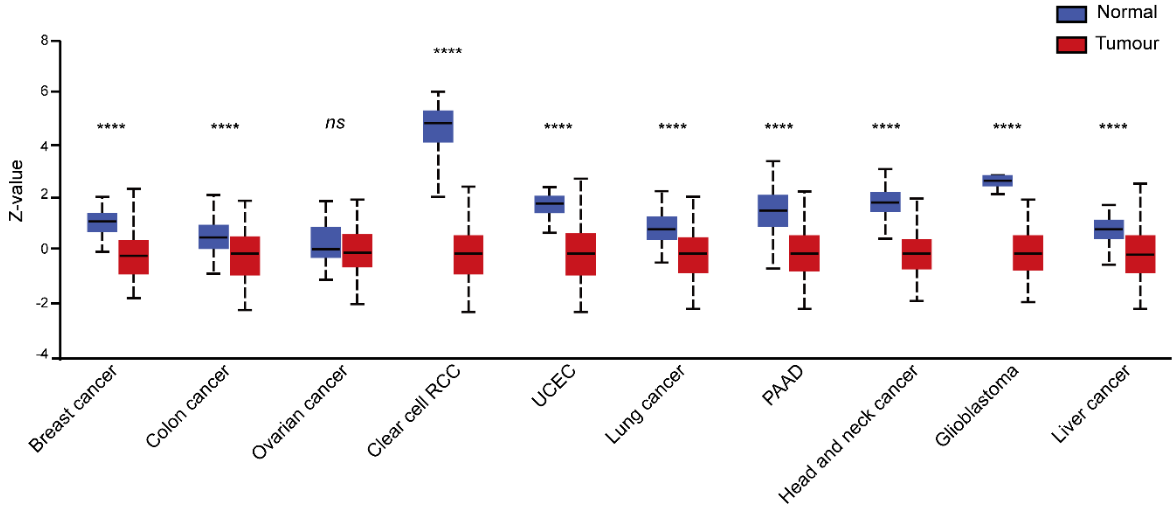

Supplementary Figure 1 (Related to Figure 1). The analysis of PCYT2 expression in pan cancer tissues.

The CPTAC database showed the lower expression of PCYT2 in pan cancers including breast cancer, Colon cancer, clear cell RCC, UCEC, Lung cancer, PAAD, Head and neck, Glioblastoma liver cancer. *NS* represent no significant difference, **** $P < 0.0001$ by 2-tailed unpaired Student's t test between genotypes.

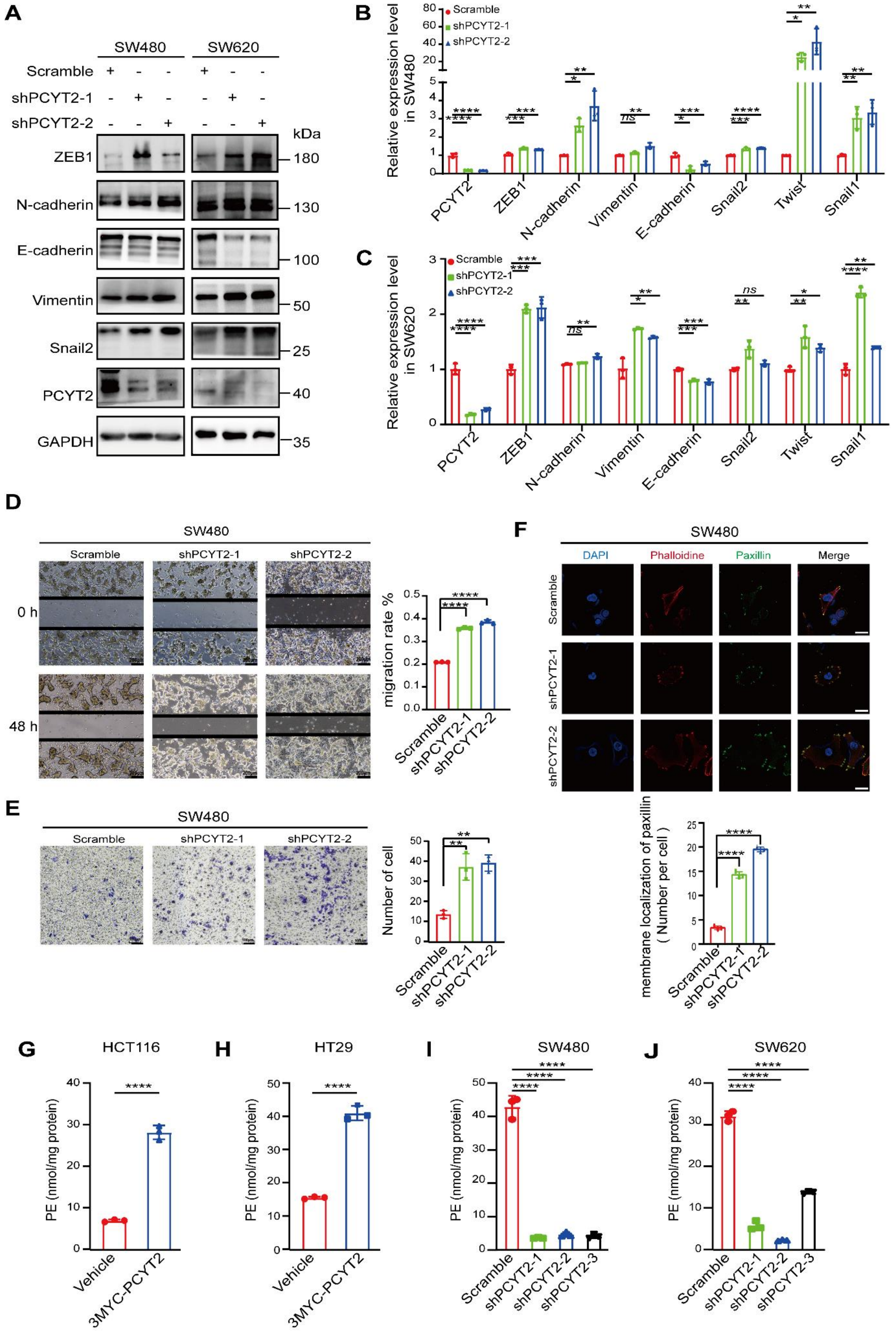
A
SW480
SW620
Scramble
shPCYT2-1
shPCYT2-2
kDa
ZEB1
N-cadherin
E-cadherin
Vimentin
Snail2
PCYT2
GAPDH
180
130
100
50
25
40
35
B
Relative expression level in SW480
Scramble
shPCYT2-1
shPCYT2-2
PCYT2
ZEB1
N-cadherin
Vimentin
E-cadherin
Snail2
Twist
Snail1
ns
C
Relative expression level in SW620
D
SW480
0 h
48 h
migration rate %
E
Number of cell
F
DAPI
Phalloidine
Paxillin
Merge
membrane localization of paxillin (Number per cell)
G
HCT116
H
HT29
I
J
PE (nmol/mg protein)
Vehicle
3MYC-PCYT2
shPCYT2-3

Supplementary Figure 2 (Related to Figure 2). PCYT2 knockdown promotes EMT and cell migration in CRC cell lines. (**A**) Detection of the epithelial and mesenchymal markers in PCYT2-knockdown SW620 and SW480 cells by Western blot. (**B** and **C**) Quantitative real-time PCR analysis for the epithelial and mesenchymal markers in PCYT2-knockdown SW480 (**B**) and SW620 (**C**) cells. (**D**) Detection of the cell migration ability in PCYT2-knockdown SW480 cells using scratch wound assay. The graph shows the area of wounds evaluated with Image J. Scale bar, 200 μm. Left: representative images, Right: quantifications. (**E**) Detection of the cell migration ability in PCYT2 knockdown cells using Transwell migration assays. Scale bar, 100 μm. Left: representative images, Right: quantifications. (**F**) Immunofluorescence assays were performed to determine the membrane localization of paxillin in PCYT2-knockdown SW480 cells. Different colors represent different antibodies or dyes: DAPI (blue), paxillin (green), phalloidine (red). Scale bar, 20 μm. Upper: representative images, Lower: statistical chart. (**G** and **H**) Detection of cellular PE levels in HCT116 (**G**) and HT29 (**H**) cells overexpressing PCYT2. (**I** and **J**) Detection of cellular PE levels in SW480 (**I**) and SW620 (**J**) cells with PCYT2-knockdown. Data are mean ± SD (n=3). Statistical significance was assessed with 1-way ANOVA with Tukey's multiple-comparison test (**B**-**F** and **I**-**J**) or 2-tailed unpaired Student's t test (**G**-**H**). *NS* represent no significant difference, * $P < 0.05$, ** $P < 0.01$, *** $P < 0.001$, **** $P < 0.0001$.

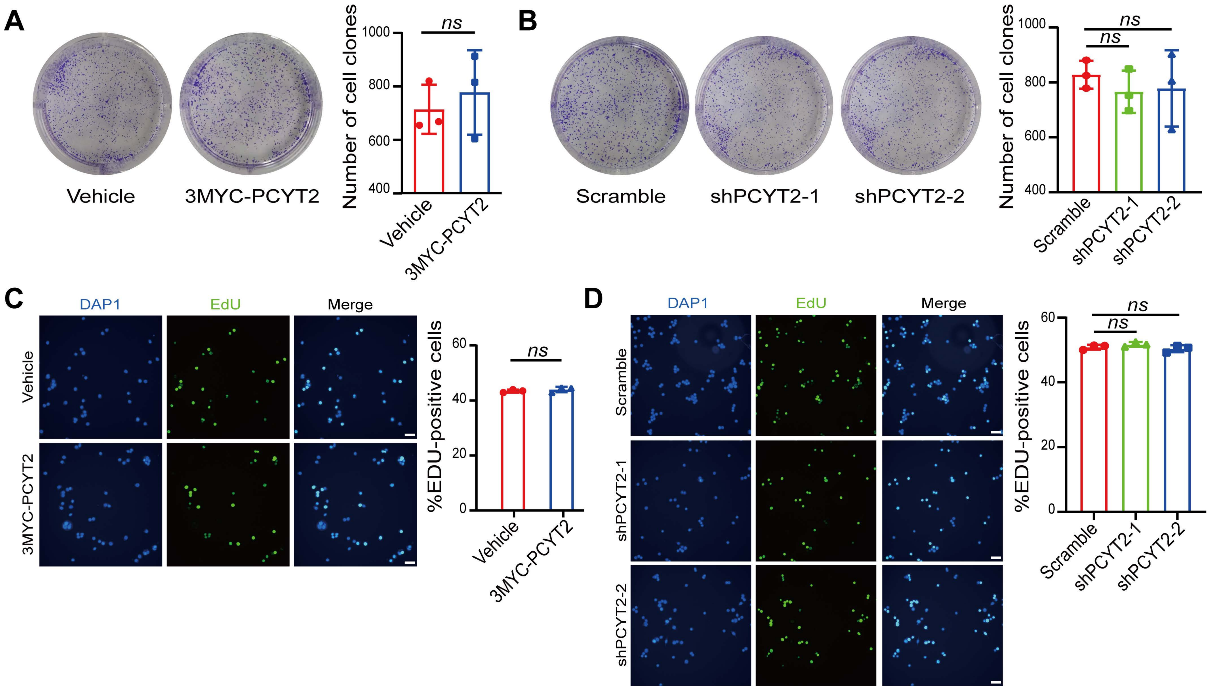

Supplementary Figure 3 (Related to Figure 2). PCYT2 does not affect the proliferation of CRC cells. (**A** and **B**) Representative images of colony formation of PCYT2-overexpressing (**A**) and knockdown (**B**) CRC cells. Left: representative images, Right: quantifications. (**C** and **D**) Detection of the proliferation ability in PCYT2-overexpressing (**C**) and knockdown (**D**) CRC cells by EdU incorporation assay. Left: representative images, Right: quantifications. Scale bar, 100 µm. Data are mean ± SD (n=3). *P* value was calculated with 2-tailed unpaired Student's *t* test (**A** and **C**) or 1-way ANOVA with Tukey's multiple-comparison test (**B** and **D**), *NS* represent no significant difference.

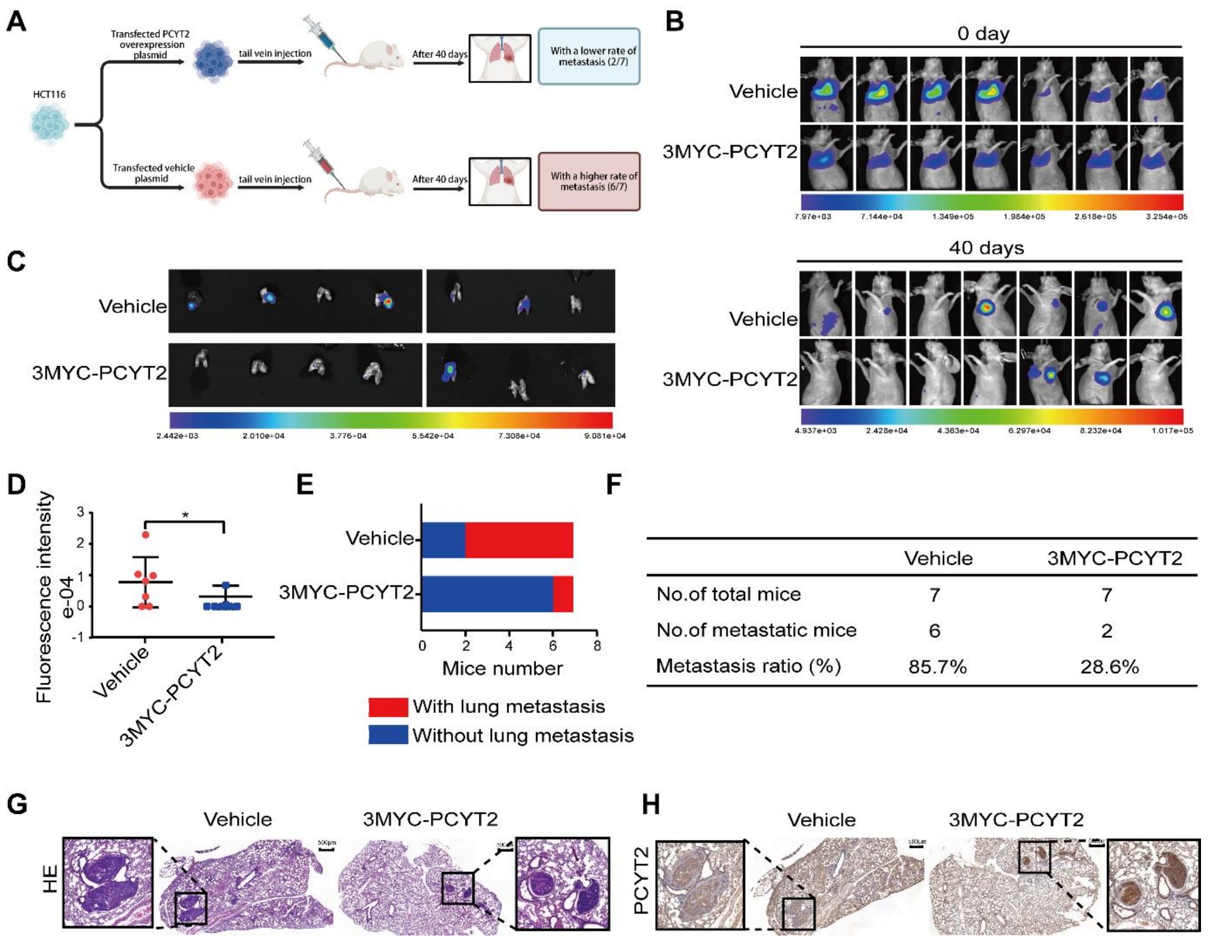

Supplementary Figure 4 (Related to Figure 3). Overexpression of PCYT2 inhibits lung metastasis in mice. (**A**) Schematic diagram demonstrating mouse tail vein injection models of CRC lung metastasis. (**B**) Images of lung fluorescence in mouse before and after injection of CRC cells. (**C** and **D**) The fluorescence intensity of lung was measured 40 days after tumor cell injection following dissection. (**E** and **F**) The tumorigenesis rate was observed 40 days after the injection of tumor capital into the caudal vein. (**G** and **H**) Representative images of H&E (**G**) and IHC (**H**) staining for the lung metastases. Scale bar, 500 µm. *NS* represent no significant difference, * $P < 0.05$ by 2-tailed unpaired Student's *t* test between genotypes.

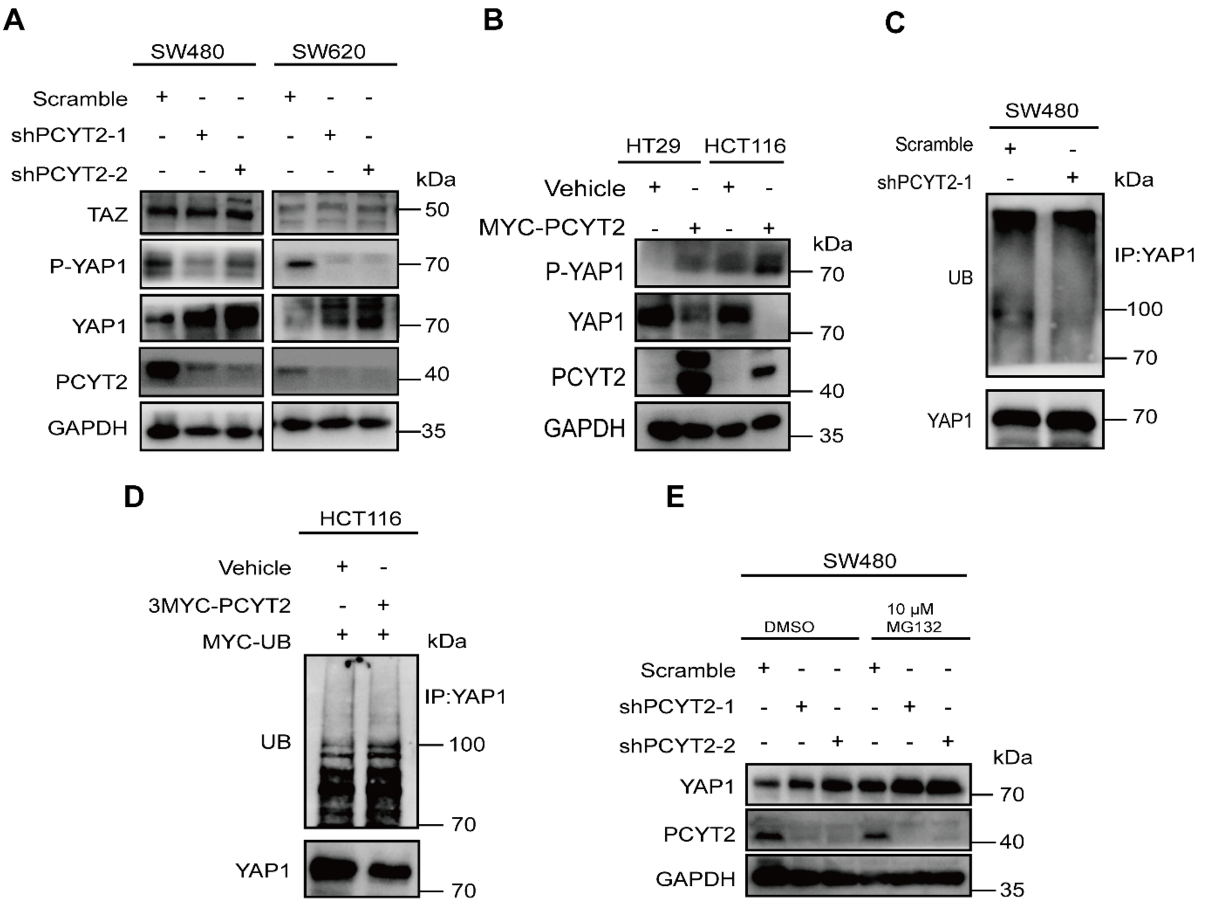

Supplementary Figure 5 (Related to Figure 4). PCYT2 remarkably increase phosphorylated YAP1 and promote P-YAP1 degradation. (**A**) Detection of YAP1/p-YAP1 expression in cells lacking PCYT2 by Western blot. (**B**) Detection of YAP1/p-YAP1 expression in cells overexpressing PCYT2 by Western blot. (**C** and **D**) Detection of YAP1 ubiquitination in cells with either PCYT2 knockdown (**C**) or PCYT2 overexpression (**D**) by Western blot. (**E**) Detection of YAP1 expression in PCYT2-knockdown SW480 cells treated with or without MG132 by Western blot.

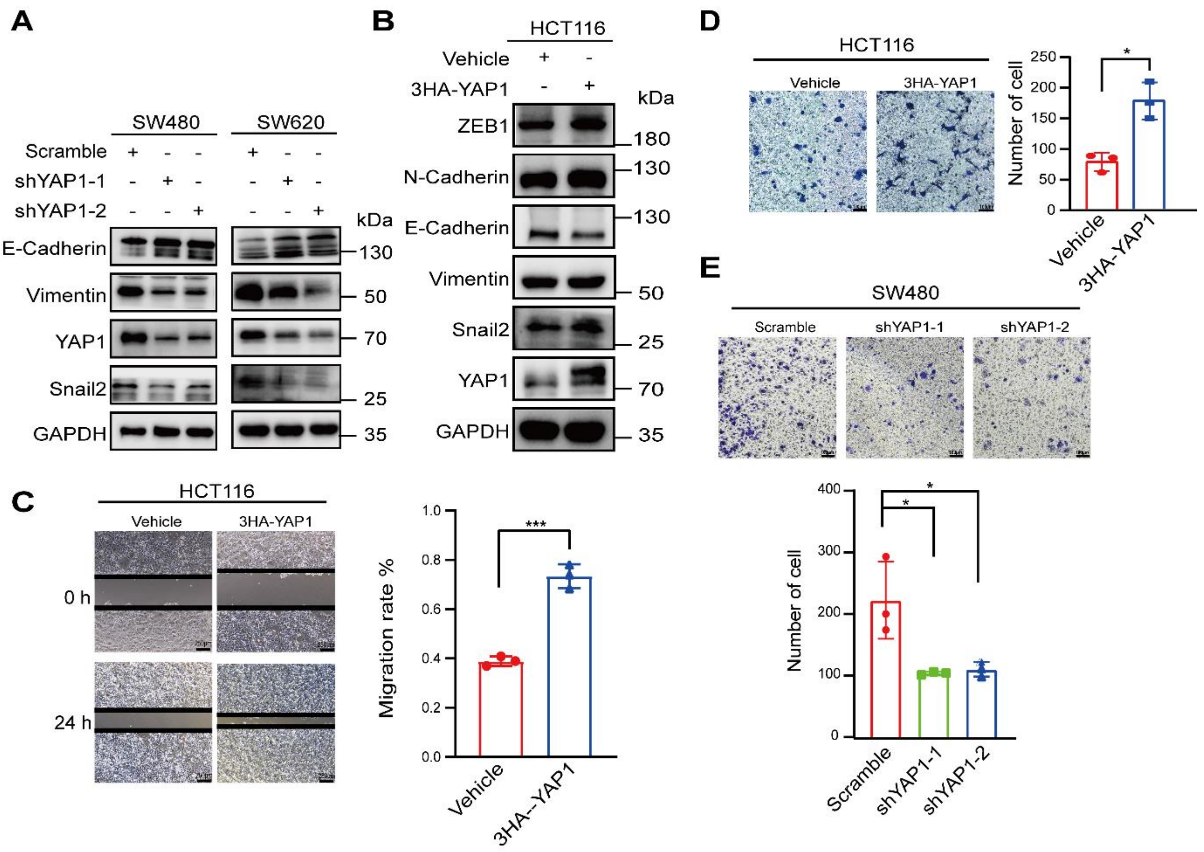

Supplementary Figure 6 (Related to Figure 5). YAP1 promotes colorectal cancer cell migration. (**A** and **B**) Detection of the epithelial and mesenchymal markers in YAP1-knockdown (**A**) or YAP1-overexpressing (**B**) CRC cells. (**C**) Scratch assays for detection of the migration capability in HCT116 cells overexpressing 3HA-YAP1. The graph shows the area of a wound evaluated with Image J. Scale bar: 250 μm. Left: representative images, Right: quantifications. (**D** and **E**) Transwell assays for detection of the migration capability in YAP1-overexpressing (**D**) HCT116 cells or YAP1-knockdown (**E**) SW480 cells. Left in D and Upper in E: representative images, Right in D and Lower in E: quantifications. Scale bar, 100 μm. Data represent the mean ± SD (n=3). Statistical significance was assessed with 2-tailed unpaired Student's t test (**C-D**) or 1-way ANOVA with Tukey's multiple-comparison test (**E**), * $P < 0.05$, *** $P < 0.001$.

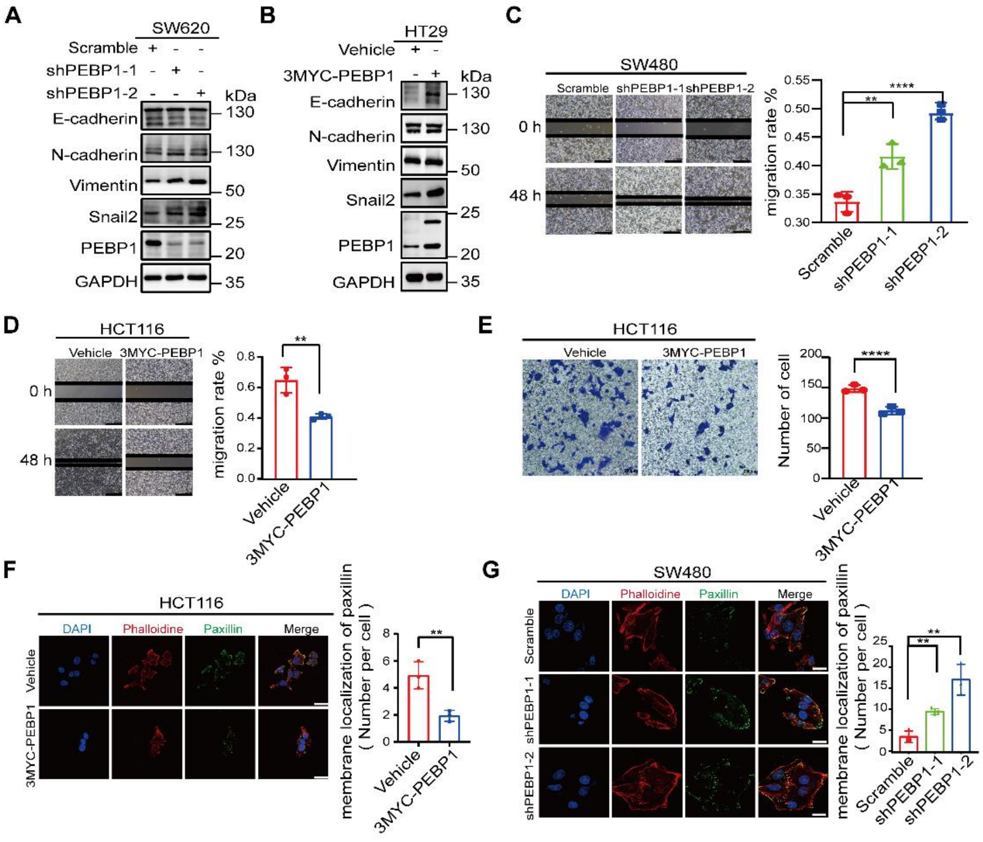

Supplementary Figure 7 (Related to Figure 5). PEBP1 inhibits colorectal cancer cell metastasis. (**A** and **B**) Western blot was performed to detect epithelial and mesenchymal markers in PEBP1-knockdown SW620 cells (**A**) and PEBP1 -overexpressing HT29 cells (**B**). (**C** and **D**) Representative images of scratch assay in PEBP1-knockdown SW480 cells (**C**) and PEBP1-overexpressing HCT116 cells (**D**). Scale bar, 100 μm. Left: representative images, Right: quantifications. (**E**) Transwell assays for detection of the migration capability in PEBP1-overexpressing HCT116 cells. Scale bar, 100 μm. Left: representative images, Right: quantifications. (**F**) Immunofluorescence assay was performed to determine the membrane localization of paxillin in HCT116 cells with PEBP1-overexpressing. Scale bar, 20 μm. Left: representative images, Right: quantifications. (**G**) Immunofluorescence assay was performed to determine the membrane localization of paxillin in SW480 cells with PEBP1-knockdown. Scale bar, 20 μm. Left: representative images, Right: quantifications. Data represent the mean ± SD (n=3). Statistical significance was assessed with 1-way ANOVA with Tukey's multiple-comparison test (**C** and **G**) or 2-tailed unpaired Student's t test (**D-F**), ** *P*

< 0.01, **** $P < 0.0001$.

Supplementary Table 1. Differentially expressed genes in PCYT2 knockdown CRC cells.

**Downregulated**			
PCYT2	UPK3BL1	HCG4	LOC254896
CNPY3-GNMT	KCNK3	SIGLEC6	NIPAL4
DNAAF4-CCPG1	RNF182	RNA18SN3	INSL3
CLCNKB	KRT71	TPM1-AS	CBR3
HSPB2-C11orf52	JMJD1C-AS1	CA5BP1-CA5B	CHST6
GTF2H2B	C16orf86	MEFV	DGCR9
SNHG22	NXNL2	CHRNA10	IRF1-AS1
LOC389199	APLNR	LSMEM2	GNG11
RPL34-AS1	IL3RA	CPXM1	TNS1
LINC01659	TMPRSS4	MSH5	MLPH
RPS6KA2-AS1	NUAK1	SPTSSB	C11orf96
C11orf86	WNT9B	LINC02175	FAM131B
C1QTNF8	CHRNA4	VAT1L	LOC100133286
PLA2G4E-AS1	COL2A1	PROZ	DMGDH
C5orf66-AS1	SPON1	LOC100506282	FOXD1
LINC01694	CYP26A1	EEF1E1-BLOC1S5	LRP4-AS1
MIR10394	FAM95C	STX16-NPEPL1	F8A3
KLF1	APOC4-APOC2	CLDN5	LINC01297-DUXAP10-NBEAP6
XPNPEP2	ALDH3A1	HAPLN3	LINC02563
DNMT3L-AS1	TRIM54		

Upregulated

SCOC-AS1	C1QTNF3-AMACR	NKAIN4	LOC108783645
LOC105274304	CSNK1G2-AS1	WDR93	USP27X-AS1
MBOAT4	HEPACAM2	LINC00323	LOC100506124
GNMT	FAAHP1	LOC101927583	CASC21
COL10A1	LOC101927310	C11orf16	GALR3
LOC105371925	GAB3	LOC107985770	FAM13A-AS1
C5AR2	SLCO6A1	PTGIR	KRT79
CAPNS2	HMSD	GLT1D1	CYP4B1
CXCR3	BANF2	TAS2R31	LOC105372990
TRIM34	LOC645752	LOC100507053	PEX5L
HLA-DRB1	FLJ12825	FGD2	C22orf24
ADGRB3	LOC101929882	EPN3	RBFADN
NCKAP5-AS2	RGMB-AS1	FRG2	PCDHGC5
TM4SF18	SPDYE13	SPANXC	FAM230C
ERV3-1-ZNF117	MSANTD3-TMEFF1	NOTCH4	RBM14-RBM4
STIMATE	TMEM144	UGT1A3	GPRASP2
IL23A	KIAA1614-AS1	INHBA	CHRM5
AGA	LINC00648	KRTAP2-4	GPRIN3
TAGLN	COL4A5	PLAC8	C4orf50
CXCL8	LINC01611	PLAAT4	HHIPL2
CHGB	ROBO4	PTPRM	FLJ30679

ZNF624	PLAU	ZBED3-AS1	COMMD3-BMI1
OCLNP1	FSD2	LINC01405	LINC00598
ZNF561-AS1	TBC1D7-LOC100130357	SPRR2D	ECI2-DT
FAM157B	CHGA	KRT40	SCARNA6
SNAI2	PCDHGA9	ZEB1	LINC00426
ST3GAL5	TIE1	PAPSS2	DACH2
HTR7P1	H2BC17	GZMB	NUDT9P1
PLA2G4C	ADD3-AS1	SCG2	PRR20E
IL1RAP	LINC01602	RARB	ALLC
CXCL1	KRT34	BIRC7	HCG9
LETM2	XCR1	HDAC11-AS1	INHBA-AS1
CFL2	ITGB3	DUSP19	MYLK3
GABRB2	CSDC2	DUSP13	FSTL1
E2F7	LINC01269	ZNF8-ERVK3-1	ISY1-RAB43
LOC101927919	CCN2	CD274	PRSS1
GOLGA8S			

Supplementary Table 2. PEBP1 interacting proteins identified by MS

Protein	Protein	Protein	Protein	Protein
PEBP1	TLK2	IGKV4-1	MED15	RPL10A
TACC2	ZAK	EML1	HSP90AB2P	LSG1
CSDE1	PKP3	HNRNPUL1	TBL3	RPS3A
MARK2	RPS5	NEDD1	EIF3I	SERBP1
RPS7	RPS9	EIF2S1	Septin-1	ERLIN1
MYH7; MYH6	RPL3	ATAD3C	PGAM5	LRPPRC
RPL9	EIF3G	EIF3M	HNRNPR	HRNR
PURA	CCDC88C	KALRN	TRIOBP	SEC16A
NPEPPS	HAUS1	LARP1	PSPC1	NCL
MYH13	RAVER1	SEC23A	NCBP1	HSPA9
KANK2	HDLBP	RAF1	STAU2	HNRNPA1; HNRNPA1L2
ZNF207	PPP2R1A	TIAL1	MED14	FLG2
NIFK	CKAP5	ASCC3	TES	HNRNPF
DNAJA3	CREBBP	OR2V2	XRN2	RPL4
PRRC2B	SPATS2	EXOSC4	FUBP3	FAM98A
ZCCHC3	PDE8A	TJP3	ACADSB	HNRNPU
SUCLG1	CLTCL1	RPL15	TWF1	RRBP1
MCCC1	KLHL11	RAD54B	PTH2R	RBM14
EIF3L	MED16	DHX30	EIF3B	YWHAZ
EEF1B2	EIF3C; EIF3CL	EIF3D	EIF3A	HNRNPK
DHX29	PABPC4	G3BP2	HSD17B4	API5

VARS	DDX6	PABPC1	VAPB	ALB
DDX1	FXR2	G3BP1	EIF3F	NONO
PRKCA; PRKCB	IGF2BP3	USP10	EIF4E	RNMT
FXR1	PAWR	RPS6	EIF3E	STAU1
MOV10	FAM120A	SEC23B	PPFIA1	HNRNPH2
IKBKB	RPS3	UPF1	XRCC6	TAF15
HAUS3	EML4	RPL7	RPSA	FAM98B
UBAP2	DDX3X; DDX3Y	PRDX1; PRDX4	HNRNPA0	DHX15
YTHDF3	TRA2B	YBX1	RPS4X	HSP90AB1
IGF2BP2	FMR1	RPS2	EIF4G1	UBAP2L
NUFIP2	TTC27	NMNAT1	Septin-2	LMNA
YBX3	HAUS5	EEF1A2	EEF1A1P5; EEF1A1	RPL6
SEC13	SEC24A	HAUS6	HSP90AA	DDX5
SEC24D	SEC24C	RPL8	C14orf166	ATXN2L
PPFIBP1	EZR	RPS8	RTCB	EIF4A1
DHX9	EEF1D	FUS	GOLGA2	HNRNPA3
EWSR1	XRCC5	TMPO	HAUS8	TBC1D9B
EIF4A3	SLC25A11	STRAP	RBFOX1/2/3	TOP3B
EIF2S3; EIF2S3L	CAPRIN1	SYNCRIP	MAP4	HNRNPH1
KHDRBS1	SEC24B	KHSRP	PTBP1	ELAVL1
EIF4G2	PRRC2A/2C			

Supplementary Table 3. List of antibodies

Antibodies	Source	Identifier
E-cadherin	Proteintech	20874-1-AP
N-cadherin	Proteintech	22018-1-AP
Vimentin	Huaan biology	EM0401
Snail1	Proteintech	26183-1-AP
Snail2/SLUG	Proteintech	12129-1-AP
ZEB1	Proteintech	21544-1-AP
GAPDH	Nakasugi Golden Bridge	TA-08
Histone H3	Proteintech	17168-1-AP
PCYT2	Invitrogen	PA5-28047
PEBP1	Invitrogen	36-0700
Paxillin	Proteintech	10029-1-Ig
TAZ	Huaan biology	ER1917-51
YAP1	Proteintech	13584-1-AP
p-YAP1-S127	CST	13008
YAP1 for ChIP	CST	14074T
PPP2R1A	Proteintech	15882-1-AP
LATS1	ABclonal	A17992

Supplementary Table 4. List of primers and oligos

Primers for real time PCR		
ACTIN	forward	TGGAGAAAATCTGGCACCAC
	reverse	GAGGCGTACAGGGATAGCAC
PCYT2	forward	TCCTGCAGACATCTCARAAG
	reverse	CCTTGTAGTGATTGACCTCCTR
PEBP1	forward	CCTGCAAGAAGTGGACGAG
	reverse	ACCAAGGTGTAGAGCTTCCCT
N-cadherin	forward	CCTCCAGAGTTTACTGCCATGAC
	reverse	GTAGGATCTCCGCCACTGATTC
E-cadherin	forward	GCCTCCTGAAAAGAGAGTGGAAG
	reverse	TGGCAGTGTCTCTCCAAATCCG
Vimentin	forward	AGGCAAAGCAGGAGTCCACTGA
	reverse	ATCTGGCGTTCCAGGGACTCAT
Snail1	forward	TGCCCTCAAGATGCACATCCGA
	reverse	GGGACAGGAGAAGGGCTTCTC
Snail2	forward	ATCTGCGGCAAGGCGTTTTCCA
	reverse	GAGCCCTCAGATTTGACCTGTC
Twist	forward	GCCAGGTACATCGACTTCCTCT
	reverse	TCCATCCTCCAGACCGAGAAGG
ZEB1	forward	CATCTTGAGCTGAATTTGGGTAACA
	reverse	CCTGAAATGACCTGAAGCATGAA
YAP1	forward	TGTCCCAGATGAACGTCACAGC

	reverse	TGGTGGCTGTTTCACTGGAGCA
PPP2R1A	forward	ACCGCATGACTACGCTCTTCTG
	reverse	TTGAAGCGGACATTGGCAACCG

Primers for ChIP

ZEB1	forward	CGTAGAGCGAGAGCCTCTA
	reverse	TCTAAATGCTCGAGTCACCT
CTGF	forward	TTCTGTGAGCTGGAGTGTGC
	reverse	GCCAATGAGCTGAATGGAGT
Snail2	forward	AAACACTGACTGTCTTTCTT
	reverse	TTTTTGCACTGGTATTTCTT

Oligos for shRNA

shPCYT2-1	forward	CCGGGCAGACATCTCAGAAGATCATCTCGAGATGAT<br>CTTCTGAGATGTCTGCTTTTTG
	reverse	AATTCAAAAAGCAGACATCTCAGAAGATCATCTCG<br>AGATGATCTTCTGAGATGTCTGC
shPCYT2-2	forward	CCGGACTAGAGACCCTGGACAAATACTCGAGTATTT<br>GTCCAGGGTCTCTAGTTTTTTG
	reverse	AATTCAAAAAACTAGAGACCCTGGACAAATACTCG<br>AGTATTTGTCCAGGGTCTCTAGT
shPCYT2-3	forward	CCGGTCACGGCAAGACAGAAATTATCTCGAGATAAT<br>TTCTGTCTTGCCGTGATTTTTG
	reverse	AATTCAAAAATCACGGCAAGACAGAAATTATCTCG<br>AGATAATTTCTGTCTTGCCGTGA

shPEBP1-1	forward	CCGGGCCCTTGAGCCTGCAAGAAGTCTCGAGACTT<br>CTTGCAGGCTCAAGGGCTTTTTG
	reverse	AATTCAAAAAGCCCTTGAGCCTGCAAGAAGTCTCG<br>AGACTTCTTGCAGGCTCAAGGGC
shPEBP1-2	forward	CCGGCGAGCAGGACAGGCCGCTAAACTCGAGTTTA<br>GCGGCCTGTCCTGCTCGTTTTTG
	reverse	AATTCAAAAACGAGCAGGACAGGCCGCTAAACTCG<br>AGTTTAGCGGCCTGTCCTGCTCG
shPEBP1-3	forward	CCGGGTGGTCAACATGAAGGGCAATCTCGAGATTG<br>CCCTTCATGTTGACCACTTTTTG
	reverse	AATTCAAAAAGTGGTCAACATGAAGGGCAATCTCG<br>AGATTGCCCTTCATGTTGACCAC
shYAP1-1	forward	CCGGCCCAGTTAAATGTTCACCAATCTCGAGATTGG<br>TGAACATTTAACTGGGTTTTTG
	reverse	AATTCAAAAACCCAGTTAAATGTTCACCAATCTCGA<br>GATTGGTGAACATTTAACTGGG
shYAP1-2	forward	CCGGCAGGTGATACTATCAACCAAACTCGAGTTTGG<br>TTGATAGTATCACCTGTTTTTG
	reverse	AATTCAAAAACAGGTGATACTATCAACCAAACTCG<br>AGTTTGGTTGATAGTATCACCTG
shYAP1-3	forward	CCGGGACCAATAGCTCAGATCCTTTCTCGAGAAAG<br>GATCTGAGCTATTGGTCTTTTTG
	reverse	AATTCAAAAAGACCAATAGCTCAGATCCTTTCTCGA

		GAAAGGATCTGAGCTATTGGTC
shPPPAR1A-1	forward	CCGGCTACGCTCTTCTGCATCAATGCTCGAGCATTGATGCAGAAGAGCGTAGTTTTTG
	reverse	AATTCAAAAACTACGCTCTTCTGCATCAATGCTCGAGCATTGATGCAGAAGAGCGTAG
shPPPAR1A-2	forward	CCGGTTGCCAATGTCCGCTTCAATGCTCGAGCATTGAAGCGGACATTGGCAATTTTTG
	reverse	AATTCAAAAATTGCCAATGTCCGCTTCAATGCTCGAGCATTGAAGCGGACATTGGCAA
shPPPAR1A-3	forward	CCGGACCAGGATGTGGACGTCAAATCTCGAGATTTGACGTCCACATCCTGGTTTTTTG
	reverse	AATTCAAAAAACCAGGATGTGGACGTCAAATCTCGAGATTTGACGTCCACATCCTGGT
