# Supplementary material for: PCYT2 inhibits epithelial-mesenchymal transition in colorectal cancer by elevating YAP1 phosphorylation
Source: JCI Insight. 2024 Dec 20;9(24):e178823. doi: 10.1172/jci.insight.178823 (PMC11665560; doi:10.1172/jci.insight.178823)
Supplement: Unedited blot and gel images [file jciinsight-9-178823-s248.pdf]

PCYT2 inhibits epithelial-to-mesenchymal transition in colorectal cancer by elevating YAP1 phosphorylation

unedited images for all blots and gels in the manuscript associated with Figures and Supplementary figures.

Full unedited gel for Figure 1D

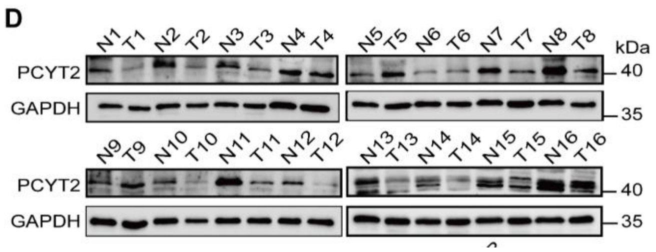

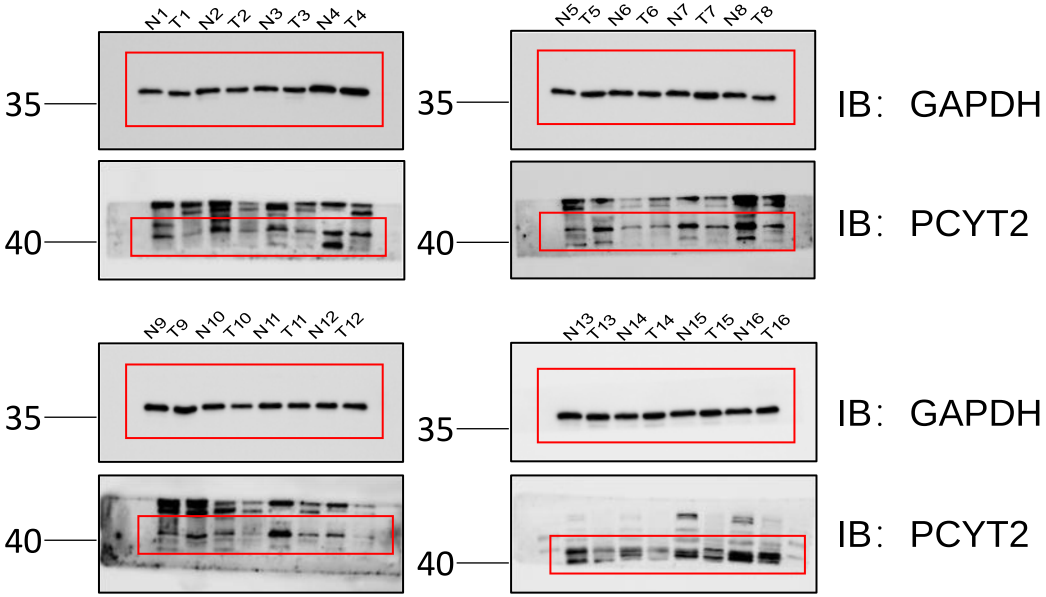

The blots come from the same batch of sample.

Full unedited gel for Figure 1F

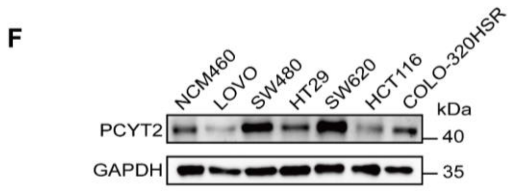

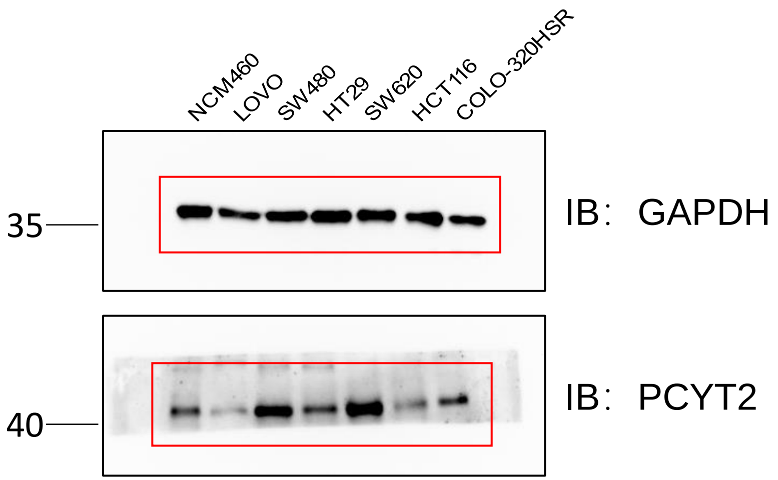

The blots come from the same batch of sample.

Full unedited gel for Figure 2A

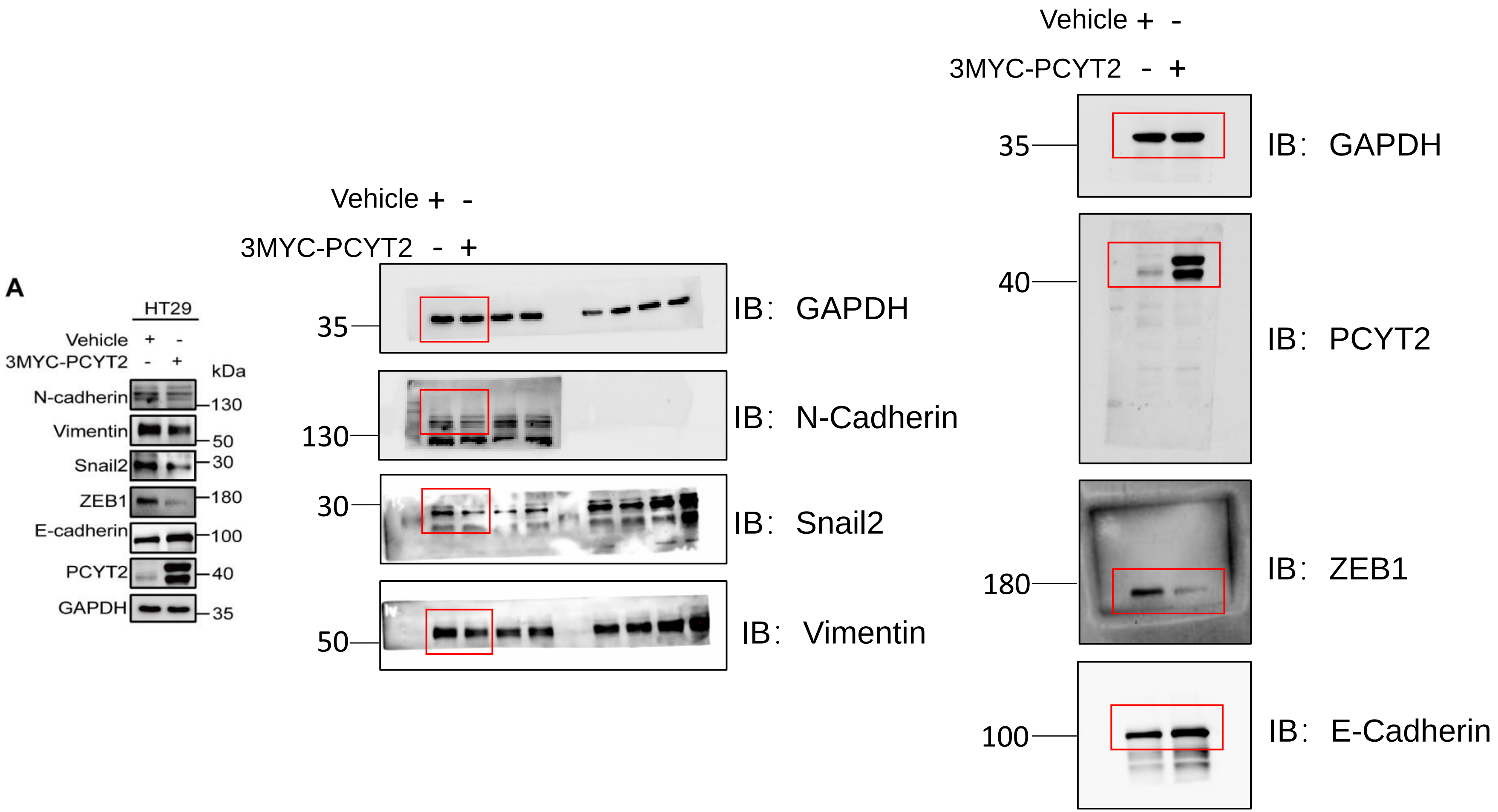

The blots come from the same batch of sample.

Full unedited gel for Figure 2B

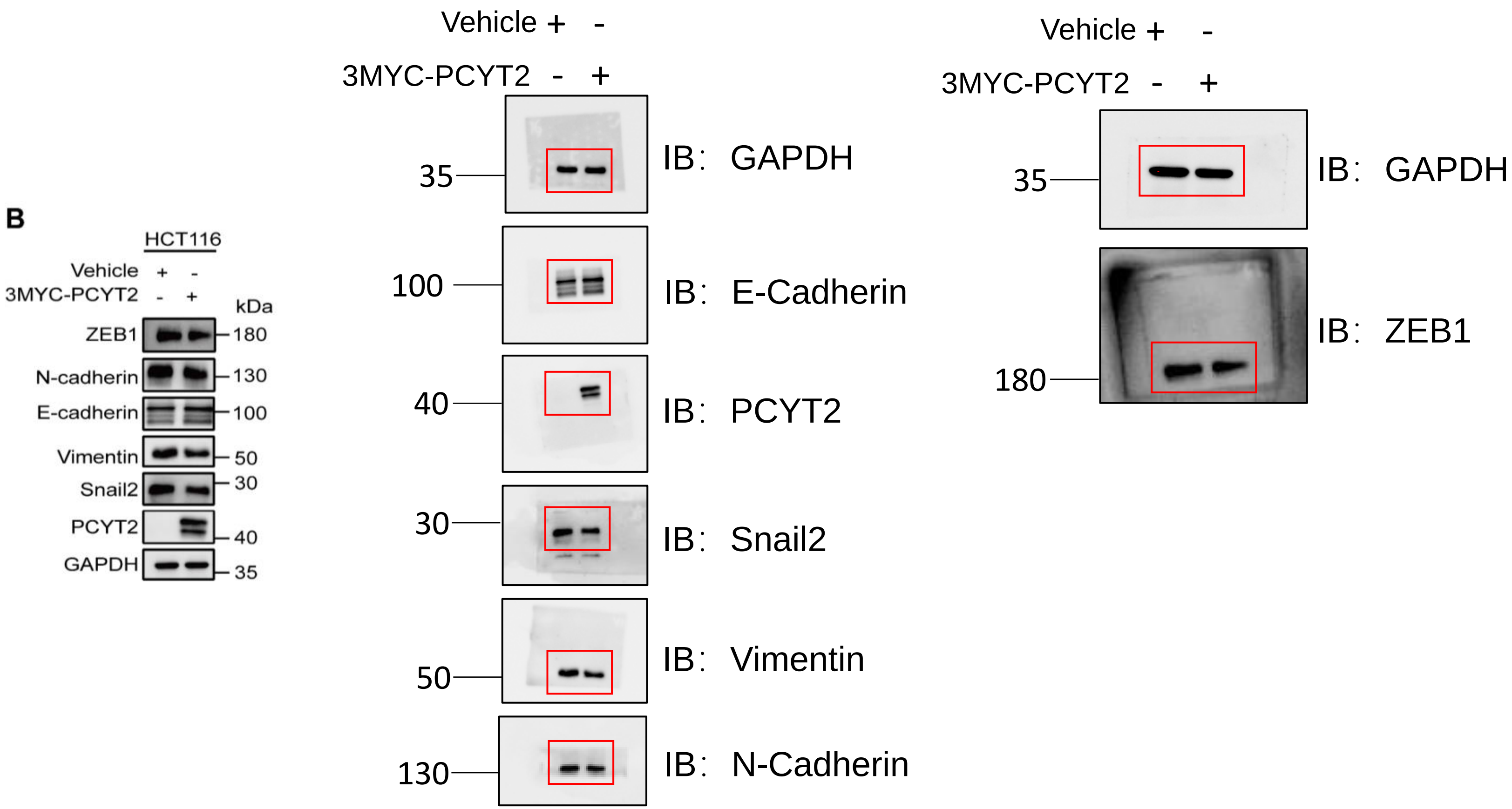

The blots come from the same batch of sample.

Full unedited gel for Figure 4D

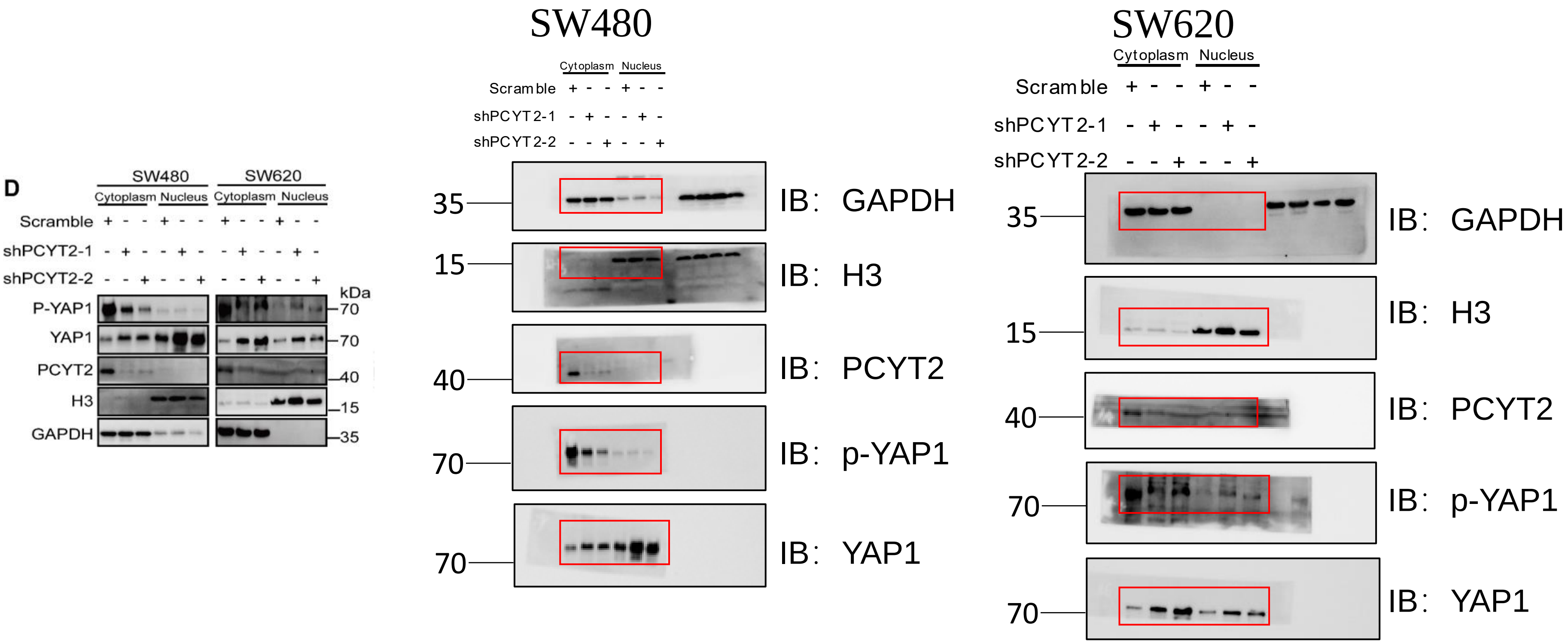

The blots come from the same batch of sample.

Full unedited gel for Figure 4D（Biological duplicate data）

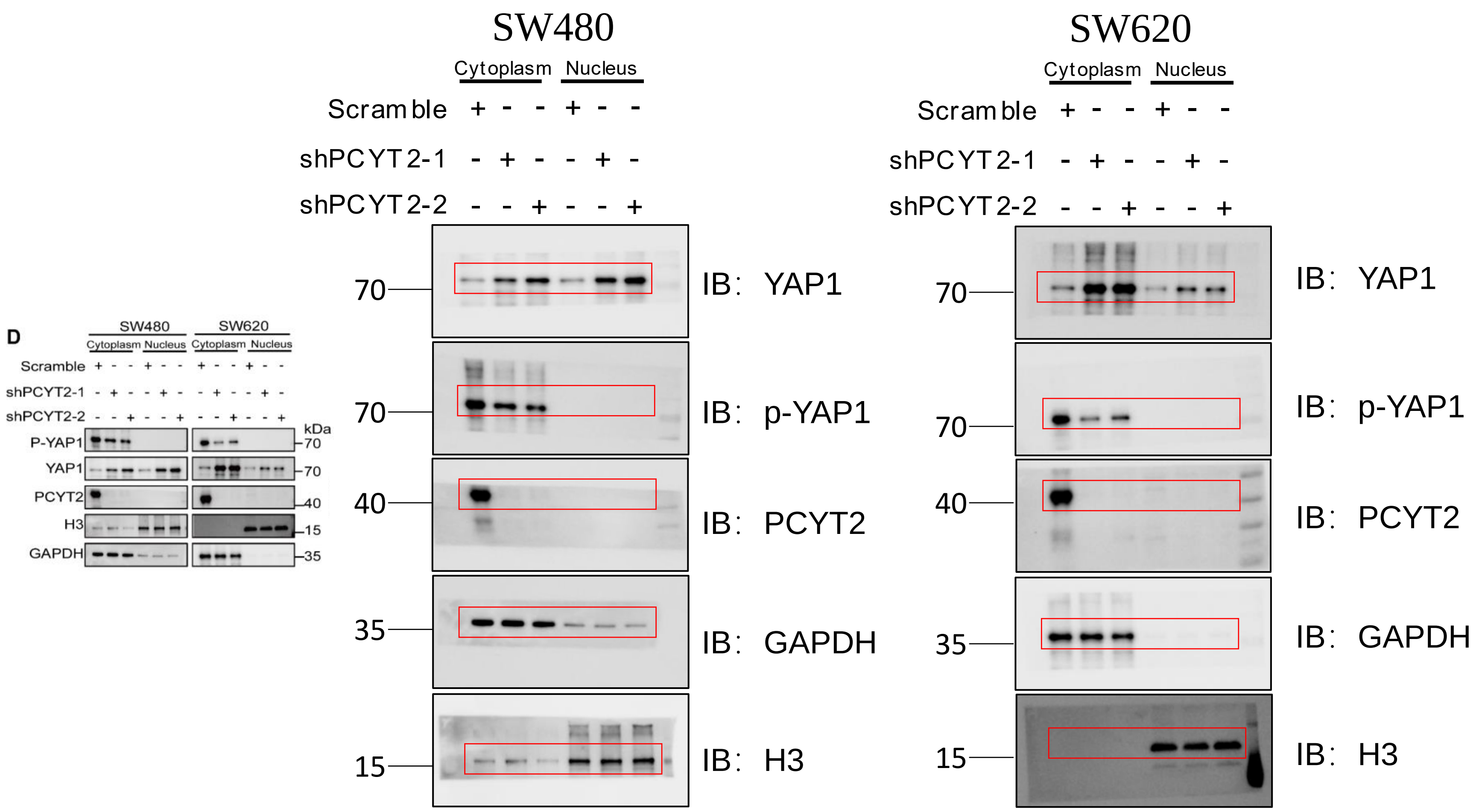

The blots come from the same batch of sample.

Full unedited gel for Figure 4E

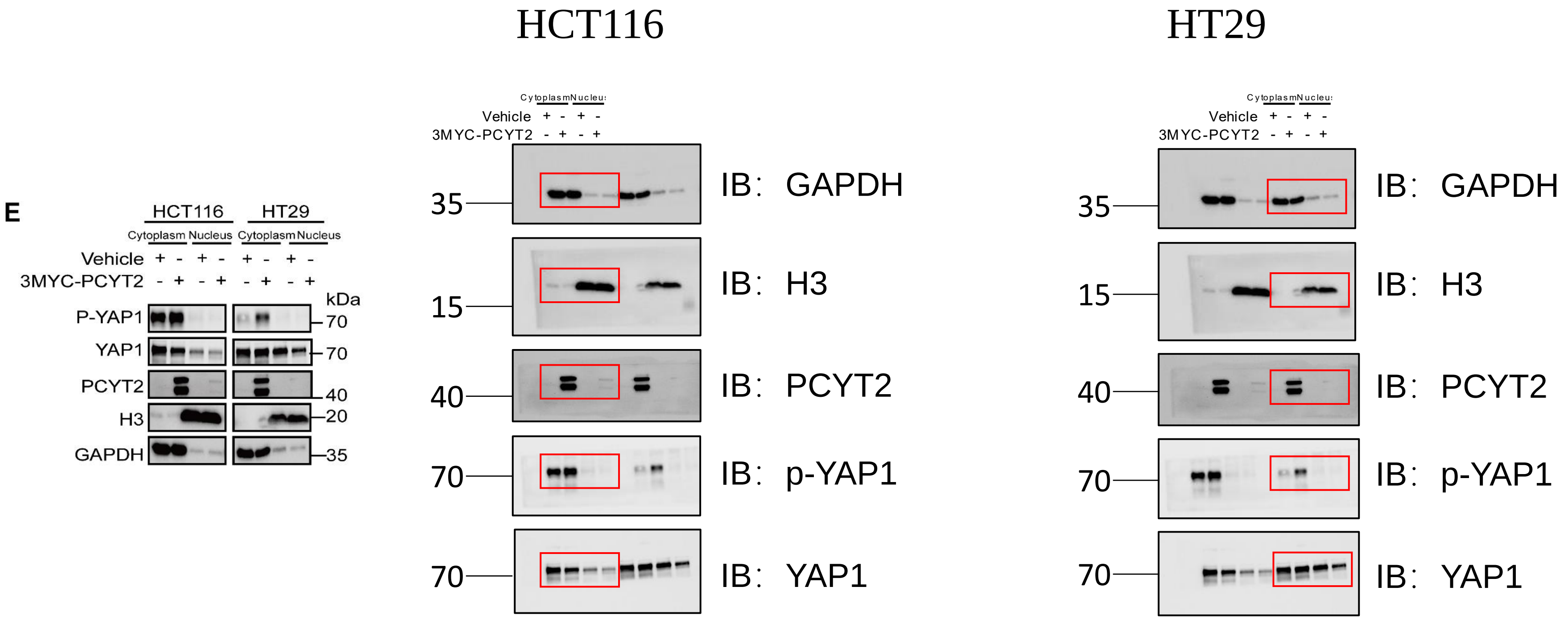

The blots come from the same batch of sample.

Full unedited gel for Figure 4H

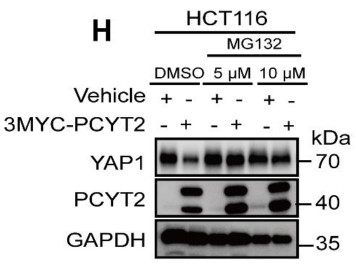

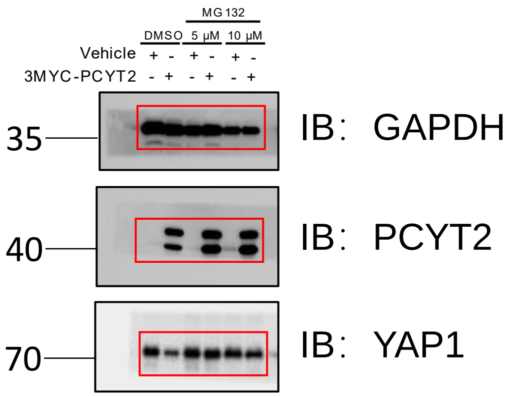

The blots come from the same batch of sample.

Full unedited gel for Figure 4I

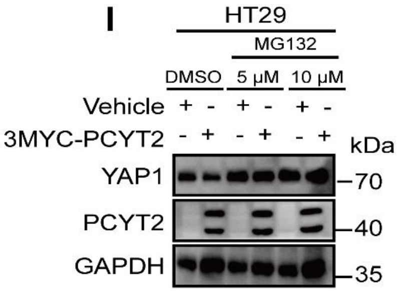

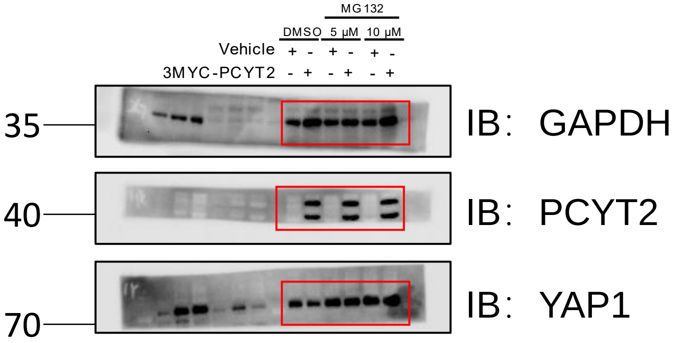

The blots come from the same batch of sample.

Full unedited gel for Figure 4 K

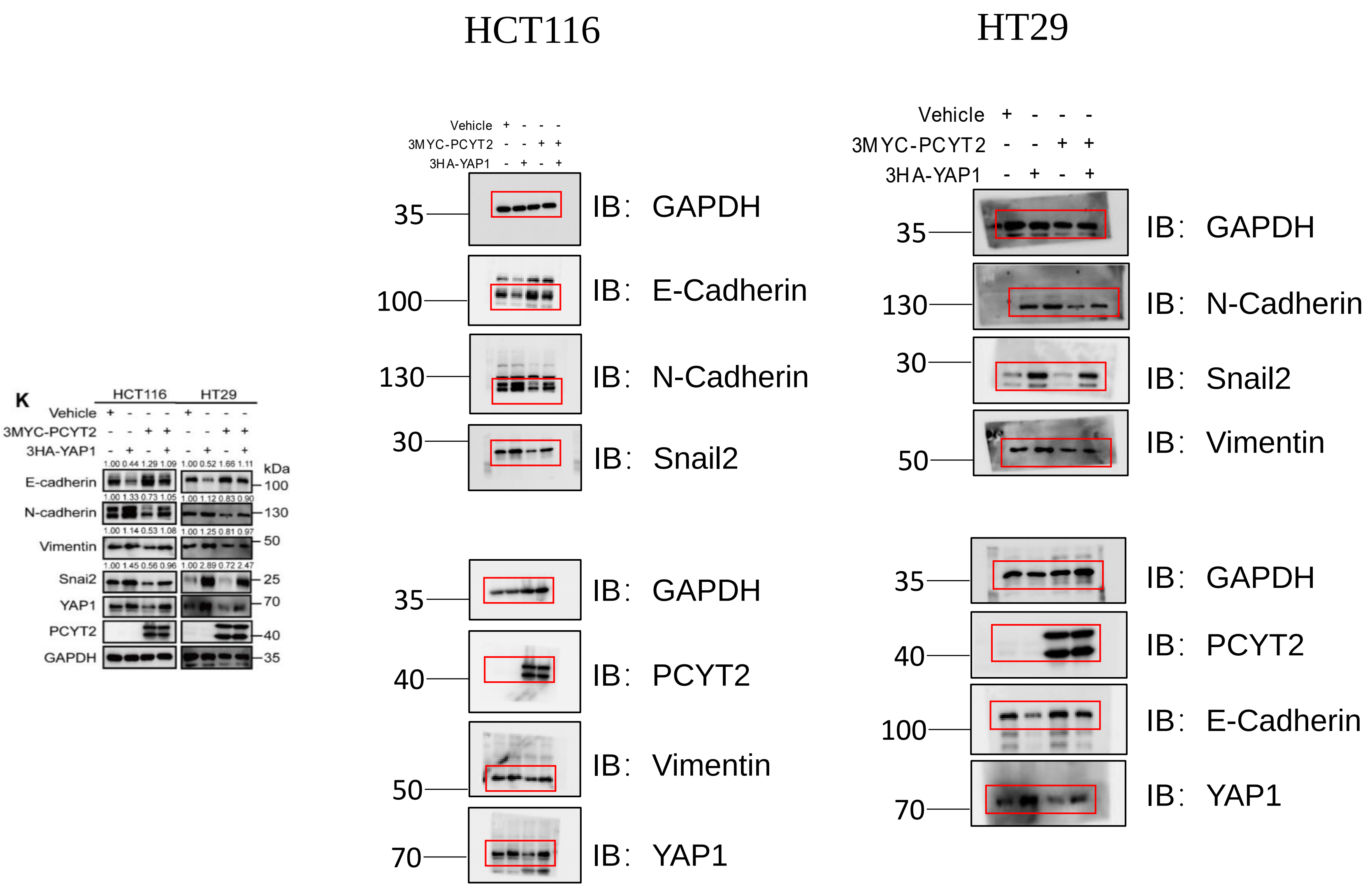

The blots come from the same batch of sample.

Full unedited gel for Figure 4 M

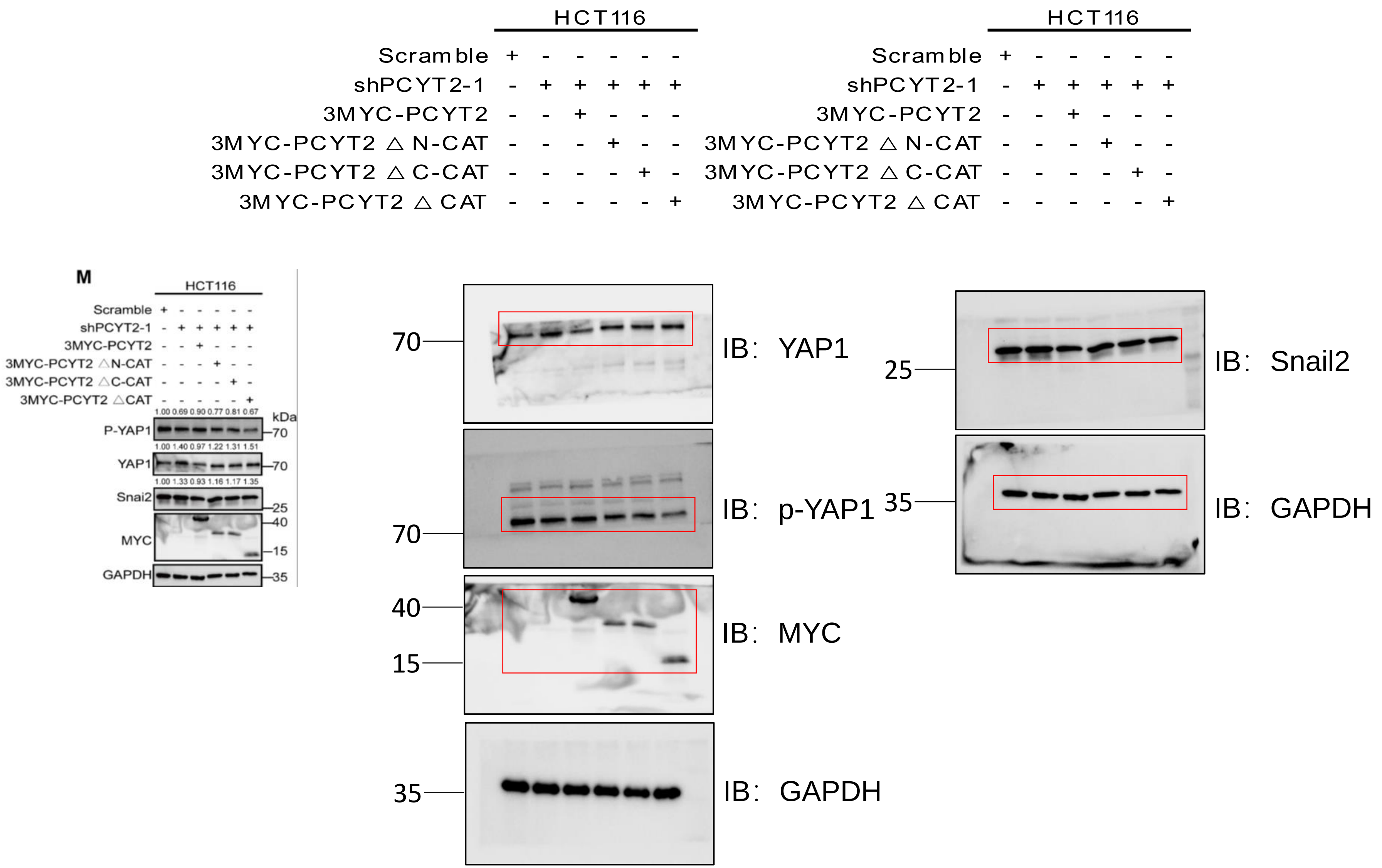

The blots come from the same batch of sample.

Full unedited gel for Figure 5 A

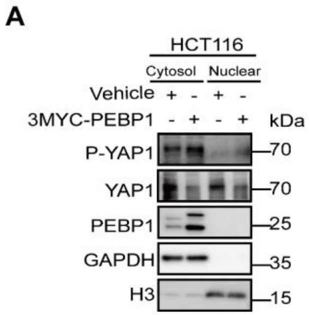

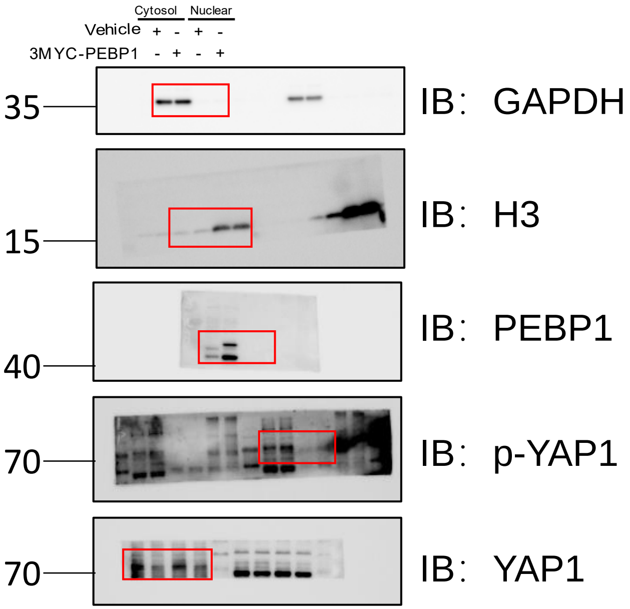

The blots come from the same batch of sample.

Full unedited gel for Figure 5 A （Biological duplicate data）

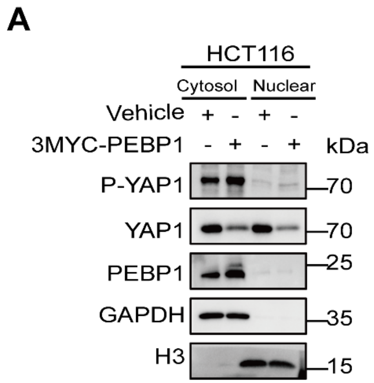

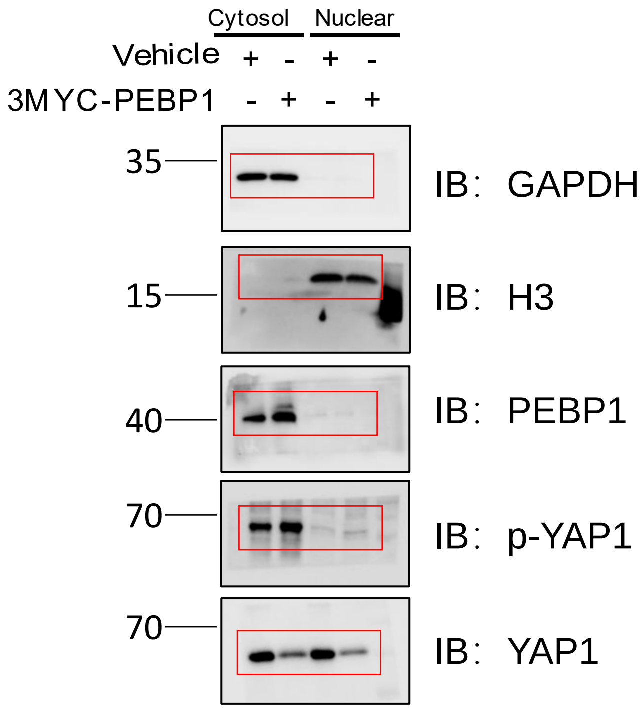

The blots come from the same batch of sample.

Full unedited gel for Figure 5 B

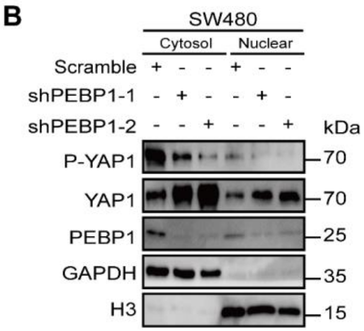

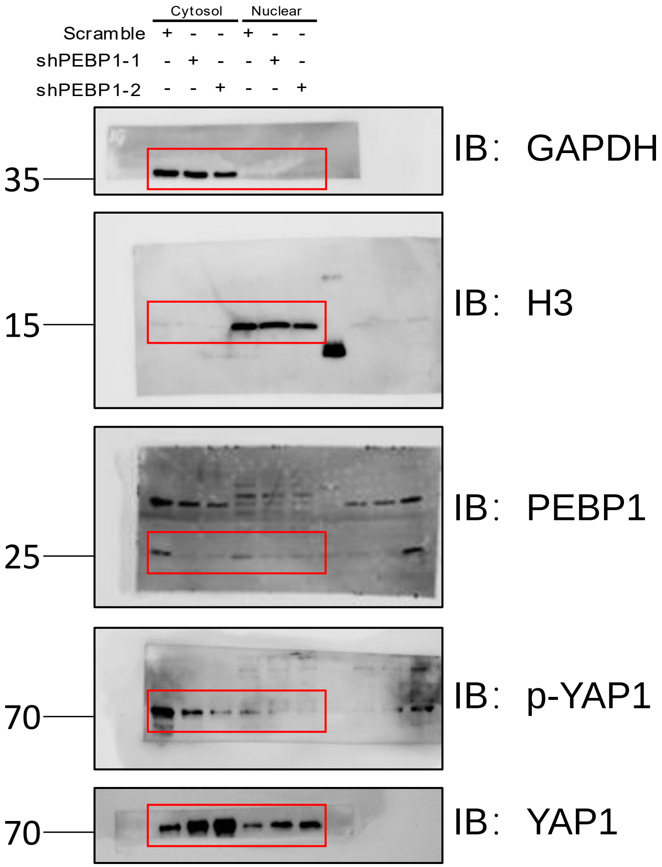

The blots come from the same batch of sample.

Full unedited gel for Figure 5 B （Biological duplicate data）

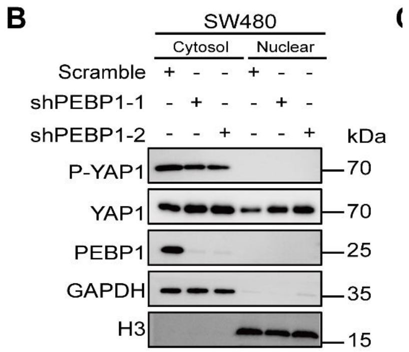

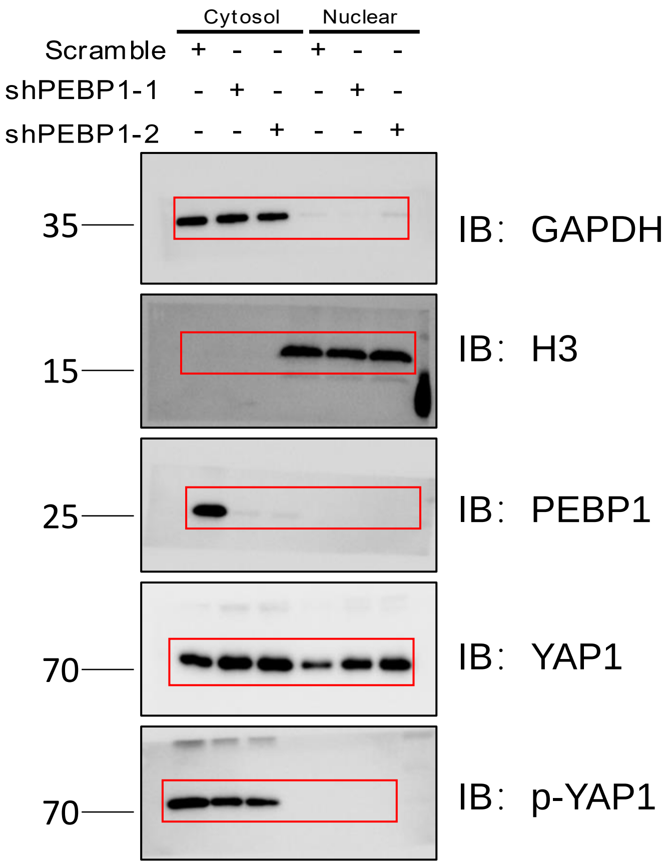

The blots come from the same batch of sample.

Full unedited gel for Figure 5 G

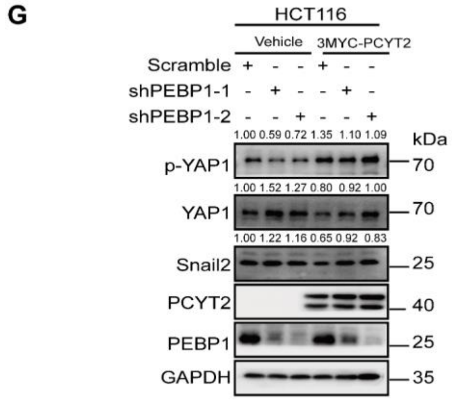

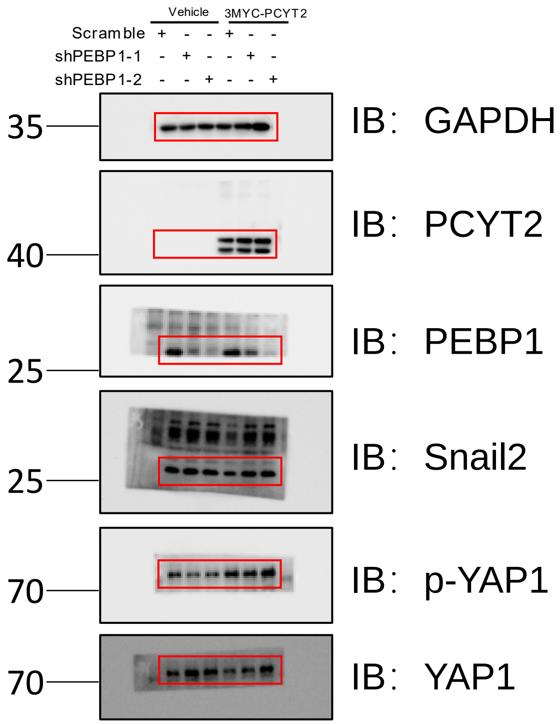

The blots come from the same batch of sample.

Full unedited gel for Figure 5I

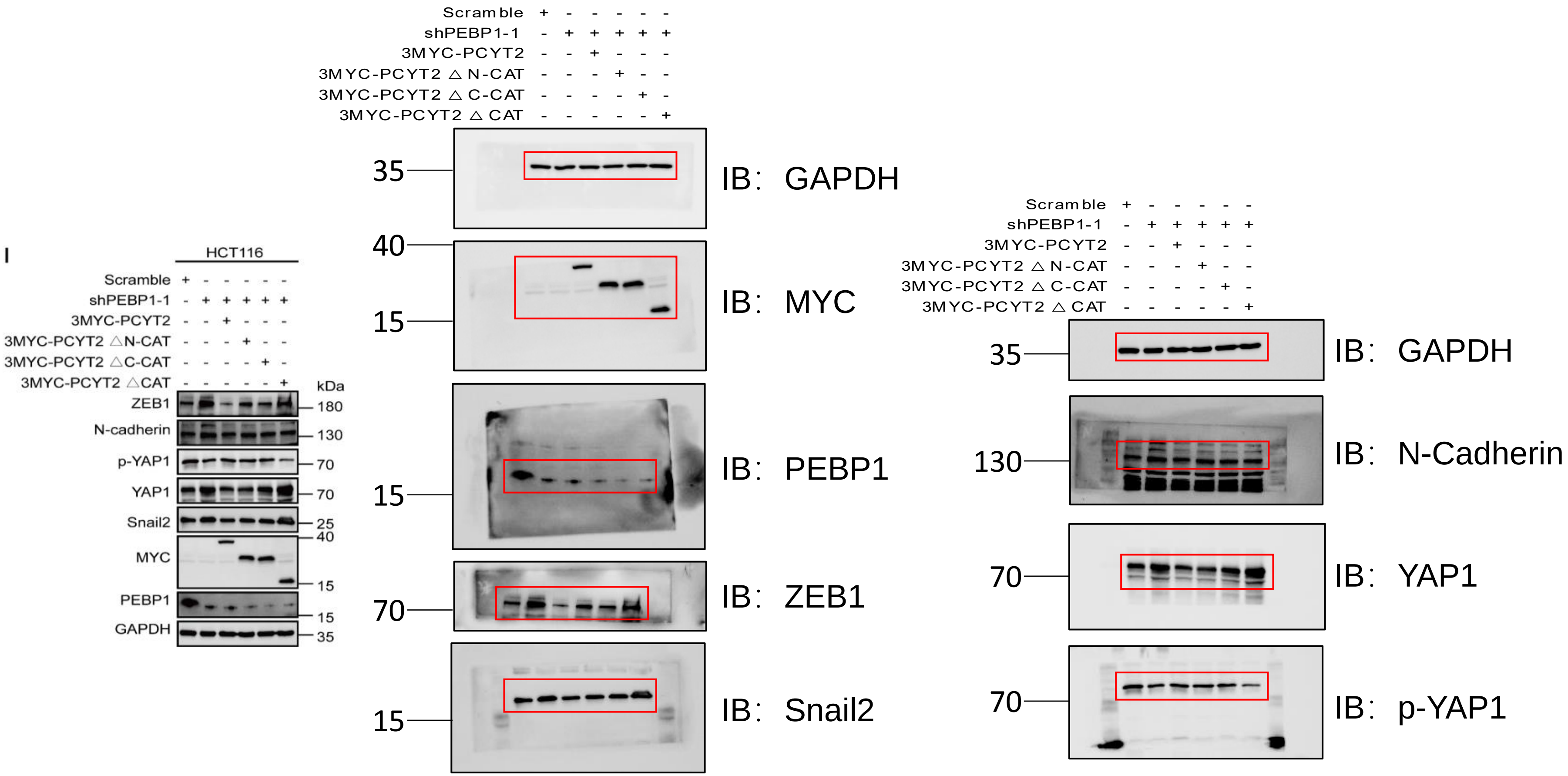

The blots come from the same batch of sample.

Full unedited gel for Figure 6A

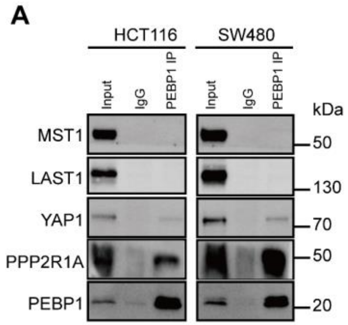

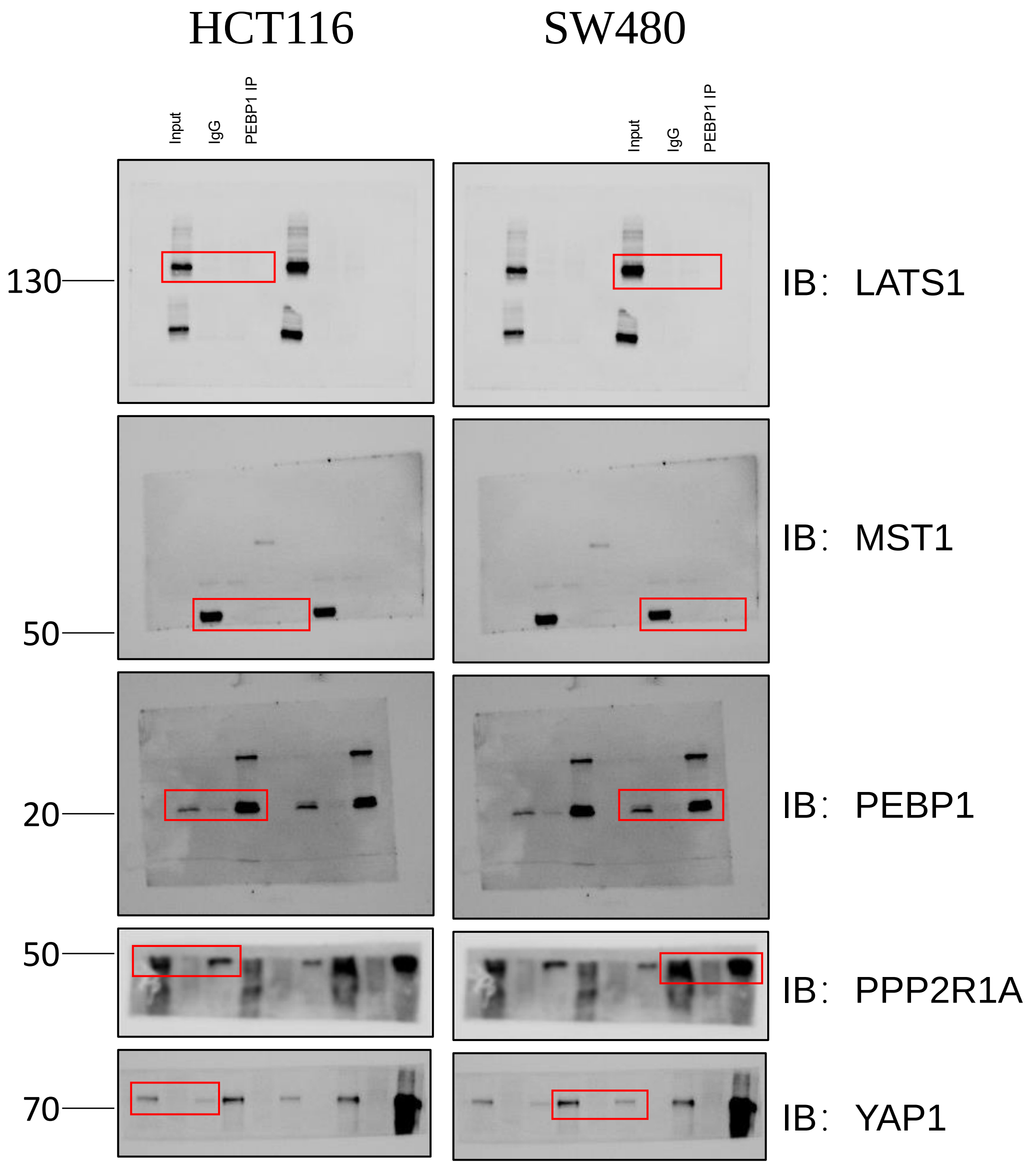

The blots come from the same batch of sample.

Full unedited gel for Figure 6A （Biological duplicate data）

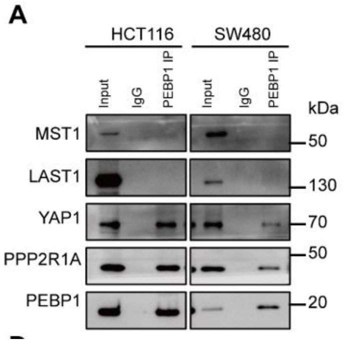

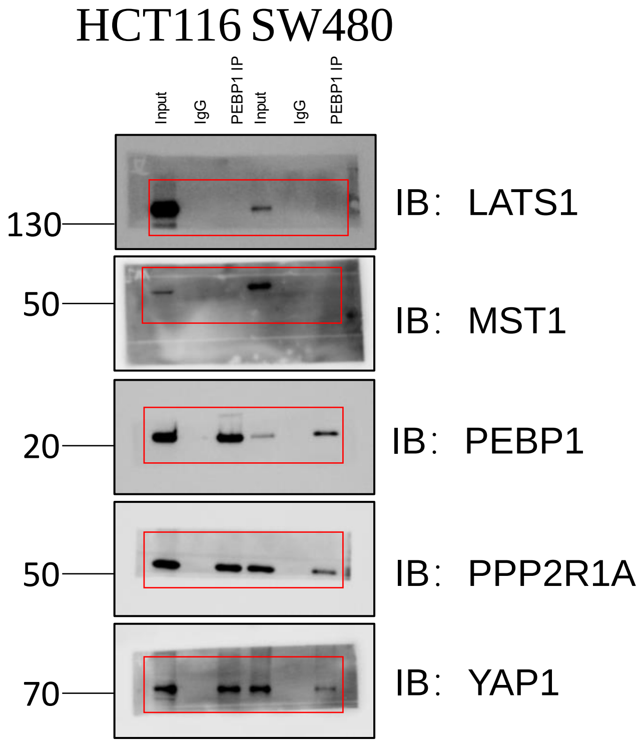

The blots come from the same batch of sample.

Full unedited gel for Figure 6B

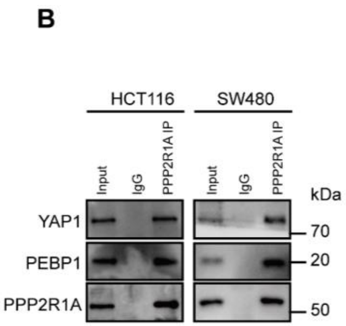

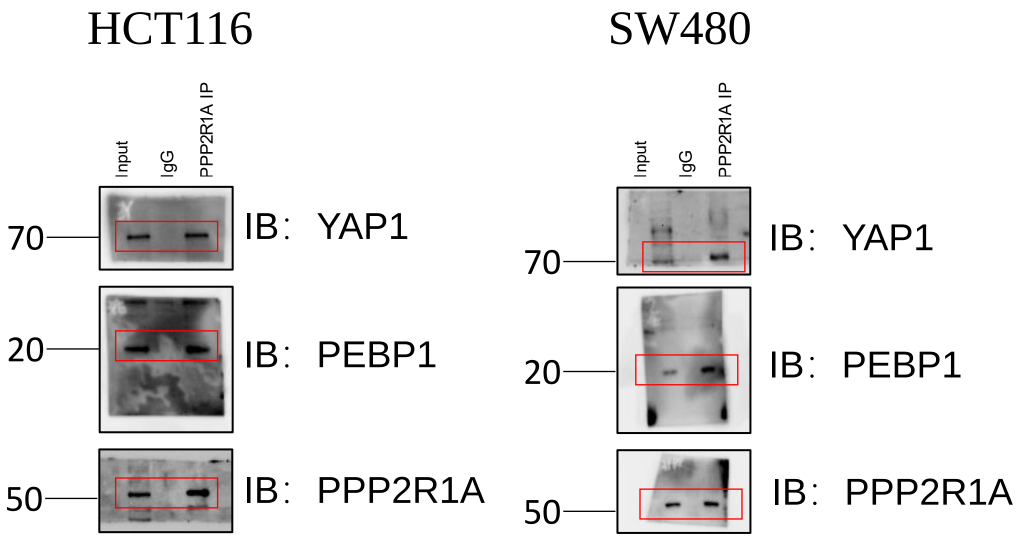

The blots come from the same batch of sample.

Full unedited gel for Figure 6B （Biological duplicate data）

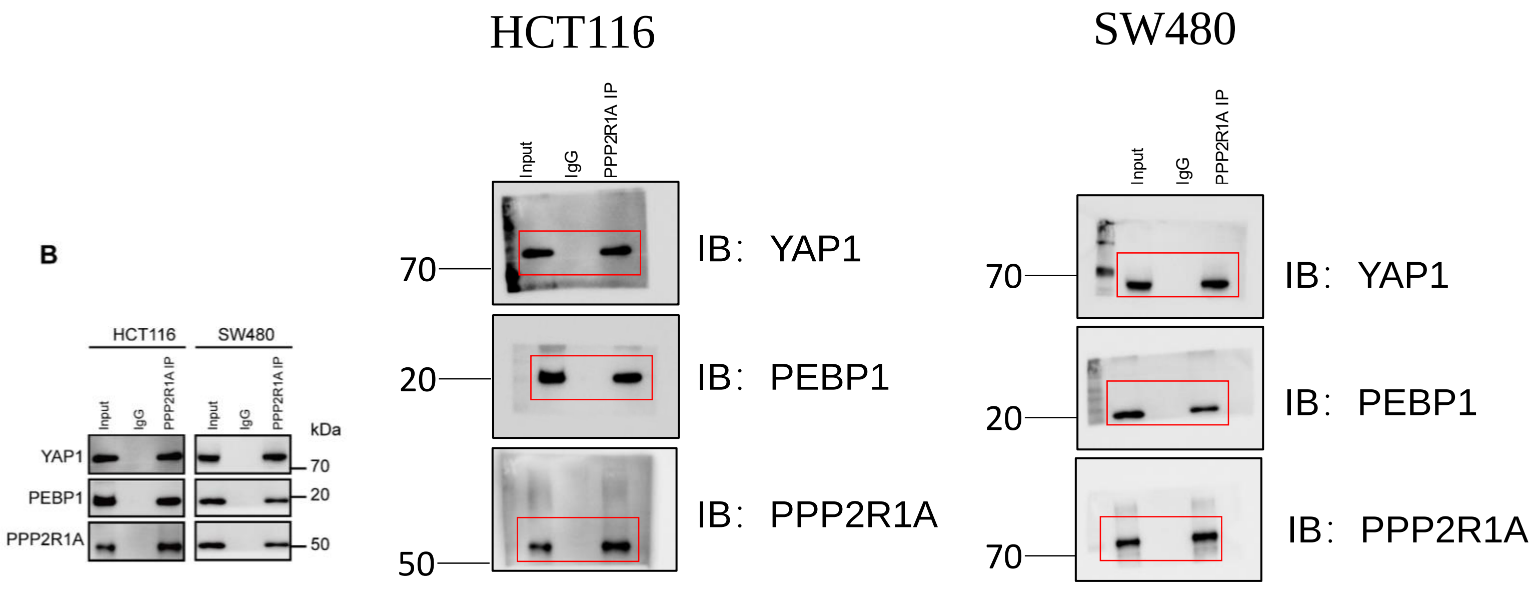

The blots come from the same batch of sample.

Full unedited gel for Figure 6C

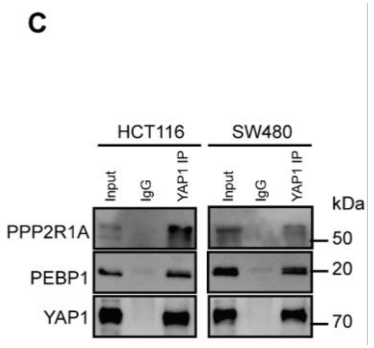

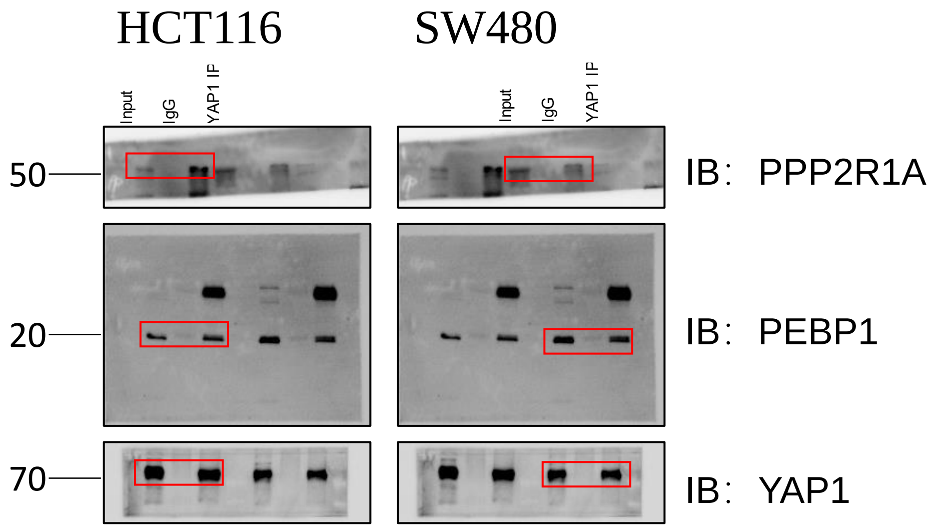

The blots come from the same batch of sample.

Full unedited gel for Figure 6C （Biological duplicate data）

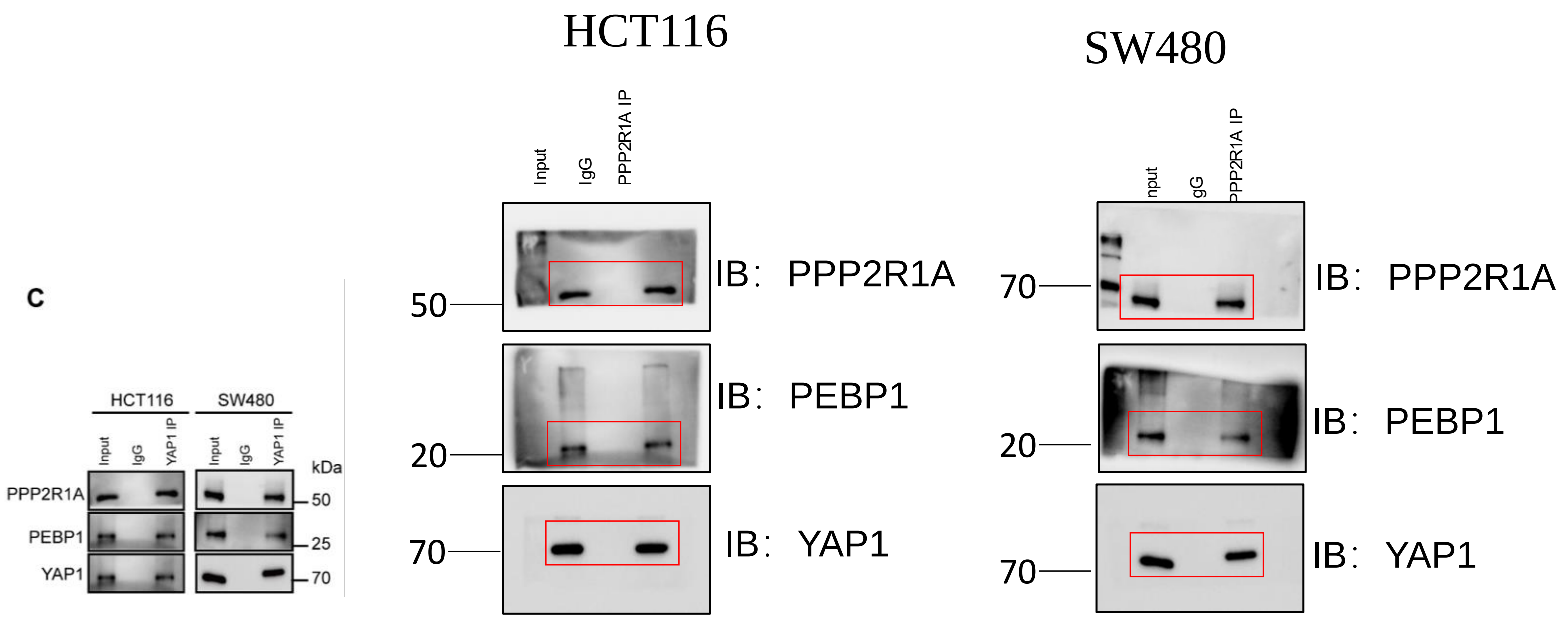

The blots come from the same batch of sample.

Full unedited gel for Figure 6F

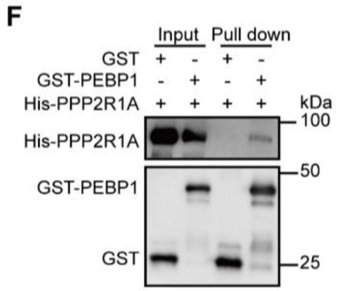

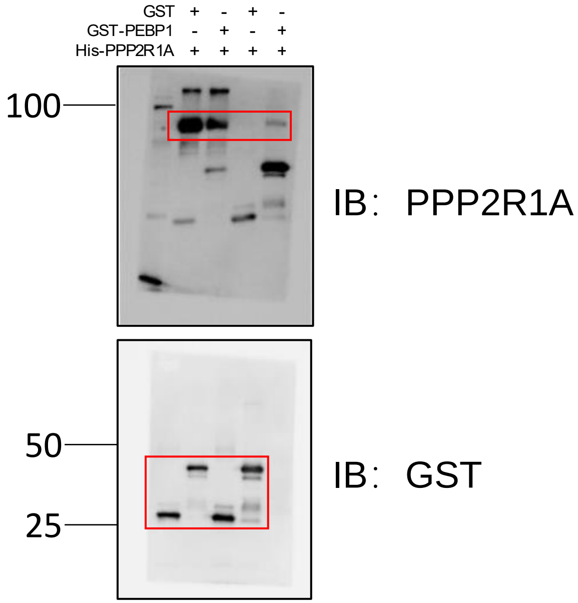

The blots come from the same batch of sample.

Full unedited gel for Figure 6G

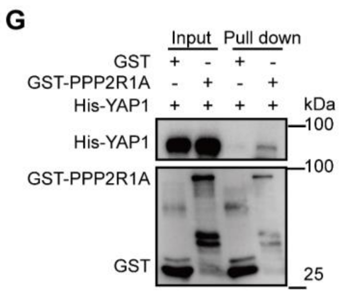

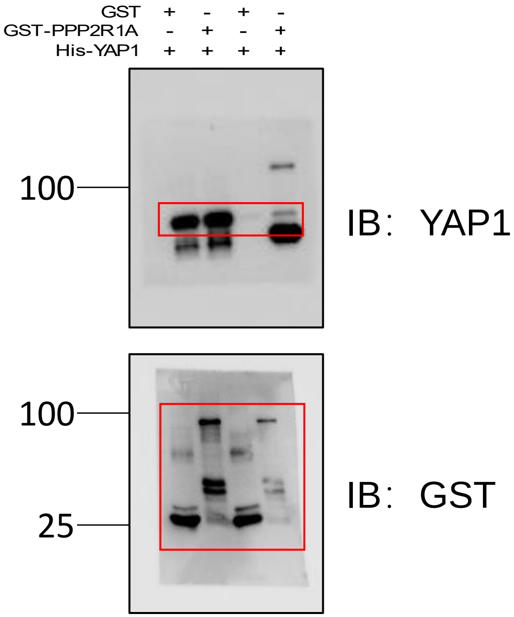

The blots come from the same batch of sample.

Full unedited gel for Figure 6H

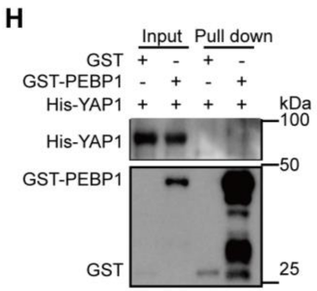

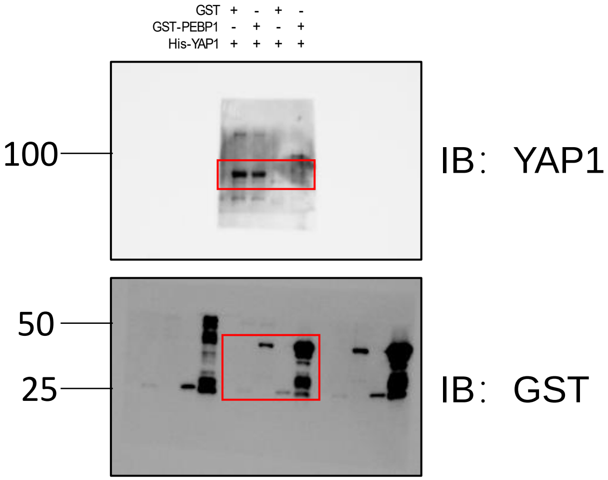

The blots come from the same batch of sample.

Full unedited gel for Figure 6I

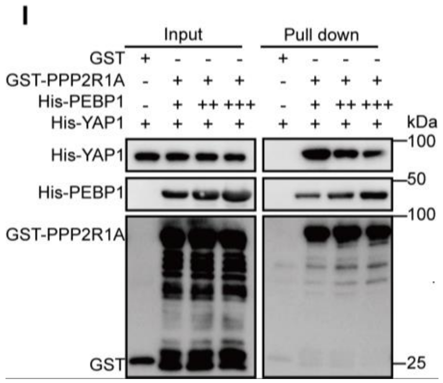

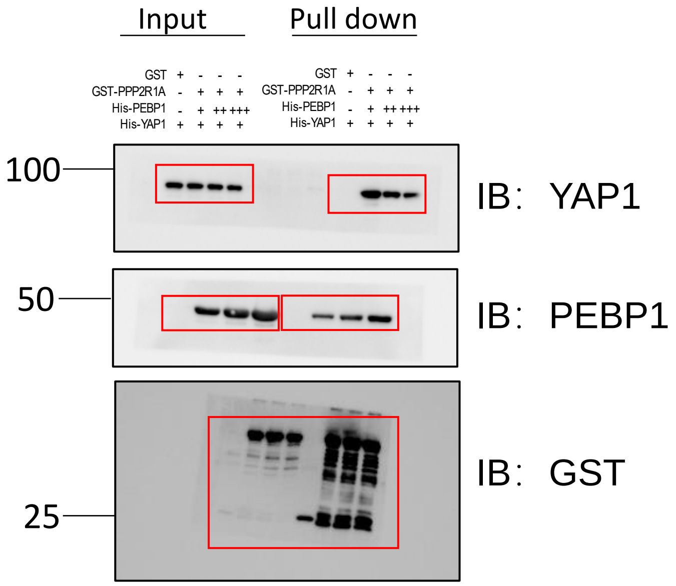

The blots come from the same batch of sample.

Full unedited gel for Figure 6I （Biological duplicate data）

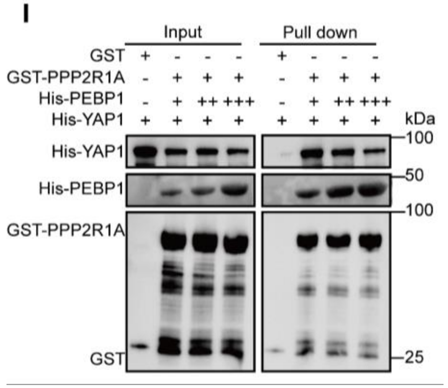

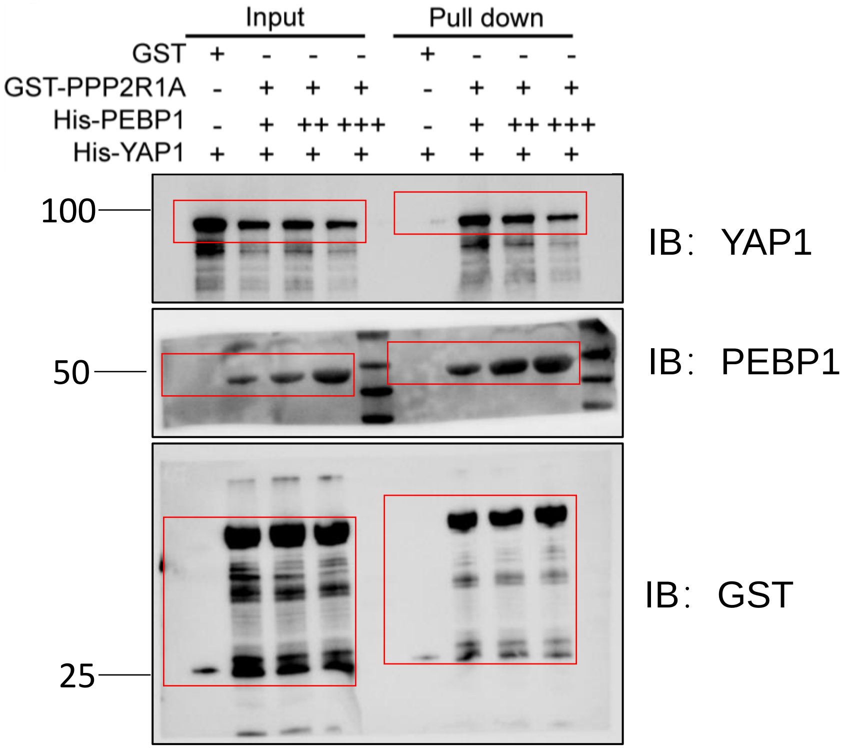

The blots come from the same batch of sample.

Full unedited gel for Figure 6J

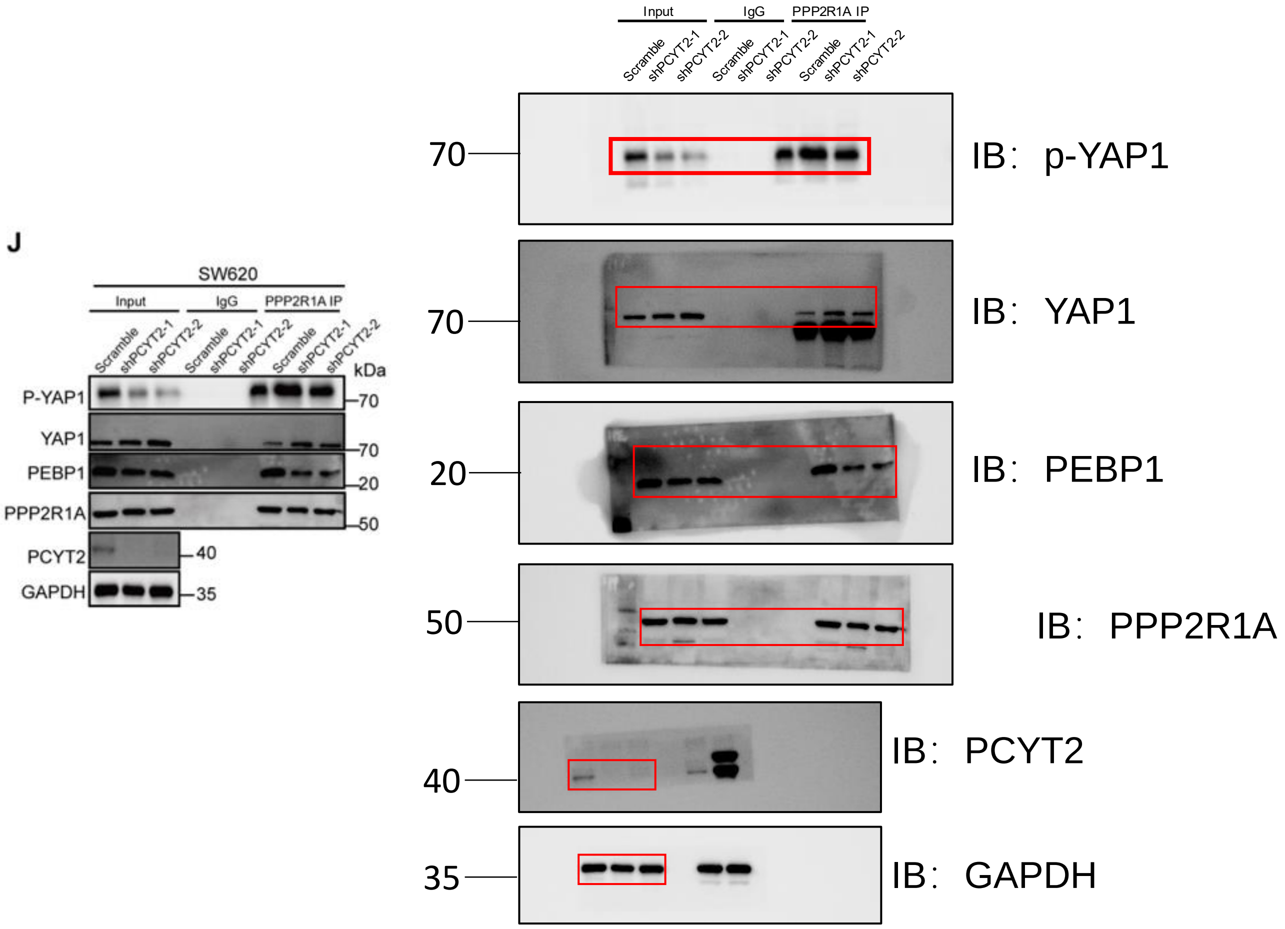

The blots come from the same batch of sample.

Full unedited gel for Figure 6K

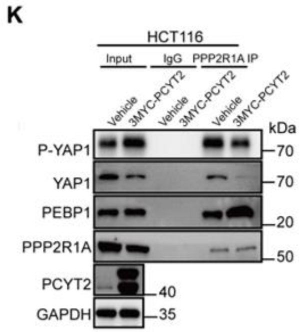

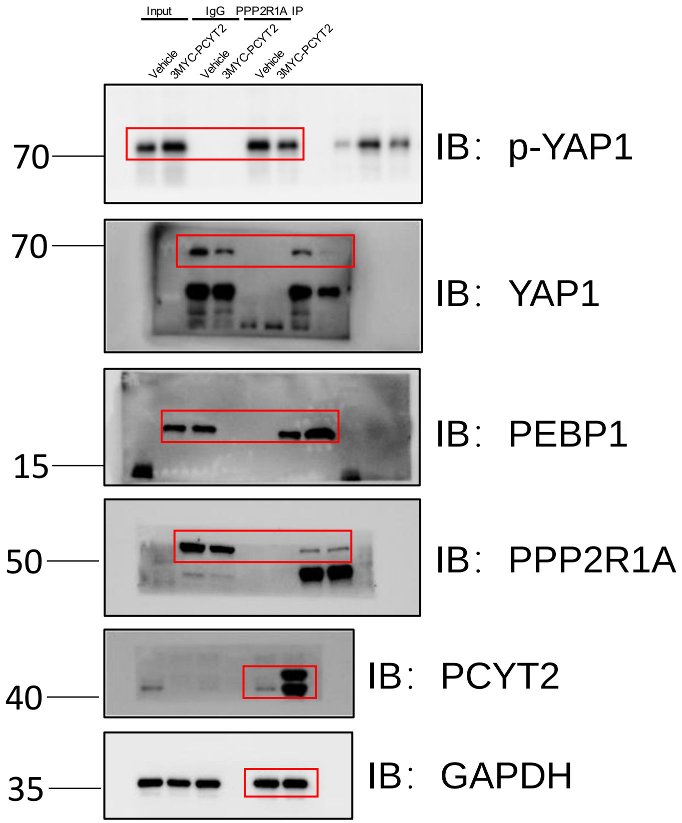

The blots come from the same batch of sample.

Full unedited gel for Figure 7A

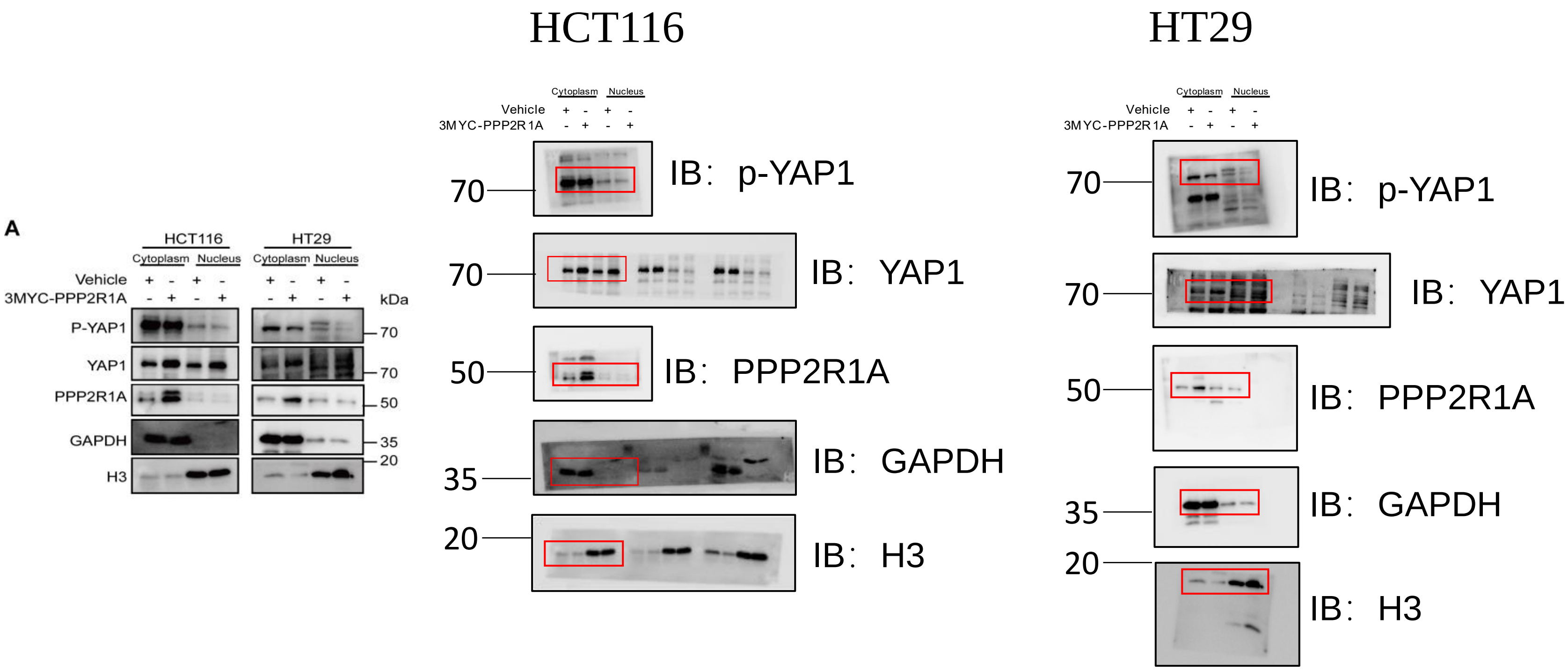

The blots come from the same batch of sample.

Full unedited gel for Figure 7A （Biological duplicate data）

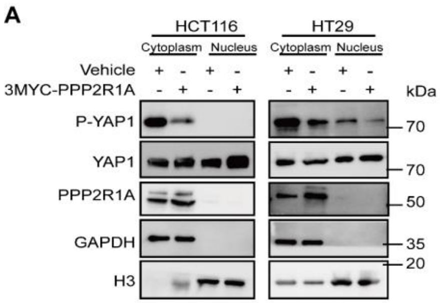

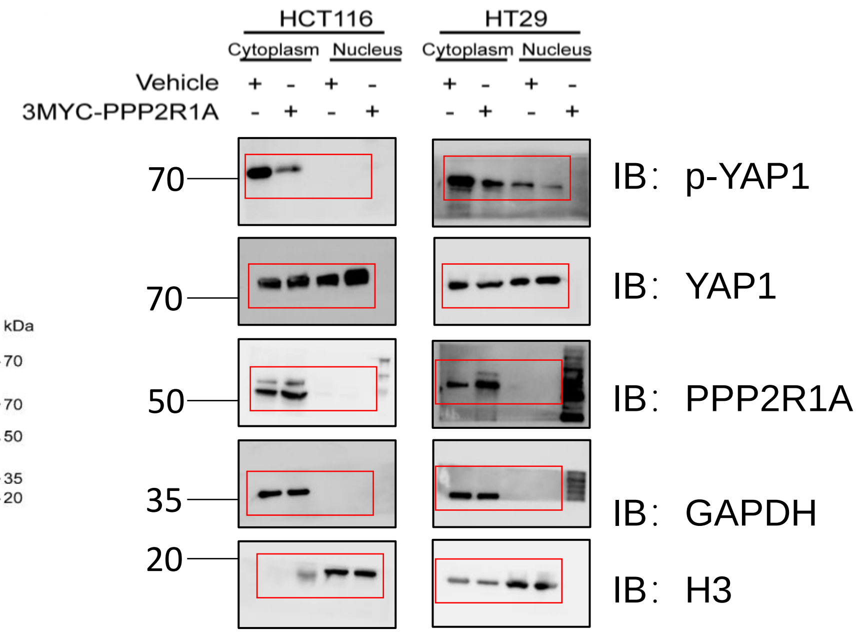

The blots come from the same batch of sample.

Full unedited gel for Figure 7B

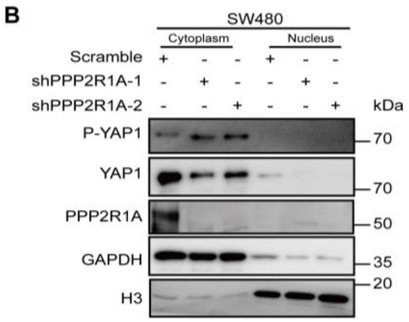

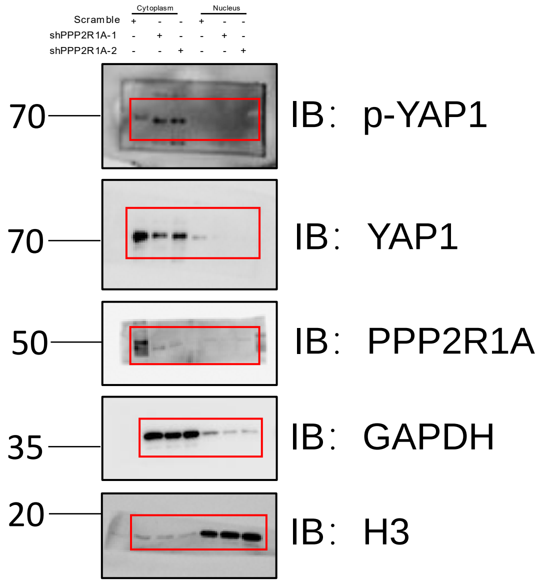

The blots come from the same batch of sample.

Full unedited gel for Figure 7G

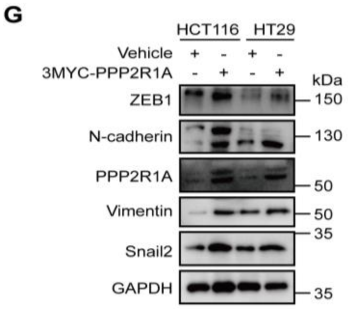

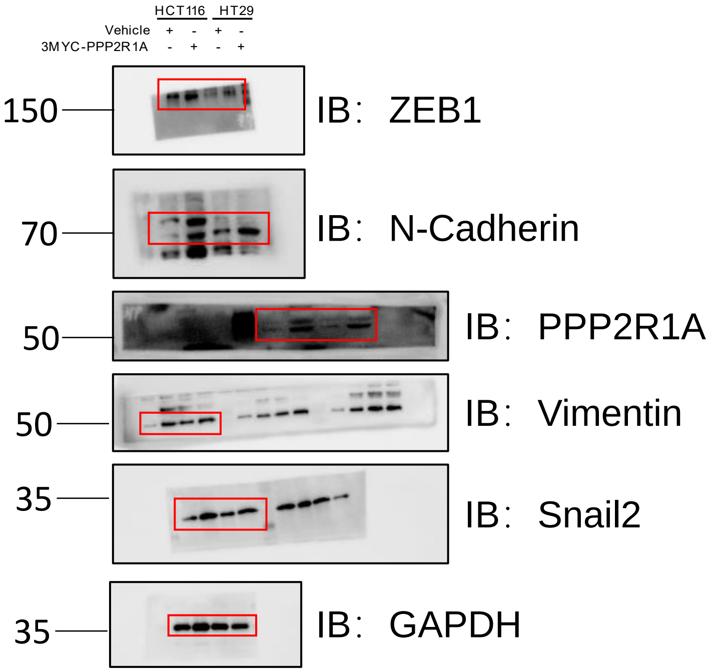

The blots come from the same batch of sample.

Full unedited gel for Figure 7G （Biological duplicate data）

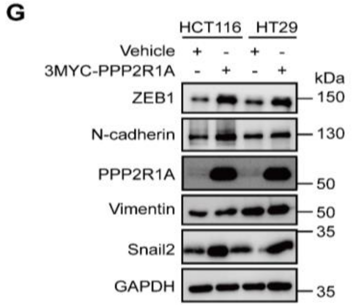

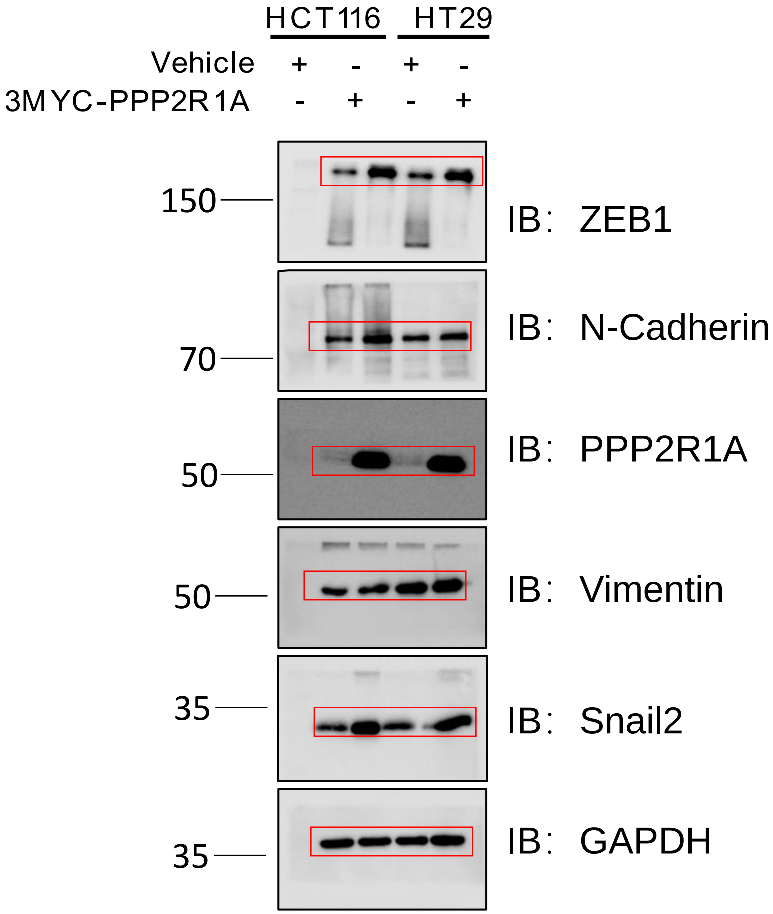

The blots come from the same batch of sample.

Full unedited gel for Figure S2 A

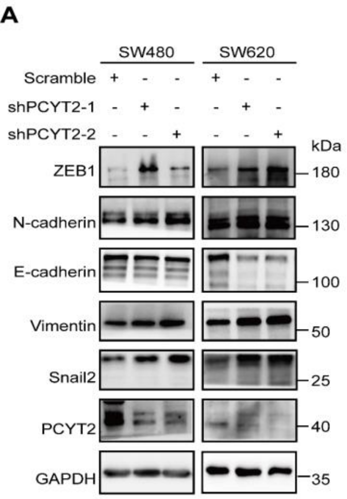

SW480

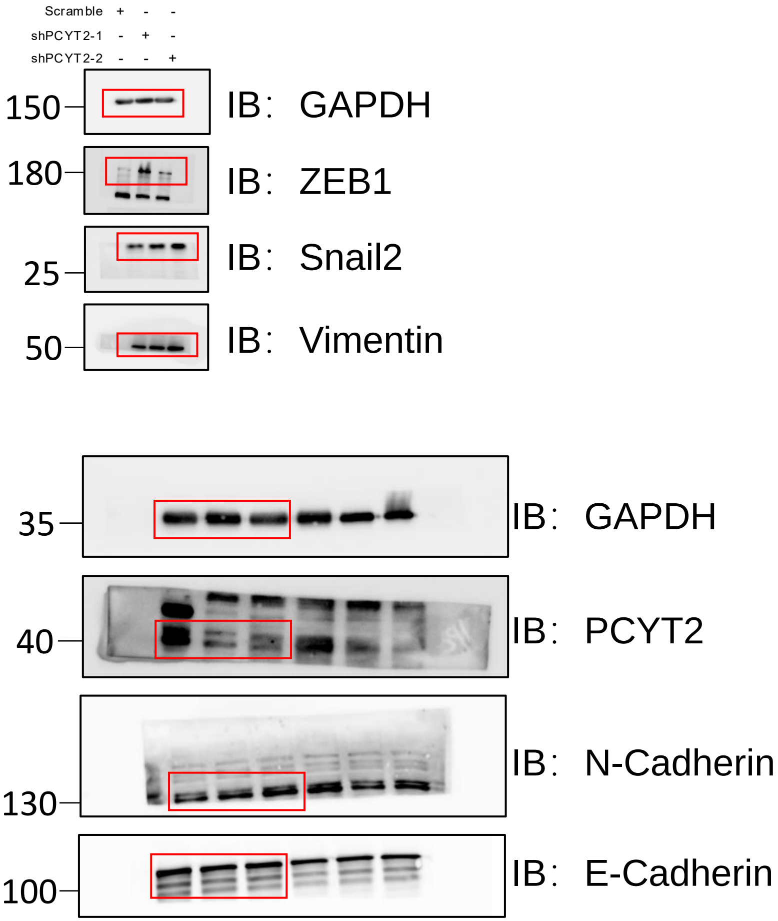

The blots come from the same batch of sample.

SW620

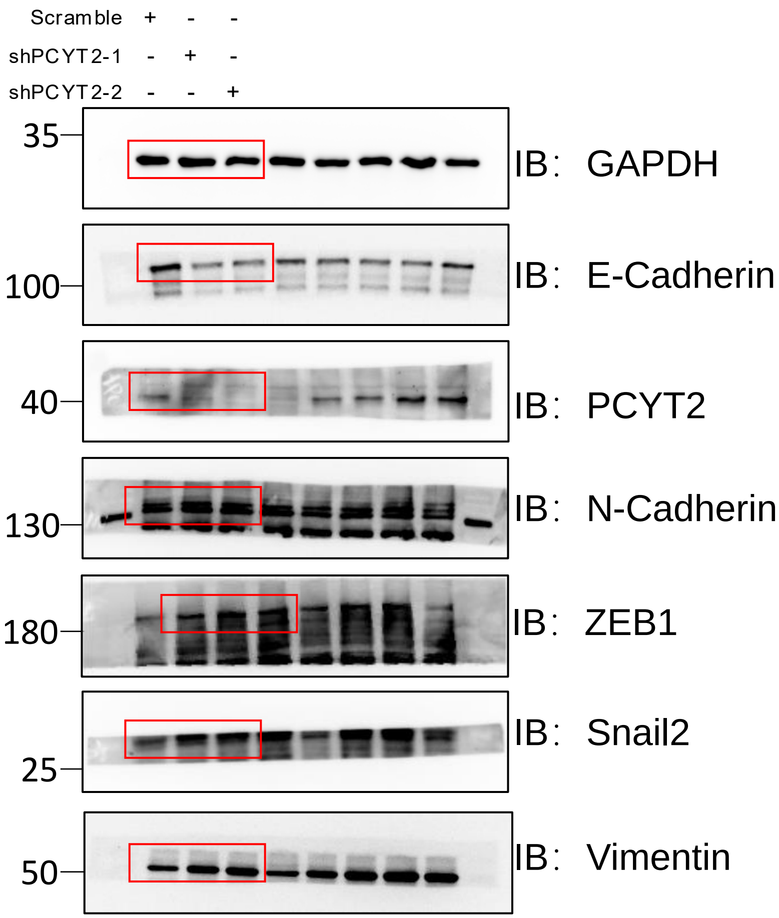

The blots come from the same batch of sample.

Full unedited gel for Figure S5 A

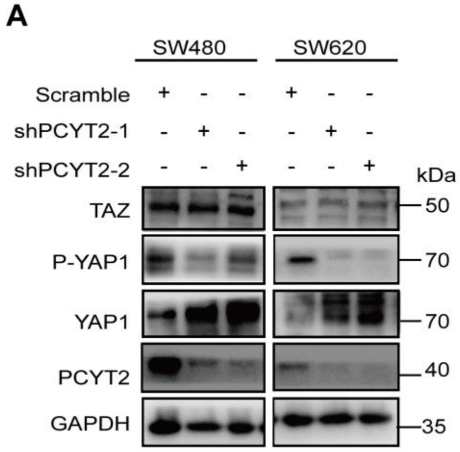

SW480 SW620

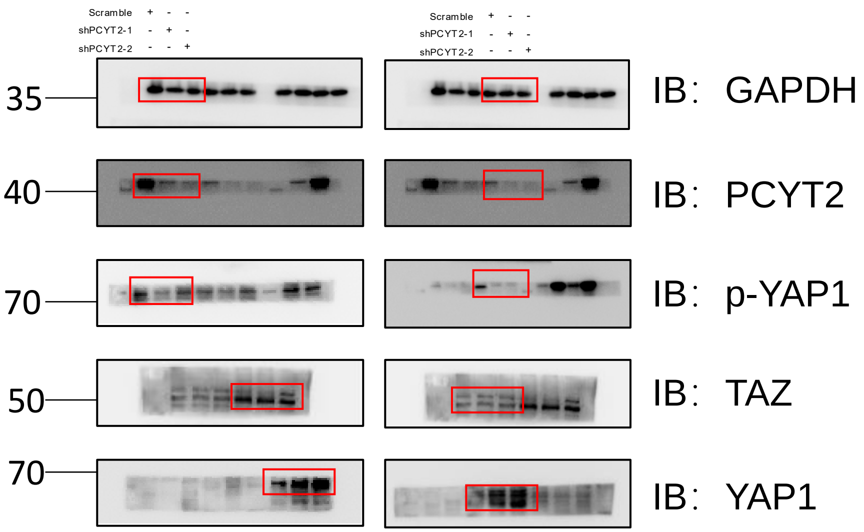

The blots come from the same batch of sample.

Full unedited gel for Figure S5B

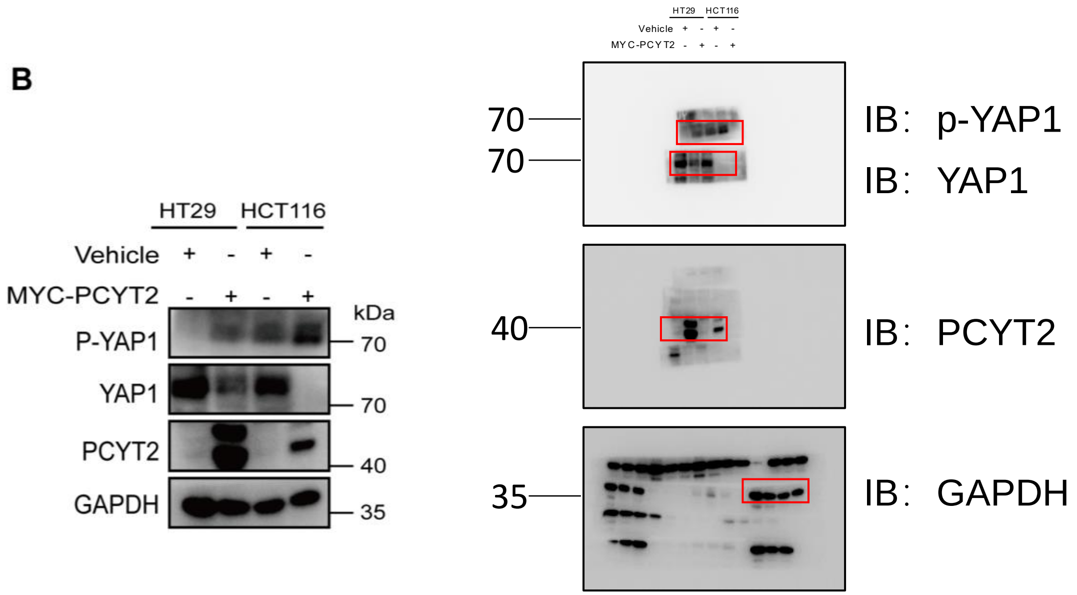

The blots come from the same batch of sample.

Full unedited gel for Figure S5C

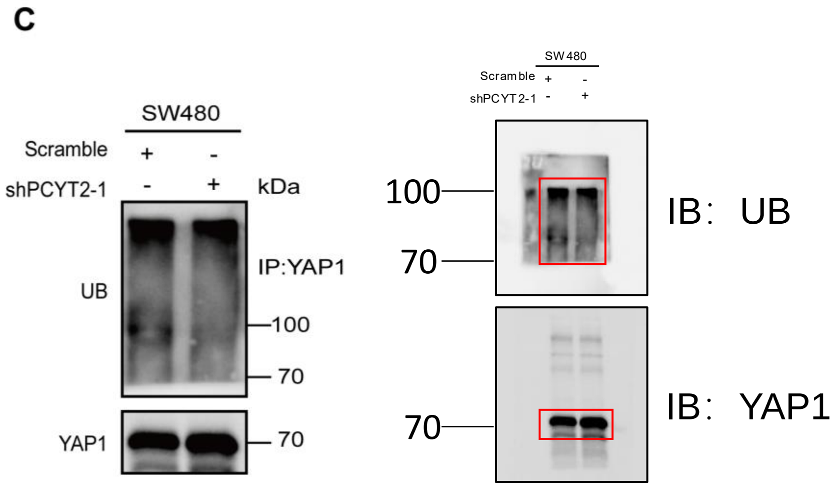

The blots come from the same batch of sample.

Full unedited gel for Figure S5D

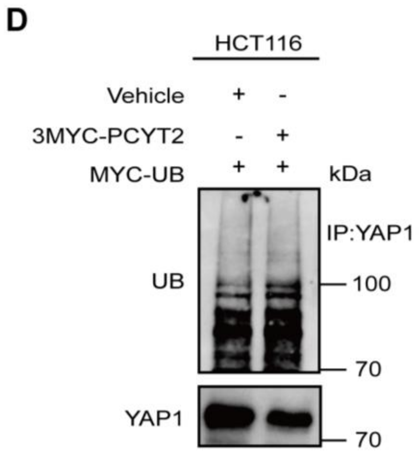

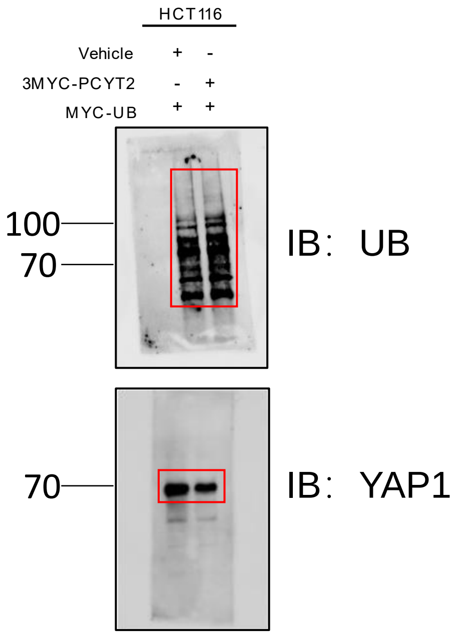

The blots come from the same batch of sample.

Full unedited gel for Figure S5E

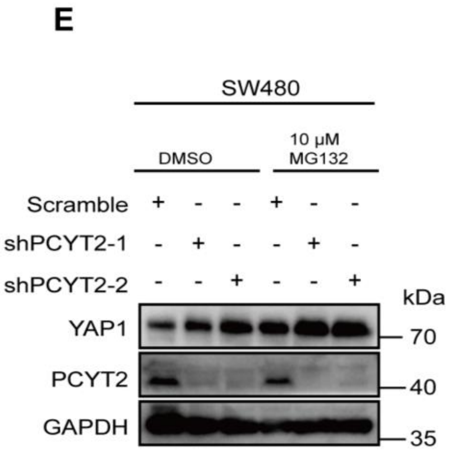

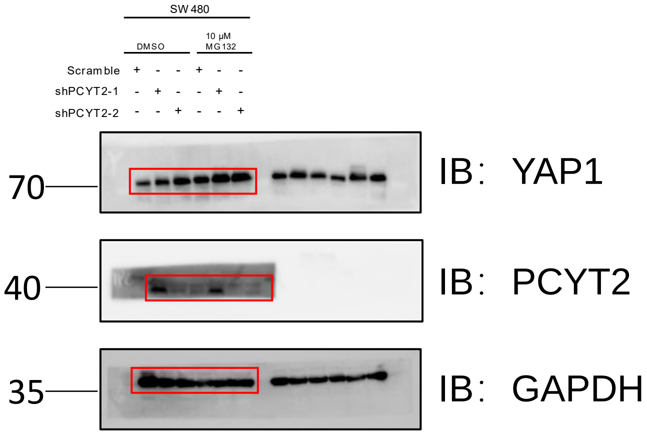

The blots come from the same batch of sample.

Full unedited gel for Figure S6A

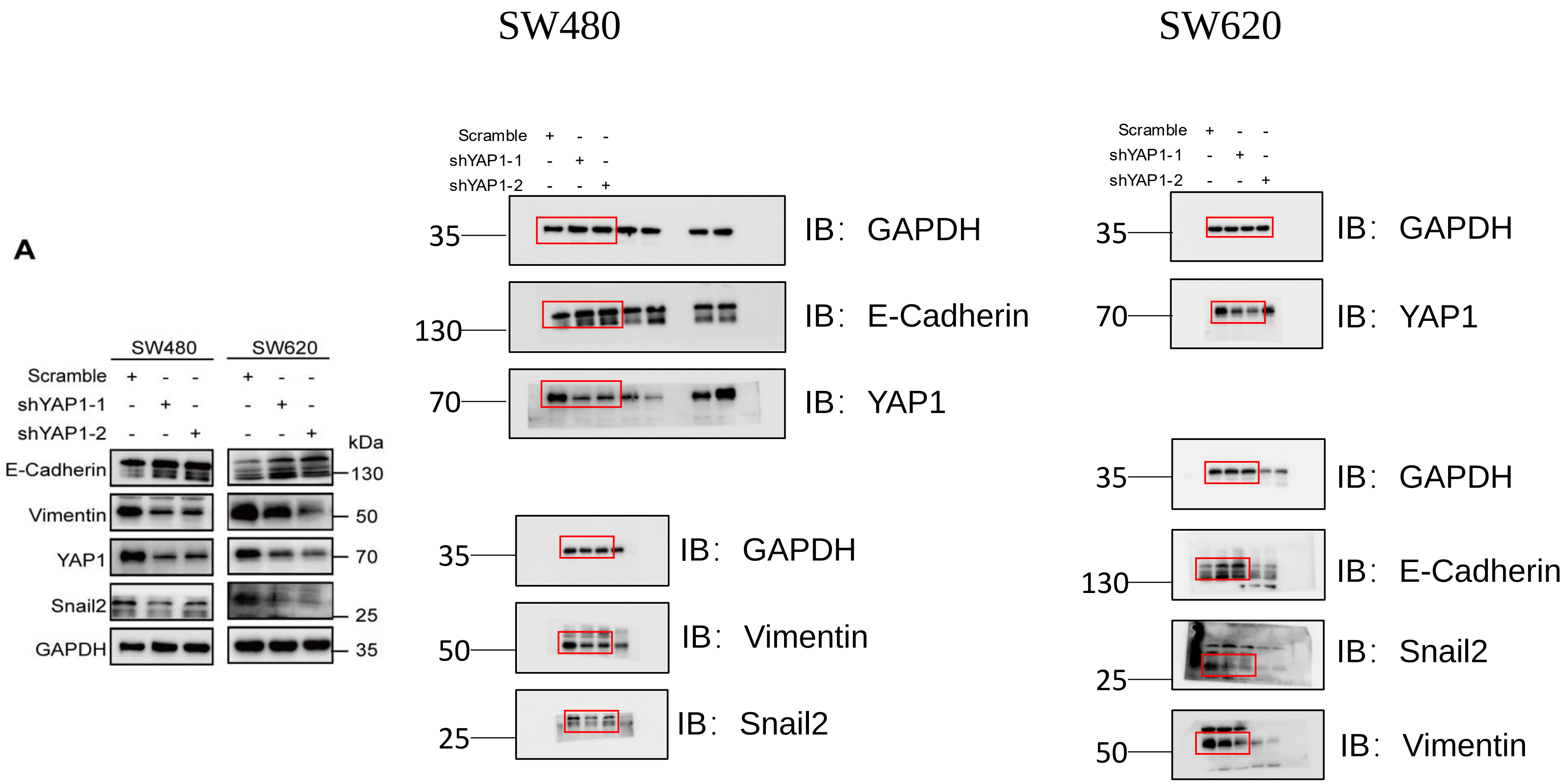

The blots come from the same batch of sample.

Full unedited gel for Figure S6B

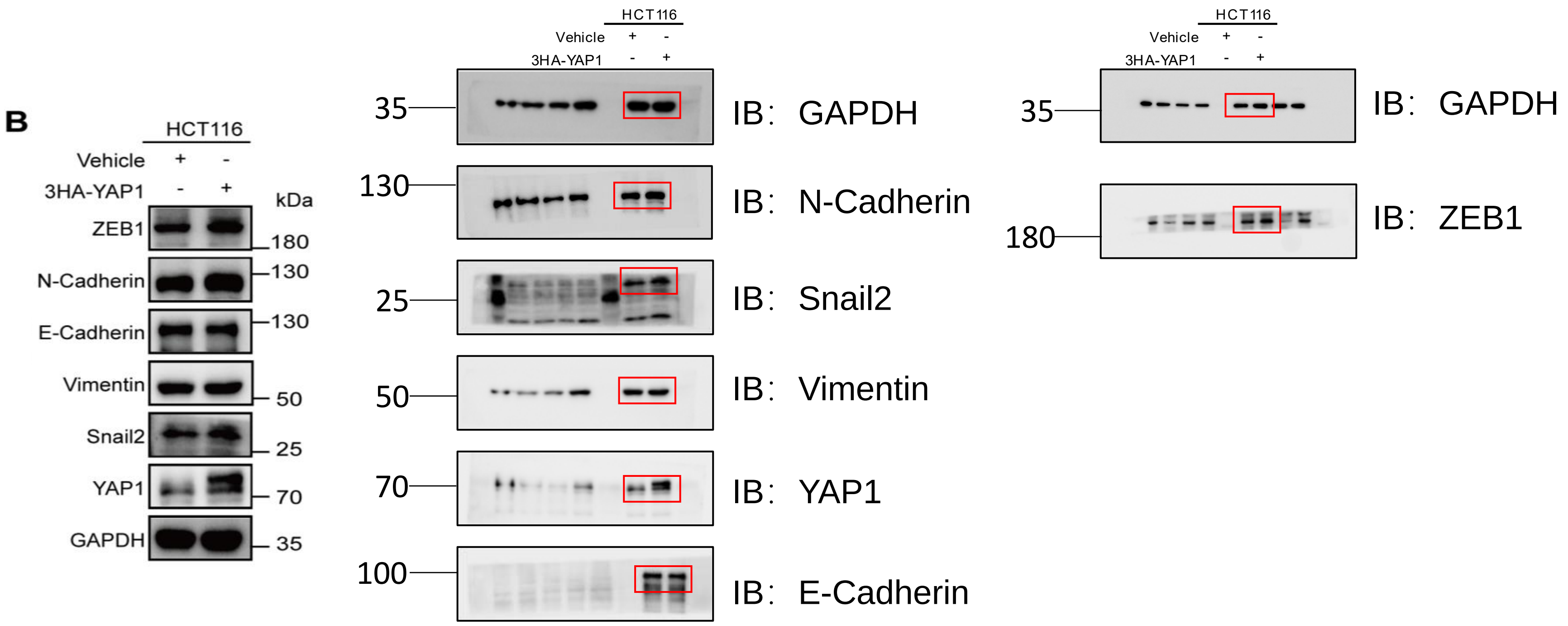

The blots come from the same batch of sample.

Full unedited gel for Figure S7A

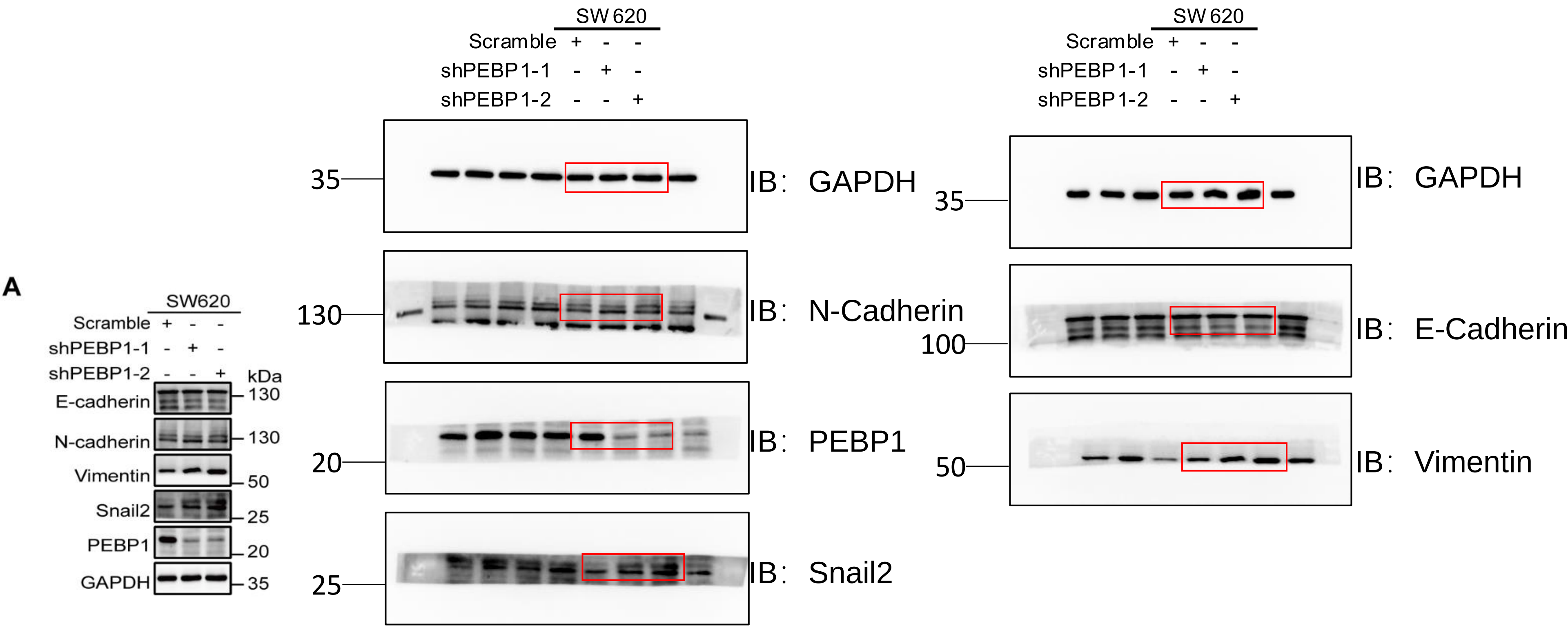

The blots come from the same batch of sample.

Full unedited gel for Figure S7B

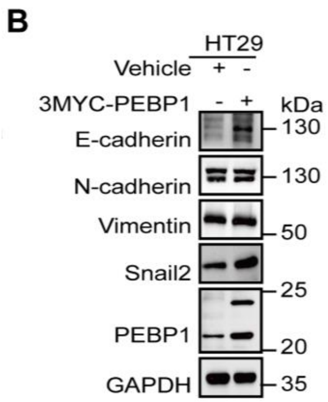

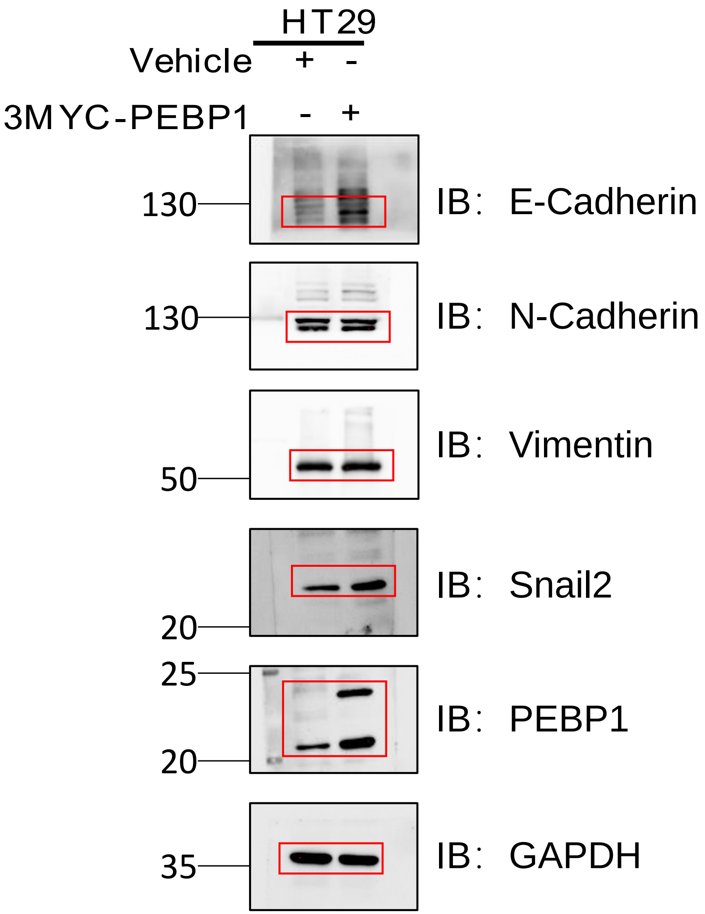

The blots come from the same batch of sample.
